# Hypoxia-induced circSPECC1 drives temozolomide resistance in glioblastoma via IGF2BP2-mediated PGK1 mRNA stabilization

**DOI:** 10.1038/s41419-026-08879-8

**Published:** 2026-06-17

**Authors:** Yu Zeng, Liqian Zhao, Tianshi Que, Li Zhou, Xiaosheng Yang, Xin Xu, Kaihua Cao, Xizhao Wang, Xuhui Wang, Wenchuan Zhang, Ming Chen

**Affiliations:** 1 Department of Neurosurgery, Shanghai Ninth People’s Hospital, Shanghai Jiao Tong University School of Medicine, Shanghai, China; 2 Institute of Translational Medicine, Zhejiang University School of Medicine, Hangzhou, China; 3 Department of Neurosurgery, Nanfang Hospital, Southern Medical University, Guangzhou, China; 4 Department of Operating Room, Xinhua Hospital, Shanghai Jiao Tong University School of Medicine, Shanghai, China; 5 Department of Neurosurgery, The First Hospital of Quanzhou Affiliated to Fujian Medical University, Quanzhou, China; 6 Department of Neurosurgery, Xinhua Hospital, Shanghai Jiao Tong University School of Medicine, Shanghai, China; 7 Department of Neurosurgery, Xinhua Hospital Affiliated to Shanghai JiaoTong University School of Medicine, Chongming Branch, Shanghai, China

**Keywords:** CNS cancer, Cancer therapeutic resistance

## Abstract

Glioblastoma (GBM) is a notoriously lethal brain tumor, primarily owing to its inevitable resistance to temozolomide (TMZ), a frontline chemotherapy. Hypoxia-driven metabolic adaptations have been implicated in therapeutic failure; however, the role of circular RNAs remains largely underexplored. By integrating multiomics profiling with functional assays in patient-derived GBM cells, orthotopic xenografts, and clinical specimens, this study aimed to elucidate the role of hypoxia-induced hsa_circ_0000745 (circSPECC1) in mediating TMZ resistance. Mechanistic investigation included RNA pulldown, RIP, glycolysis flux analysis, and DNA damage assessment. The circSPECC1 is overexpressed in GBM and correlates with poor prognosis. Hypoxia triggers HIF-1α-mediated transcriptional upregulation of circSPECC1, which scaffolds insulin-like growth factor 2 mRNA-binding protein 2 (IGF2BP2) to stabilize phosphoglycerate kinase 1 (PGK1) mRNA. Importantly, circSPECC1/PGK1 axis activation enhances glycolytic flux, blunts TMZ-induced DNA damage, and confers chemoresistance. Targeting circSPECC1 disrupts PGK1-driven glycolysis, restores TMZ sensitivity, and synergizes with TMZ to extend survival in orthotopic GBM models. In conclusion, this study identifies a previously uncharacterized HIF-1α/circSPECC1/IGF2BP2/PGK1 axis that drives metabolic adaptation and TMZ resistance in GBM. Targeting this axis overcomes acquired chemoresistance, positioning circSPECC1 as both a prognostic biomarker and a therapeutic vulnerability in hypoxic GBM niches.

**Figure:**
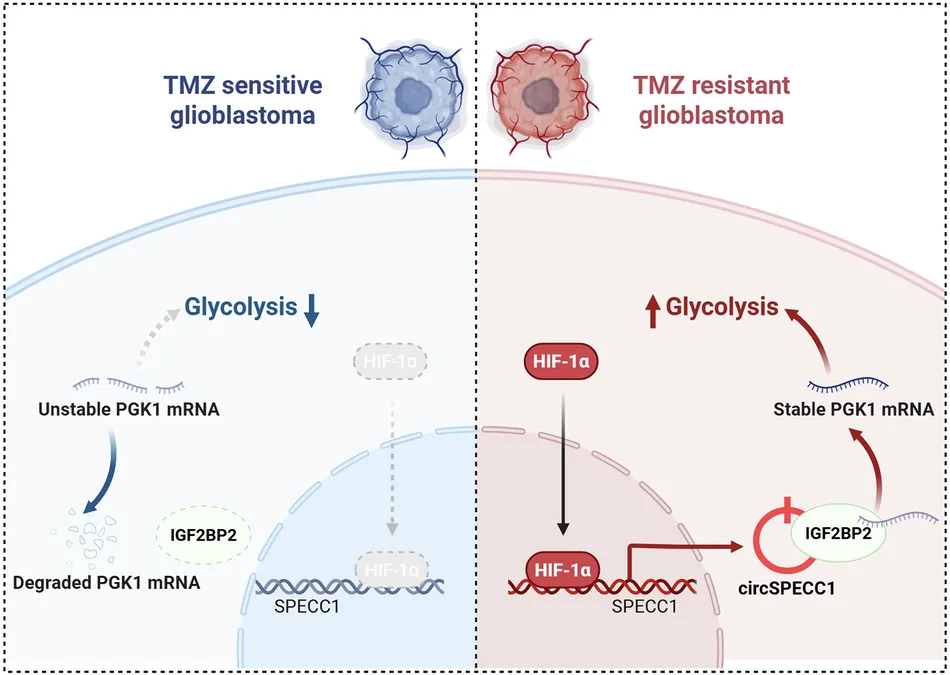

## Introduction

Glioblastoma (GBM) is the most prevalent and aggressive primary brain tumor in adults, accounting for approximately 49% of all malignant brain tumors [1]. GBM is characterized by rapid proliferation, diffuse invasion of the surrounding brain tissue, and a high propensity for recurrence owing to treatment resistance, presenting significant therapeutic challenges [2]. The Stupp regimen is the current standard of care and involves maximal safe surgical resection followed by concurrent radiotherapy and adjuvant temozolomide (TMZ) [3]. Several clinical trials have demonstrated that combined strategies based on the Stupp regimen can modestly improve survival in patients [4–7]; however, limited efficacy and frequent tumor recurrence owing to TMZ resistance persist as critical therapeutic barriers.

Recent advances in molecular biology have highlighted the pivotal role of non-coding RNAs, particularly circular RNAs (circRNAs), in cancer pathogenesis [8–10]. CircRNAs are single-stranded RNA molecules with a covalently closed-loop structure that confers exceptional stability and resistance to exonuclease degradation [11]. Their tissue- and developmental stage-specific expression patterns make them promising candidates for diagnostic and prognostic biomarkers [12]. CircRNAs are involved in oncogenic processes through various mechanisms, including acting as sponges for microRNAs (miRNAs), modulating protein interactions, regulating parental gene transcription, and even encoding functional proteins [13, 14]. Their impact on cancer progression and treatment remains debatable and sometimes contradictory in a context-dependent manner.

Among these, hsa_circ_0000745 (circSPECC1) has emerged as a significant oncogenic driver in gliomas. Our previous study established that circSPECC1 is significantly upregulated under hypoxic conditions, and that its depletion suppresses cell proliferation and epithelial-mesenchymal transition [15]. Similarly, Lan et al. reported that circSPECC1 functions as a molecular sponge for miR-615-5p, thereby suppressing huntingtin-interacting protein-1 and activating the AKT signaling pathway [16]. The functional landscape of circSPECC1 appears to be multifaceted. Although its non-coding transcript promotes tumor progression, its recently identified coding potential for the peptide SPECC1-415aa has been reported to inhibit glioma recurrence [17]. To address these gaps, this study aimed to define the functional significance of circSPECC1 in hypoxia-induced TMZ resistance. We demonstrated that hypoxia-induced circSPECC1 formed a functional complex with insulin-like growth factor 2 mRNA-binding protein 2 (IGF2BP2) to stabilize phosphoglycerate kinase 1 (PGK1) mRNA. This interaction facilitates a glycolytic shift that confers TMZ resistance in GBM. These findings reveal a novel circRNA-centered axis that drives GBM metabolic adaptation and chemoresistance within the hypoxic niche, underscoring the context-dependent nature of circSPECC1 and its potential as both a prognostic marker and therapeutic target.

## Materials and methods

### Cell culture

The cells were cultured under standard conditions at 37 °C in a humidified atmosphere with 5% CO₂. Commercially available astroglia cell SVG (CRL-8621), GBM cell lines (LN229, CVCL_0393; U138, CVCL_0020; A172, CRL-1620; T98G, CRL-1690; U87MG, HTB-14) and HEK293T (CRL-3216) cells were obtained from the American Type Culture Collection (ATCC). Primary GBM cell lines (pGBM1-5) were a kind gift from Nanfang Hospital, which were all established from tumors pathologically diagnosed as WHO grade IV GBM, IDH1 wild-type, and MGMT promoter unmethylated following institutional review board approval and informed consent. Tumor tissues were enzymatically dissociated using collagenase IV (1 mg/mL; Sigma‒Aldrich) for 1 h at 37 °C, followed by mechanical disruption. GBM and HEK293T cells were cultured in DMEM (Gibco, USA) supplemented with 10% fetal bovine serum (BI, Israel) and 1% penicillin‒streptomycin at 37 °C in a 5% CO₂ humidified incubator. For the hypoxia experiments, cells were placed in a modular hypoxia chamber and exposed (1% O₂, 5% CO₂, balanced N₂) for the indicated duration, while normoxic controls were maintained at 21% O₂. All cultures were subjected to routine mycoplasma screening to confirm the absence of contamination. The cells were passaged with 0.25% trypsin-EDTA and harvested at 70–80% confluence for downstream assays.

### Clinical samples

Primary glioma tissues were snap-frozen in liquid nitrogen within 30 min of resection or collection and stored at –80 °C until nucleic acid extraction. The pathological diagnosis was confirmed by two independent neuropathologists according to the WHO criteria. Clinicopathological data are provided in Supplementary Table 2. Clinical specimens were collected under protocols approved by the institutional review boards of Xinhua Hospital (XHEC-C-2022-111-1). Written informed consent was obtained from all the participants.

### Fluorescence in situ hybridization (FISH)

RNA-FISH was performed to localize circSPECC1 using Cy3-labeled probes (BersinBio, China) targeting back-splice junctions following manufacturer’s instruction. Briefly, cells grown on poly-L-lysine-coated coverslips were fixed in 4% paraformaldehyde for 15 min, permeabilized with 0.5% Triton X-100 for 10 min and dehydrated in graded ethanol. In the case of tissue FISH staining, the tissue microarray slides were first dewaxed with xylene and ethanol solutions. Hybridization was carried out overnight at 37 °C in a humidified chamber with probe-containing buffer. For protein-RNA co-localization, cells were immunostained with primary antibodies (anti-IGF2BP2, 1:200, Proteintech 11601-1-AP) followed by Alexa Fluor 488-conjugated secondary antibodies (Invitrogen, USA). Nuclei were counterstained with DAPI (Sigma-Aldrich), and coverslips were mounted. Images were acquired using an Olympus FV3000 confocal microscope.

CircSPECC1 expression was quantified using staining index (SI) as previously reported [18], which arise from multiplying the positive staining intensity score (negative = 0; weak = 1; medium = 2; strong = 3) by the score for the percentage of positive staining cells (0: <5%; 1: 5–25%; 2: 26–50%; 3: 51–75%; 4: >75%). Samples were classified as low (SI < 6) or high (SI ≥ 6) expression. Scoring was performed independently by two blinded observers.

### Chromatin immunoprecipitation (ChIP)

ChIP assays were performed using the SimpleChIP Chromatin IP Kit (#9005; CST, USA) according to the manufacturer’s instruction. Briefly, 4 × 10⁶ cells (or 25 mg fresh tissue) were cross-linked with 1% formaldehyde (37% stock, freshly opened) for 10 min at room temperature, quenched with 0.125 M glycine, and washed twice in ice-cold PBS containing protease inhibitor cocktail (200× PIC). Chromatin was digested with micrococcal nuclease (0.5 µL per IP preparation) for 20 min at 37 °C to yield 150–900 bp fragments, as confirmed by 1% agarose gel electrophoresis. After clarification (10,000 rpm, 10 min, 4 °C), 5–10 µg chromatin was diluted 1:4 in 1× ChIP Buffer + PIC and pre-cleared with ChIP-grade protein G magnetic beads (30 µL/IP) for 2 h at 4 °C. Immunoprecipitations were carried out overnight at 4 °C with 2 µg of the specified antibody or matched normal rabbit IgG. Beads were collected on a magnetic rack, washed sequentially with low-salt, high-salt, LiCl, and TE buffers, and DNA–protein complexes were eluted at 65 °C for 30 min in 1× ChIP Elution Buffer. Cross-links were reversed by incubating with 5 M NaCl and Proteinase K for 2 h at 65 °C. DNA was purified with spin columns (DNA purification kit) and quantified by qPCR or electrophoresis as mentioned above using primers flanking putative binding sites in the SPECC1 promoter. Primer sequences are listed in Supplementary Table 1.

### Intracranial orthotopic xenograft model

BALB/c nude mice (4–6 weeks old, *n* = 5/group) were anesthetized with 1.5% isoflurane and immobilized in a stereotactic frame. GBM cells with indicated treatments (5 × 10⁵ cells suspended in 5 μL PBS) were injected intracranially at coordinates: 2 mm right lateral, 0.5 mm anterior to bregma, 3 mm depth. For therapeutic assessment, mice received two cycles of TMZ (50 mg kg⁻¹, intraperitoneal injection (i.p.), 5 days on/2 days off) beginning on day 7 post-injection. Mice were euthanized upon reaching endpoint criteria (weight loss >20%, neurological deficits). Brains were fixed in 4% paraformaldehyde, paraffin-embedded, and sectioned for H&E staining and immunohistochemistry of Ki-67, cleaved Caspase-3, γH2AX, and PGK1. All animal procedures were approved by the Institutional Animal Care and Use Committee (IACUC) of Nanfang Hospital (NFYY-2020-1038) and conducted in accordance with ARRIVE guidelines.

### Statistical analysis

Statistical analysis was performed using GraphPad Prism 9.0. Data are presented as mean ± standard deviation (SD) or mean ± standard error of the mean (SEM) from at least three independent experiments, each performed in triplicate unless stated otherwise. Data normality was assessed via Shapiro-Wilk test (α = 0.05) and homogeneity of variance via F-test. Comparisons between two groups were analyzed using two-tailed unpaired Student’s t-tests or Mann-Whitney U test; multiple groups were compared by one-way ANOVA or two-way ANOVA followed by Tukey post-hoc tests. Survival data were analyzed by Kaplan–Meier curves with log-rank test. Statistical significance was set at *p* < 0.05. The sample sizes, statistical tests and significances have been illustrated in the corresponding graphs or the figure legends.

## Results

### CircSPECC1 is highly expressed in glioma and correlates with poor prognosis

**Fig. 1:**
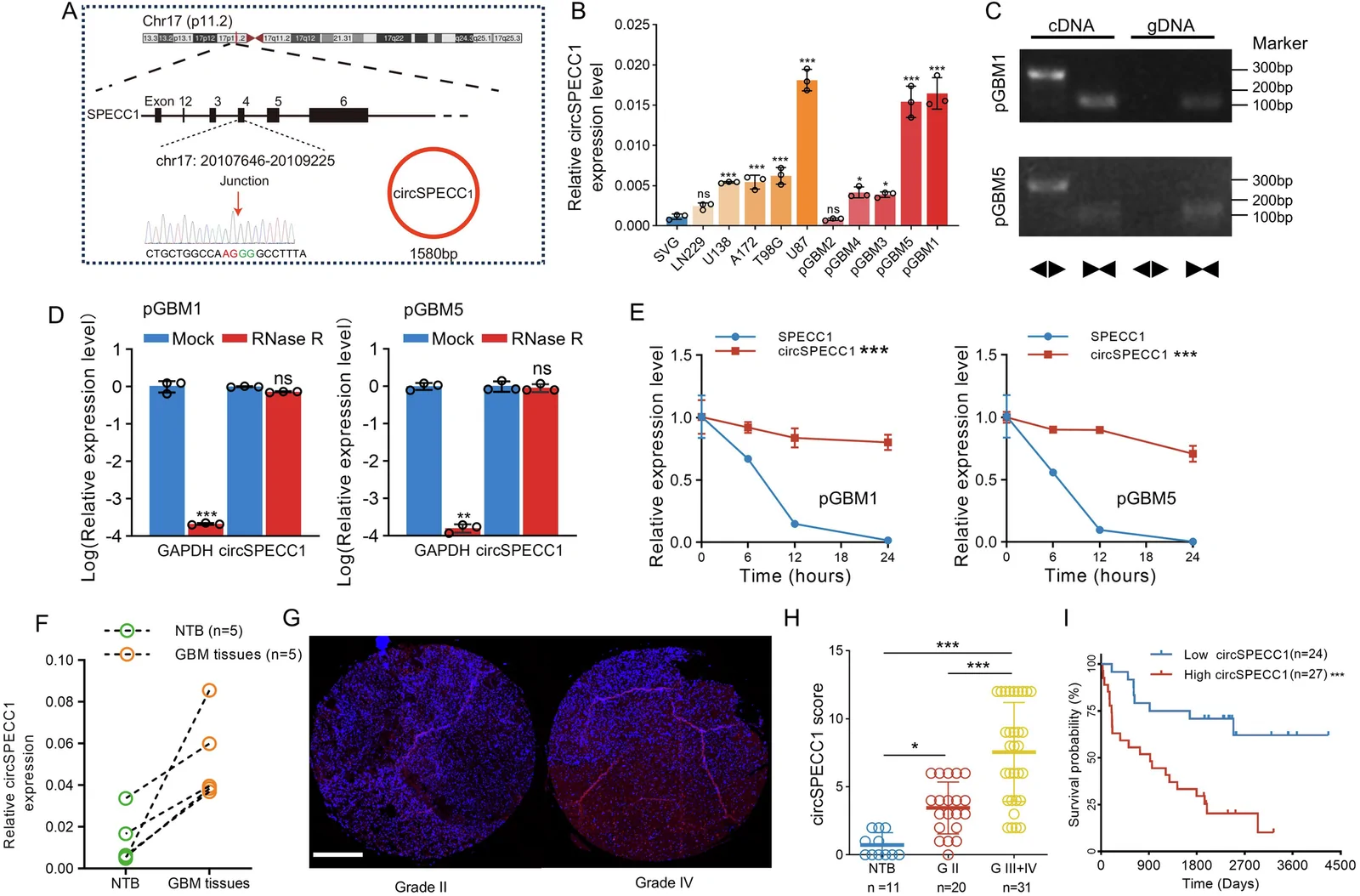
CircSPECC1 is highly expressed in glioma and associated with poor prognosis. **A** Schematic diagram of the genomic location of circSPECC1 derived from its parent gene SPECC1. **B** Relative expression levels of circSPECC1 normalized to ACTB in non-tumor glial cell lines (SVG) and glioma cell lines were examined by qRT-PCR (*N* = 3). **C** Agarose gel electrophoresis detected the circSPECC1 using divergent and convergent primers on cDNA or gDNA of pGBM1 cells. **D** RNA stability of GAPDH and circSPECC1 was measured in pGBM1 and pGBM5 cells treated with RNase R, followed by qRT-PCR (*N* = 3). **E** Half-lives of circSPECC1 and linear SPECC1 were measured in pGBM1 and pGBM5 cells treated with actinomycin D (5 µg/mL) by qRT-PCR (*N* = 3). **F** Relative expression levels of circSPECC1 normalized to ACTB in GBM tissues and their paired non-tumor brain (NTB) tissues were examined by qRT-PCR (*N* = 5). **G** Expression of circSPECC1 in NTB and glioma tissue microarrays was measured by RNA fluorescence in situ hybridization. Scale bar, 500 µm. **H** Staining for circSPECC1 was scored on a scale of 0–12, and the expression scores for circSPECC1 in grades III + IV were compared with those for NTB and grades II (*N* = 11 NTB, *N* = 20 grade II, and *N* = 31 grade III + IV). **I** Kaplan–Meier analysis of overall survival in patients with glioma stratified by high or low circSPECC1 expression (Log-rank test). Data are presented as mean ± SD. Statistical significance was determined by two-tailed Student’s *t*-test (**D**,**F**) and one-way ANOVA with Tukey’s post hoc test (**B**,**E**,**H**). NS, not significant, * *p* < 0.05, ** *p* < 0.01, *** *p* < 0.001.

The circSPECC1 is a 1580 bp circular sequence derived from the parent gene SPECC1 on chromosome 17 (Fig. 1A). The expression of circSPECC1 was then detected in non-tumor glial and glioma cell lines, including both commercially available and primary patient-derived cell lines. While expression varied among different glioma cell lines, higher circSPECC1 expression was observed in most glioma cell lines than in non-tumor glial cell lines (Fig. 1B). Aligned with its circular nature, circSPECC1 was successfully detected in pGBM1 cells using divergent primers from cDNA but not from gDNA (Fig. 1C). Furthermore, treatment with RNase R, which degrades linear RNAs but spares circRNAs, showed that circSPECC1 was resistant to digestion in both pGBM1 and pGBM5 cells, whereas its linear counterpart was degraded, further validating the circular form of SPECC1 (Fig. 1D).

We then assessed the stability of circSPECC1 compared to that of its linear transcript by treating pGBM1 and pGBM5 cells with the transcription inhibitor actinomycin D. The half-life of circSPECC1 was significantly longer than that of its linear transcript, indicating greater stability of the circular RNA in glioma cells (Fig. 1E).

Quantitative PCR revealed that circSPECC1 expression was significantly upregulated in GBM tissues compared to that in paired non-tumor brain tissues (NTB), suggesting a potential role in tumor progression (Fig. 1F). To further elucidate the relationship between circSPECC1 expression and glioma prognosis, fluorescence in situ hybridization analysis was conducted on tissue microarrays containing 51 glioma and 11 NTB, demonstrating that circSPECC1 was primarily localized in the cytoplasm of pGBM1 cells (Fig. 1G), and its expression was markedly higher in glioma tissues than in NTB tissues (Fig. 1H; Supplementary Table 2).

Finally, survival analysis showed that glioma patients with high circSPECC1 expression had significantly worse overall survival than those with low circSPECC1 expression, indicating that circSPECC1 may serve as a prognostic biomarker for glioma (Fig. 1I).

### Hypoxia-inducible factor 1-alpha (HIF-1α) directly activates the transcription of circSPECC1

**Fig. 2:**
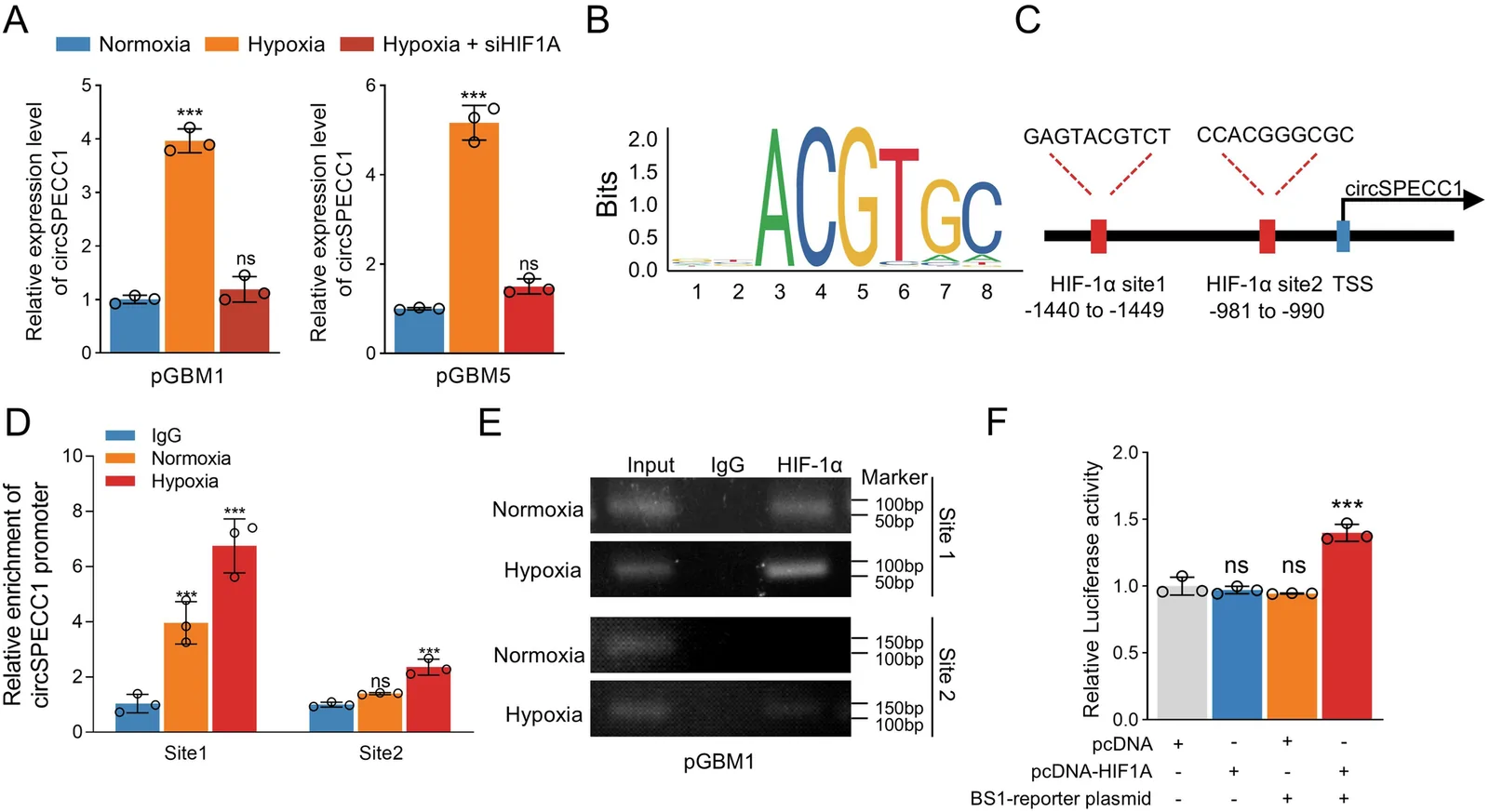
HIF-1α directly activates the transcription of circSPECC1. **A** The expression of circSPECC1 was measured in cells treated with hypoxia, with or without HIF1A knockdown by qRT-PCR (*N* = 3). **B** Predicted HIF-1α binding motifs in the SPECC1 promoter region from the JASPAR database. **C** Schematic representation of the predicted HIF-1α binding sites in the circSPECC1 promoter region. **D**,**E** Chromatin immunoprecipitation followed by qPCR was used to assess HIF-1α binding to the predicted sites in pGBM1 cells under normoxic or hypoxic conditions (*N* = 3). F Luciferase reporter assays were conducted in HEK293T cells overexpressing HIF-1α with or without wild-type SPECC1 promoter constructs (*N* = 3). Data are presented as mean ± SD. Statistical significance was determined by one-way ANOVA with Tukey’s post hoc test (**A**,**D**,**F**). NS, not significant, *** *p* < 0.001.

We moved on to investigate the role of HIF-1α in the transcriptional regulation of circSPECC1 under hypoxic conditions. qRT-PCR analysis confirmed that circSPECC1 expression was significantly upregulated by hypoxia, whereas HIF1A knockdown abrogated this upregulation (Fig. 2A). Bioinformatic analysis using the JASPAR database predicted multiple HIF-1α binding motifs in the promoter region of the SPECC1 gene (Fig. 2B). These potential binding sites are schematically illustrated in the circSPECC1 promoter (Fig. 2C).

To validate the direct interaction of HIF-1α with the SPECC1 promoter, ChIP-qPCR assays were performed. The results demonstrated that HIF-1α binds to the predicted site1 under both normoxic and hypoxic conditions but could not bind to the predicted site 2 under normoxia in pGBM1 cells (Fig. 2D, E).

Furthermore, luciferase reporter assays were performed to assess the transcriptional activity of the SPECC1 promoter in response to HIF-1α overexpression. The results revealed that wild-type promoter constructs exhibited significant luciferase activity upon HIF-1α overexpression (Fig. 2F). These findings suggest that HIF-1α directly activates circSPECC1 transcription by binding to specific motifs in the SPECC1 promoter.

### CircSPECC1 depletion diminishes the malignant phenotype of GBM cells in vitro

**Fig. 3:**
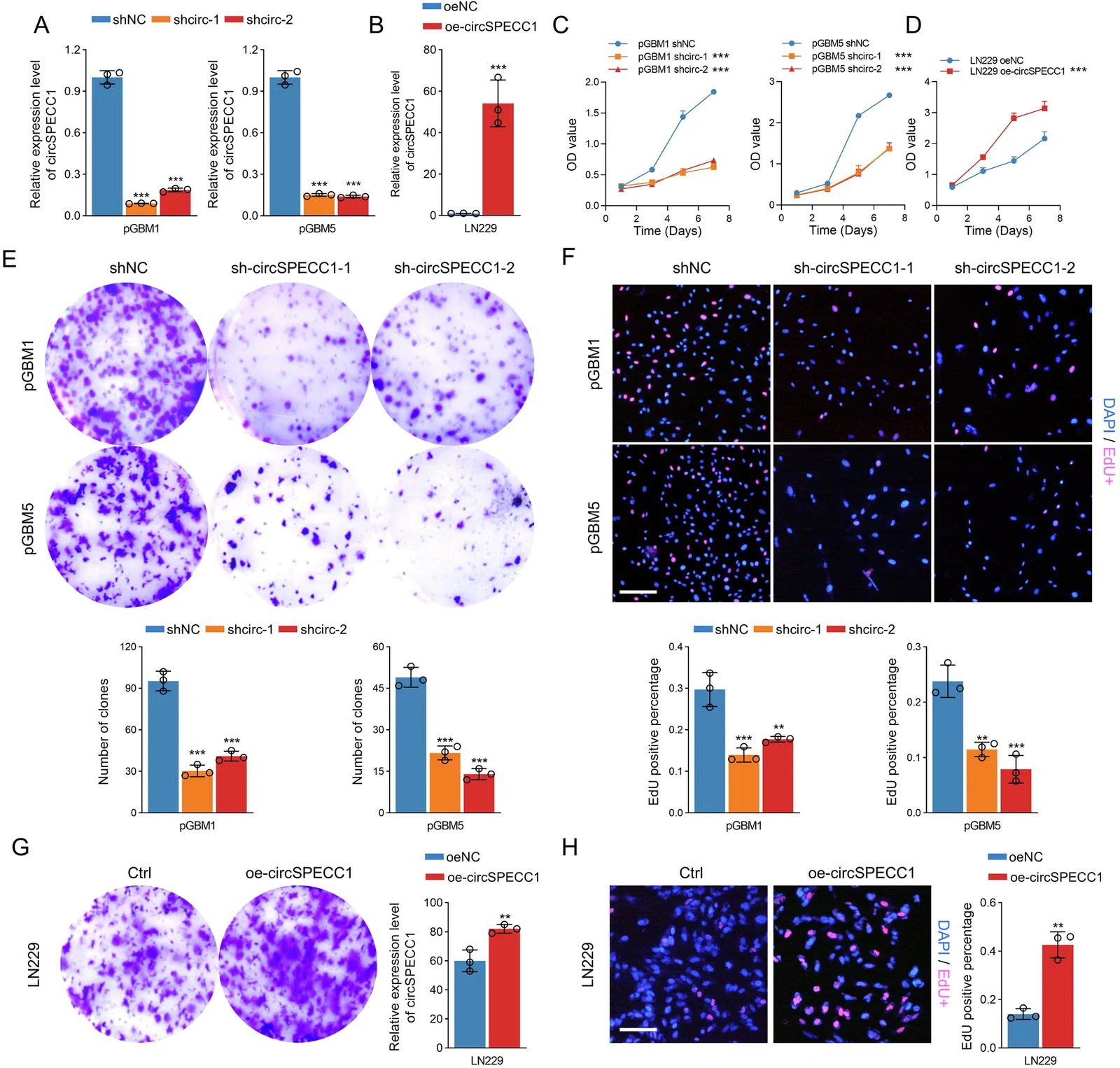
CircSPECC1 depletion diminishes GBM cell proliferation. **A** The knockdown efficiency of circSPECC1 was validated by qRT-PCR in pGBM1 and pGBM5 cells (*N* = 3). **B** The efficiency of circSPECC1 overexpression was confirmed by qRT-PCR in LN229 cells (*N* = 3). **C**,**D** Cell viability of circSPECC1-depleted or overexpressed GBM cells was assessed using CCK-8 assays (*N* = 3). **E** Colony formation assays demonstrated reduced cell viability in circSPECC1-depleted pGBM1 and pGBM5 cells (*N* = 3). **F** EdU assays further confirmed decreased proliferation in circSPECC1-depleted pGBM1 and pGBM5 cells (*N* = 3). Scale bar, 100 µm. **G** Colony formation assays showed increased viability in circSPECC1-overexpressing LN229 cells (*N* = 3). **H** Enhanced proliferation in circSPECC1-overexpressing LN229 cells was also confirmed by EdU assays (*N* = 3). Scale bar, 100 µm. Data are presented as mean ± SD. Statistical significance was determined by two-tailed Student’s t-test (**B**,**G**,**H**) and one-way ANOVA with Tukey’s post hoc test (**A**,**C–F**). ** *p* < 0.01, *** *p* < 0.001.

The role of circSPECC1 in the proliferation of GBM cells was investigated using a combination of knockdown and overexpression methods, which provided a comprehensive understanding of its functional significance. circSPECC1 is highly expressed in the patient-derived GBM lines pGBM1 and pGBM5 but exhibits relatively low expression in LN229 (Fig. 1B). Accordingly, loss-of-function experiments were conducted in pGBM1 and pGBM5, whereas gain-of-function experiments were performed in LN229 cells. The efficiency of circSPECC1 knockdown in the pGBM1 and pGBM5 cell lines was confirmed by qRT-PCR analysis, which provided clear evidence of successful gene silencing, whereas the overexpression of circSPECC1 in LN229 cells was validated (Fig. 3A, B). To assess the effect of circSPECC1 modulation on cell viability, CCK-8 assays were performed. The results indicated that depletion of circSPECC1 led to a marked reduction in cell viability, demonstrating its critical role in sustaining cell growth (Fig. 3C), whereas overexpression of circSPECC1 resulted in a significant increase in cell viability (Fig. 3D).

Further validation of these findings was achieved using colony formation assays, which revealed that the depletion of circSPECC1 significantly impaired the viability of both pGBM1 and pGBM5 cells (Fig. 3E). EdU assays confirmed decreased proliferation rates in these cell lines (Fig. 3F). In contrast, the overexpression of circSPECC1 in LN229 cells not only enhanced their viability but was also corroborated by both colony formation and EdU assays, which illustrated a robust increase in proliferation (Fig. 3G–H). Collectively, these findings strongly suggested that circSPECC1 plays a pivotal role in promoting the proliferation of GBM cells, thereby underscoring its potential significance in GBM progression and its implications for future therapeutic strategies.

**Fig. 4:**
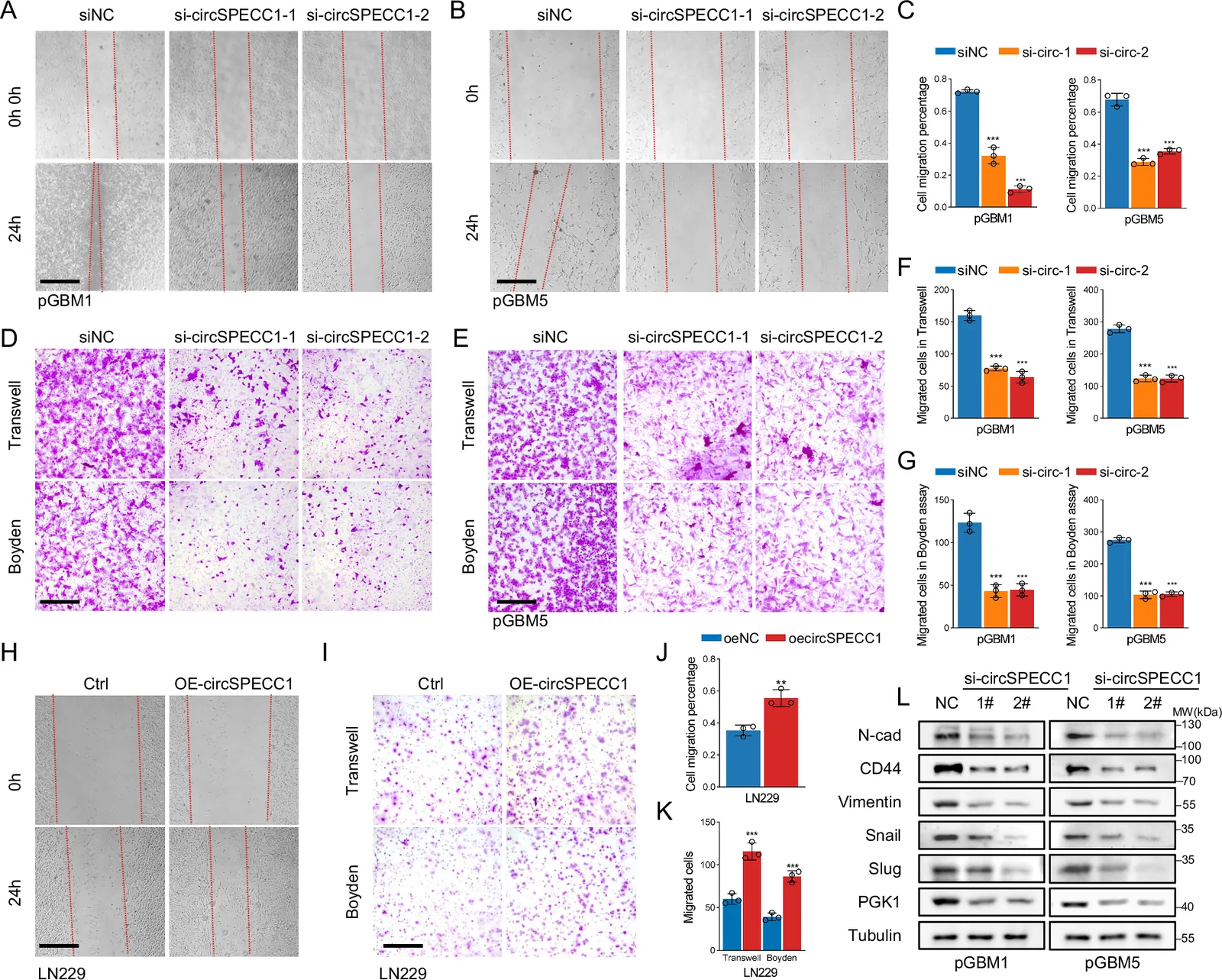
CircSPECC1 depletion diminishes GBM migration and invasion. **A**,**B** Wound-healing assays were used to evaluate the migration ability of circSPECC1-depleted pGBM1 (**A**) and pGBM5 cells (**B**). **C** Quantitative analysis of the wound-healing assays. Scale bar, 200 µm. **D**,**E** Transwell and Boyden assays were conducted to assess the migration and invasion abilities of circSPECC1-depleted pGBM1 (**D**) and pGBM5 cells (**E**), respectively (*N* = 3). **F**,**G** Quantitative analysis of the Transwell (**F**) and Boyden (**G**) assays. Scale bar, 200 µm. **H**,**J** Wound-healing assays were performed to determine the migration ability of circSPECC1-overexpressing LN229 cells (*N* = 3). Scale bar, 200 µm. **I**,**K** Transwell and Boyden assays were used to assess the migration and invasion abilities of circSPECC1-overexpressing LN229 cells (*N* = 3). Scale bar, 200 µm. **L** Western blot was used to examine the expression of mesenchymal markers and PGK1 in circSPECC1-depleted pGBM1 and pGBM5 cells, respectively. Data are presented as mean ± SD. Statistical significance was determined by two-tailed Student’s t-test (**J**,**K**) and one-way ANOVA with Tukey’s post hoc test (**C**,**F**,**G**). ** *p* < 0.01, *** *p* < 0.001.

The effect of circSPECC1 on GBM cell migration and invasion was also evaluated. The migration ability of circSPECC1-depleted pGBM1 and pGBM5 cells was significantly reduced, as indicated by the wound-healing assays (Fig. 4A–C). Similarly, Transwell and Boyden assays showed that circSPECC1 knockdown reduced both the migration and invasion abilities of these cells (Fig. 4D–G). Conversely, circSPECC1 overexpression in LN229 cells led to enhanced migration, as demonstrated by wound-healing assays (Fig. 4H–K). Furthermore, circSPECC1 knockdown in pGBM1 and pGBM5 cells led to a significant reduction in the expression of mesenchymal markers, including N-cadherin, CD44, Vimentin, Snail, and Slug (Fig. 4L).

These results indicated that circSPECC1 is involved in promoting GBM cell proliferation, migration, and invasion, suggesting its critical involvement in tumor progression.

### circSPECC1 physically interacts with IGF2BP2

Although the role of circRNAs as miRNA sponges is well-established, their interactions with RNA-binding proteins (RBPs) have been less studied and represent a promising frontier in glioma research. Understanding these interactions could provide deeper insights into the molecular mechanisms underlying glioma progression and offer novel avenues for therapeutic interventions. Therefore, potential RBPs that interact with circSPECC1 were investigated.

**Fig. 5:**
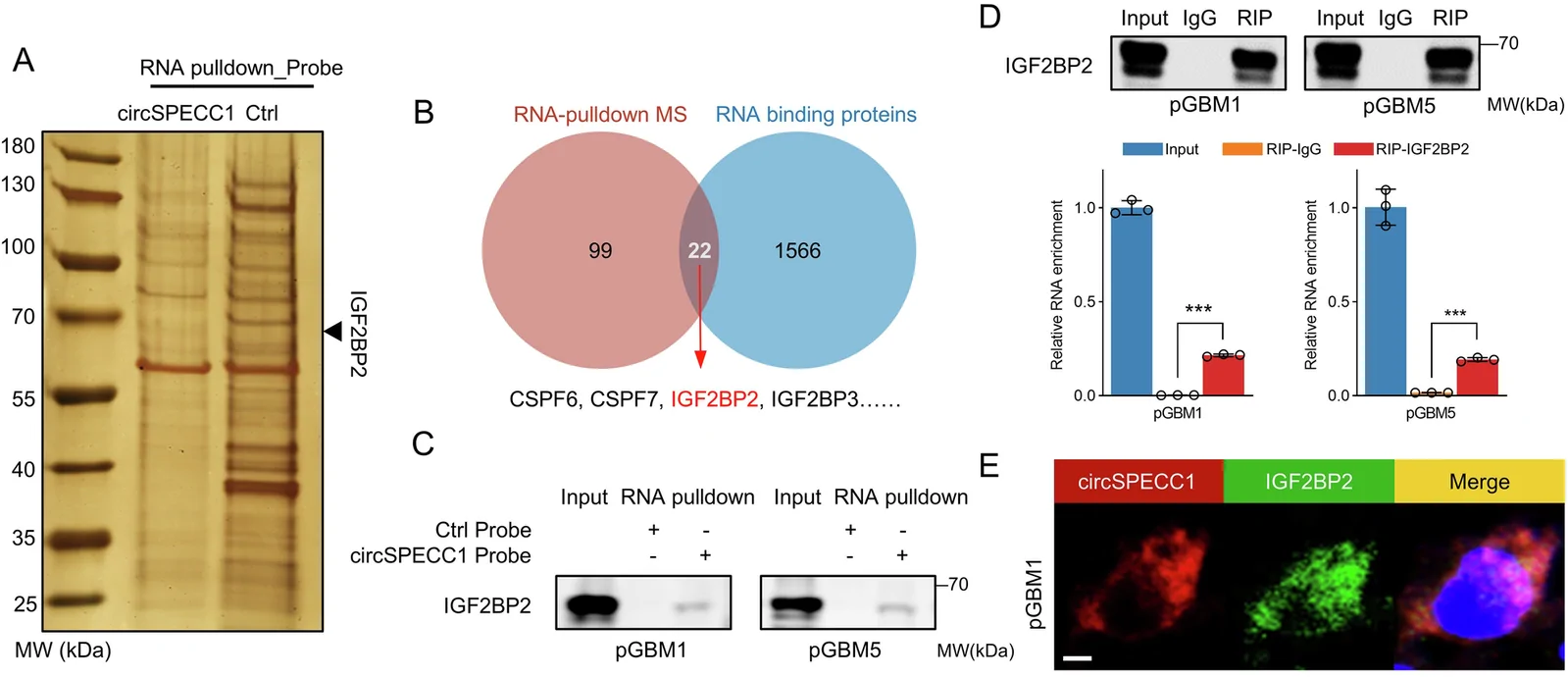
CircSPECC1 interacts with RNA-binding protein IGF2BP2. **A** Silver-stained gel of proteins pulled down by a biotinylated circSPECC1 probe versus an antisense control probe. **B** Venn diagram of RNA-binding proteins identified by mass spectrometry after circSPECC1 pulldown. **C** Western blot validation of IGF2BP2 enrichment in the circSPECC1 pulldown complex. **D** RNA immunoprecipitation assays using an anti-IGF2BP2 or anti-IgG antibody confirming the association with circSPECC1 (*N* = 3). IgG served as a control. **E** Immunofluorescence showing co-localization of circSPECC1 (red) and IGF2BP2 (green) in the cytoplasm of pGBM1 cells (*N* = 3). Nuclei were stained with DAPI (blue). Scale bar, 10 µm. Data are presented as mean ± SD. Statistical significance was determined by one-way ANOVA with Tukey’s post hoc test (**D**). *** *p* < 0.001.

An RNA pulldown assay using biotin-tagged circSPECC1 was performed to identify potential circSPECC1-interacting proteins (Fig. 5A). Mass spectrometry analysis of circSPECC1-bound proteins identified multiple candidates, including members of the IGF2BP family, IGF2BP2 and IGF2BP3 (Fig. 5B; Supplementary Table 3), which are highly expressed and involved in oncogenic processes in glioma [10, 19–21]. Although the role of IGF2BP3 in mediating an immunosuppressive phenotype via interaction with circNEIL3 has recently been established [10], the functional significance of IGF2BP2-circRNA interactions in gliomas remains largely unexplored, indicating a significant knowledge gap. To confirm this physical binding, we performed an RNA pulldown assay using a biotin-labeled circSPECC1 probe followed by western blot analysis, which confirmed that IGF2BP2 is specifically enriched in the circSPECC1 pulldown complex (Fig. 5C). This interaction was further validated by RIP assays using an anti-IGF2BP2 antibody, which showed significant enrichment of circSPECC1 compared to the IgG control (Fig. 5D). These data provide direct evidence that circSPECC1 physically interacts with IGF2BP2. Finally, immunofluorescence analysis with circSPECC1 probes and IGF2BP2 antibodies indicated the co-localization of circSPECC1 and IGF2BP2 within the cells, supporting a functional interaction in stabilizing downstream targets (Fig. 5E). Taken together, these results suggest that circSPECC1 recruits IGF2BP2 in GBM, although its precise downstream activity remains elusive.

### CircSPECC1/IGF2BP2 complex stabilizes PGK1 mRNA

**Fig. 6:**
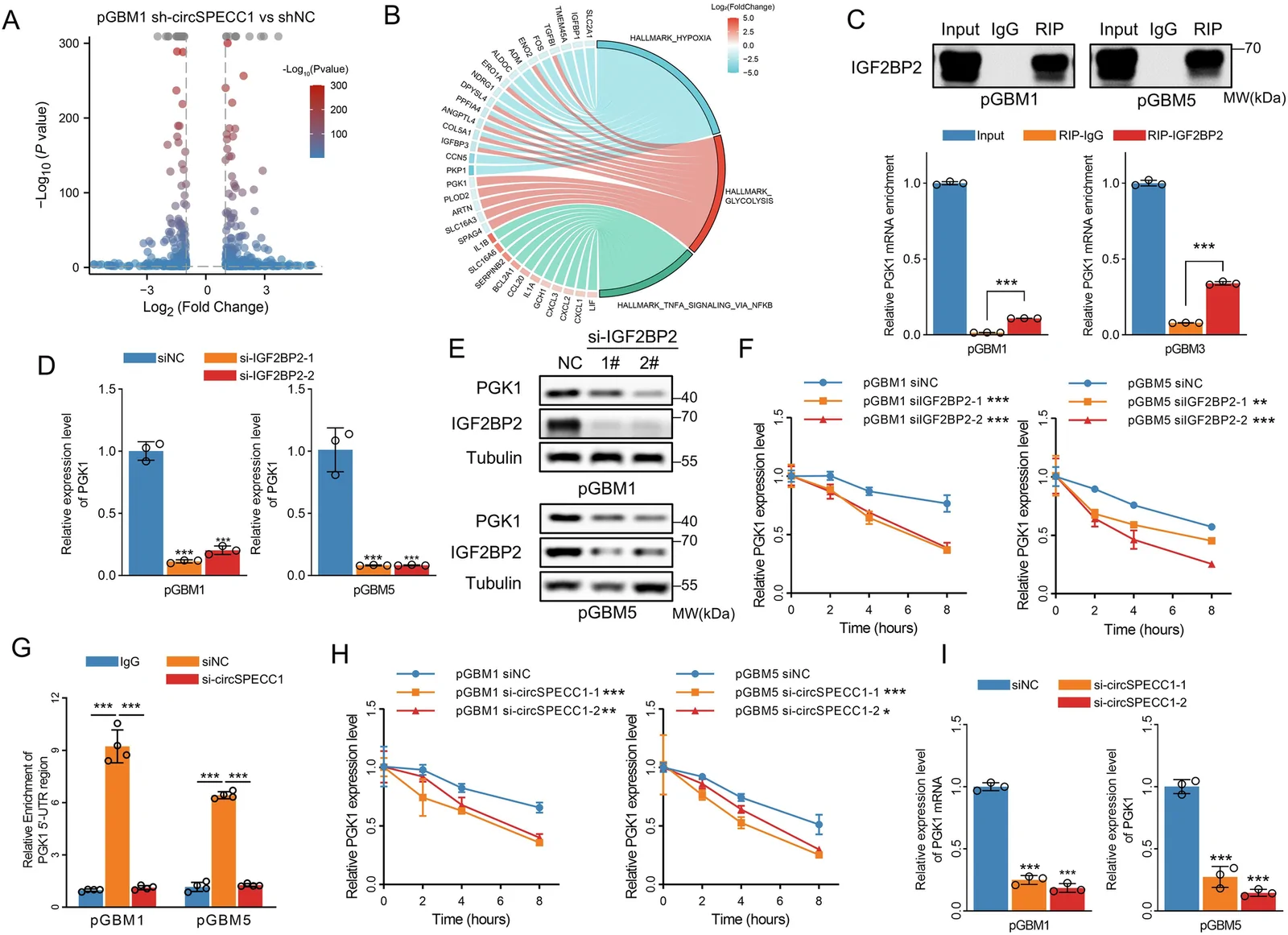
Interaction between circSPECC1 and IGF2BP2 enhances the stability of PGK1. **A** Volcano plot depicting differentially expressed genes in pGBM1 cells with circSPECC1 knockdown. **B** Gene set enrichment analysis reveals significant enrichment of glycolysis and hypoxia pathways among genes downregulated upon circSPECC1 knockdown. **C** RIP-qPCR assay showing enrichment of PGK1 mRNA in anti-IGF2BP2 immunoprecipitants compared to IgG (*N* = 3). **D** qRT-PCR analysis of PGK1 mRNA expression after IGF2BP2 knockdown in pGBM1 and pGBM5 cells (*N* = 3). **E** Western blot analysis of PGK1 protein levels after IGF2BP2 knockdown in pGBM1 and pGBM5 cells. **F** PGK1 mRNA stability assay after IGF2BP2 knockdown in pGBM1 and pGBM5 cells (*N* = 3). G RIP-qPCR assay assessed the effect of circSPECC1 knockdown on the interaction between IGF2BP2 and PGK1 mRNA (*N* = 4). **H** PGK1 mRNA stability assay after circSPECC1 knockdown in pGBM1 and pGBM5 cells (*N* = 3). **I** qRT-PCR analysis of PGK1 mRNA expression after circSPECC1 knockdown in pGBM1 and pGBM5 cells (*N* = 3). Data are presented as mean ± SD. Statistical significance was determined by one-way ANOVA with Tukey’s post hoc test (**C**,**D**,**F–I**). * *p* < 0.05, ** *p* < 0.01, *** *p* < 0.001.

To investigate the impact of circSPECC1 on gene expression in GBM cells, we first performed a transcriptomic analysis of pGBM1 cells with circSPECC1 knockdown (Fig. 6A; Supplementary Table 4). GO and KEGG enrichment analysis were performed, and the results suggested that knockdown of circSPECC1 led to significant downregulation of hypoxia and glycolysis phenotype (Fig. 6B; Supplementary Table 5).

Among the downregulated markers, a paradoxical role of PGK1 in GBM has been previously reported [22]. To establish that IGF2BP2 was directly associated with PGK1 transcripts, we performed RIP-qPCR using anti-IGF2BP2 antibodies. PGK1 mRNA was significantly enriched in IGF2BP2 immunoprecipitates compared to IgG controls (Fig. 6C), indicating that PGK1 is a bona fide target of IGF2BP2 in our GBM models. Quantitative PCR and Western blot analyses showed that IGF2BP2 knockdown led to a significant decrease in PGK1 mRNA and protein levels, respectively (Fig. 6D, E). To directly assess the effects of IGF2BP2 on PGK1 mRNA stability, we performed Actinomycin D chase experiments. Upon IGF2BP2 knockdown, PGK1 mRNA decay was significantly accelerated, resulting in a reduced half-life (Fig. 6F). This indicated that IGF2BP2 exerts a stabilizing effect on PGK1 transcripts.

We also investigated the role of circSPECC1 in this regulatory axis. RIP assays confirmed that circSPECC1 knockdown decreased the interaction between IGF2BP2 and PGK1 mRNA, highlighting circSPECC1’s role in promoting this interaction (Fig. 6G). The circSPECC1 depletion led to a marked reduction in PGK1 mRNA and protein levels, as confirmed by qRT-PCR and western blot analyses (Fig. 6H–I). These findings support the notion that circSPECC1 is required for the efficient IGF2BP2-mediated stabilization of PGK1 mRNA.

### CircSPECC1/PGK1 increases GBM cell glycolysis rate and confers TMZ resistance

GBM cells exhibit enhanced glycolytic capacity to support bioenergetic needs and the accumulation of lactate and acidic conditions within tumors, which further contribute to the evasion of chemotherapy-induced apoptosis and promote DNA repair mechanisms [23–25]. PGK1 is a key metabolic enzyme involved in glycolysis and has been implicated in the regulation of malignant phenotypes of GBM [26, 27].

**Fig. 7:**
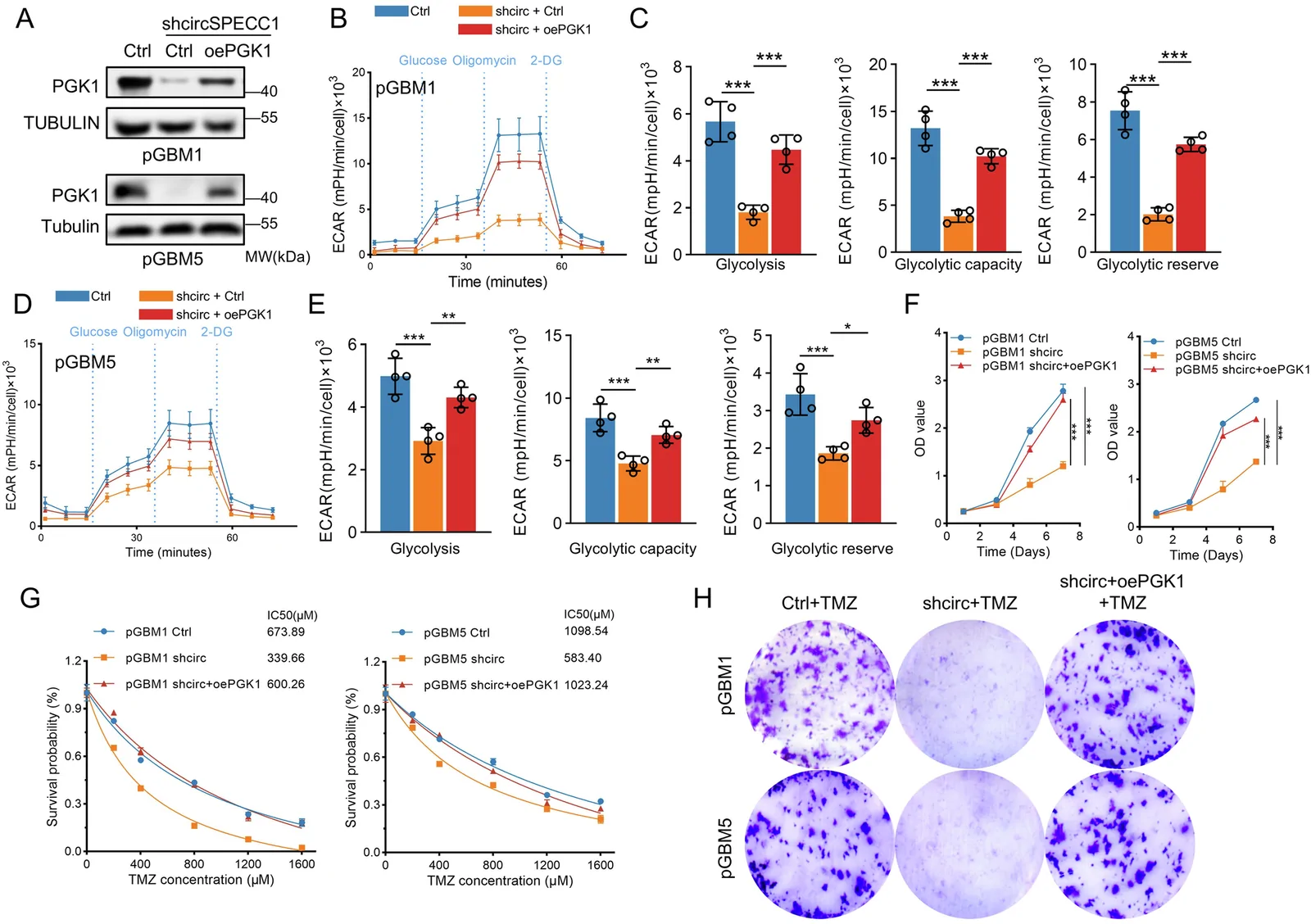
CircSPECC1/PGK1 increases GBM cell glycolysis rate and confers TMZ resistance. **A** Western blot analysis confirmed the knockdown efficiency of shcircSPECC1 and PGK1 overexpression rescue in pGBM1 and pGBM5 cells. **B–E** Seahorse XF Glycolysis Stress Test measuring the extracellular acidification rate (ECAR) in (**B**,**C**) pGBM1 and (**D**,**E**) pGBM5 cells under the indicated conditions (*N* = 4). Glycolysis, glycolytic capacity, and glycolytic reserve were quantified. **F** CCK8-assays were used to analyze cell viability in pGBM1 and pGBM5 cells with indicated treatments (*N* = 3). **G**,**H** Half-maximal inhibitory concentrations (IC_50_) and inhibitory effect of TMZ were assessed in pGBM1 and pGBM5 cells with indicated treatments by CCK8-assay (**G**) and clone formation assay (**H**), respectively (*N* = 3). Data are presented as mean ± SD. Statistical significance was determined by one-way ANOVA with Tukey’s post hoc test (**C**,**E–G**). * *p* < 0.05, ** *p* < 0.01, *** *p* < 0.001.

Based on these findings, we sought to determine whether PGK1 mediates the biological effects of circSPECC1 on GBM. First, we confirmed that the knockdown of circSPECC1 resulted in a significant reduction in PGK1 expression. Furthermore, transfection with a PGK1 overexpression plasmid successfully restored PGK1 levels (Fig. 7A). To evaluate the effect of circSPECC1 knockdown on the glycolytic profile of GBM cells, we performed the Seahorse XF glycolysis stress assay. The extracellular acidification rate was measured to quantify key glycolytic flux parameters, including basal glycolysis, glycolytic capacity, and glycolytic reserve. In both pGBM1 and pGBM5 cells, the knockdown of circSPECC1 led to a marked decrease in the glycolysis rate, glycolytic capacity, and glycolytic reserve (Fig. 7B–E). However, PGK1 overexpression in these cells rescued the inhibited glycolytic capacity, indicating that PGK1 plays a critical role in modulating glycolytic metabolism during circSPECC1 knockdown (Fig. 7B–E).

We also evaluated the effects of circSPECC1 knockdown and PGK1 overexpression on cell viability and proliferation. The circSPECC1 knockdown significantly reduced the viability and proliferation of pGBM1 cells, as shown by the CCK-8 assay (Fig. 7F). In contrast, PGK1 overexpression rescued cell viability and proliferation, underscoring the pivotal role of PGK1 in sustaining GBM cell growth under circSPECC1 knockdown conditions (Fig. 7F). Finally, we examined the sensitivity of GBM cells to TMZ after circSPECC1 knockdown and PGK1 overexpression. While circSPECC1 knockdown significantly sensitized pGBM1 and pGBM5 cells to TMZ (IC_50_ [μM]: Ctrl vs shcircSPECC1, 673.89 vs 339.66 and 1098.54 vs 583.40 in pGBM1 and pGBM5, respectively), overexpression of PGK1 antagonized this effect (IC_50_ [μM]: shcircSPECC1+oePGK1 group, 600.26 and 1023.24 in pGBM1 and pGBM5, respectively), suggesting that PGK1 mediates the effect of circSPECC1 in regulating TMZ resistance in GBM cells (Fig. 7G–H).

Taken together, these results indicate that circSPECC1 regulates glycolysis and TMZ sensitivity in GBM cells by modulating PGK1 expression, highlighting a potential therapeutic target for overcoming metabolic adaptations and drug resistance in GBM.

### CircSPECC1 depletion overcomes TMZ resistance in GBM xenograft

**Fig. 8:**
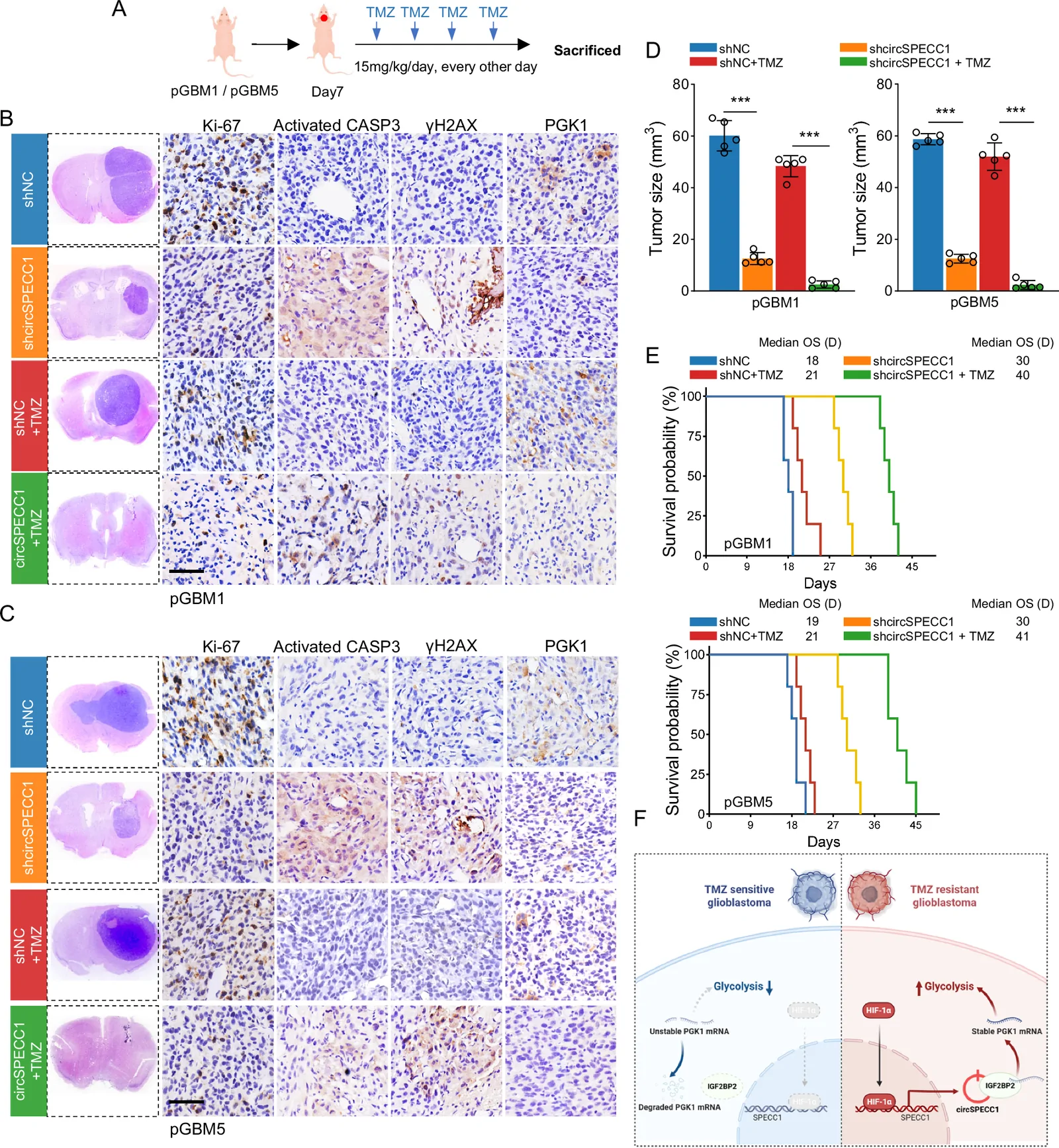
CircSPECC1 depletion overcomes TMZ resistance in GBM xenograft. **A** Experimental timeline for the orthotopic xenograft model and TMZ treatment regimen (*N* = 5 per group). **B**,**C** Representative H&E staining and immunohistochemistry (IHC) for Ki-67, cleaved caspase-3, γH2AX, and PGK1 in pGBM1-(**B**) and pGBM5-derived tumors (**C**), respectively. Scale bars, 50 μm. D Tumor volumes at endpoint in pGBM1 and pGBM5 xenograft models. **E** Kaplan–Meier survival curves with log-rank test for pGBM1 and pGBM5 xenograft models. **F** Graphical abstract illustrating the main findings of the study. Data are presented as mean ± SD. Statistical significance was determined by one-way ANOVA with Tukey’s post hoc test (**D**). *** *p* < 0.001.

To evaluate the therapeutic potential of circSPECC1 in GBM, we established an orthotopic xenograft model using TMZ-resistant pGBM1 and pGBM5 cells (Fig. 8A). The circSPECC1 knockdown alone significantly suppressed tumor growth (pGBM1: shcircSPECC1 12.58 ± 2.29 mm³ vs. control 60.09 ± 5.87 mm³, *p* < 0.001; pGBM5: shcircSPECC1 10.58 ± 12.48 mm³ vs. control 58.69 ± 2.15 mm³, *p* < 0.001) and extended the median survival time (pGBM1: shcircSPECC1 30 vs. control 18 days, *p* < 0.001; pGBM5: shcircSPECC1 30 vs. control 19 days, *p* < 0.001) (Fig. 8B–E). Accordingly, circSPECC1 knockdown resulted in reduced expression of Ki-67 and PGK1, and increased expression of cleaved caspase-3. However, TMZ monotherapy showed no efficacy in either model, with no significant changes in γH2AX expression, which was used as a robust marker of DNA damage and quantified by immunohistochemical staining (Fig. 8B–E).

Critically, combining circSPECC1 knockdown with TMZ resulted in synergistic tumor regression compared with that in the TMZ monotherapy group (pGBM1: shcircSPECC1+TMZ 2.54 ± 1.44 mm³ vs. TMZ 48.30 ± 4.07 mm³, *p* < 0.001; pGBM5: shcircSPECC1+TMZ 2.30 ± 1.71 mm³ vs. TMZ 12.48 ± 1.66 mm³, *p* < 0.001; Fig. 8D) and extended the median survival to 40 and 41 days (Fig. 8E). Accordingly, increased γH2AX expression was observed in the combination group compared with the other groups (Fig. 8B, C).

## Discussion

In this study, we focused on the circular RNA circSPECC1, which is significantly upregulated in GBM tissues and correlates with poor patient prognosis. Our findings demonstrate that hypoxia induces circSPECC1 expression, and silencing circSPECC1 significantly hinders the proliferation, migration, and invasion of GBM cells, implicating its critical role in promoting tumor malignancy. Additionally, we elucidated the underlying molecular mechanisms, revealing that circSPECC1 interacted with the RBP IGF2BP2 and PGK1 mRNA, thereby influencing glycolysis, which is crucial for GBM cell survival and proliferation (Fig. 8F). These insights establish a novel mechanism for the intersection between circRNA-RBP-mRNA scaffolding and glycolytic flux and underscore the potential of circSPECC1 as a therapeutic target to improve treatment outcomes for patients with GBM. This study provides significant insights into the molecular mechanisms underlying the role of circSPECC1 in GBM.

The circSPECC1 has emerged as a pivotal regulator of diverse cancers, exhibiting pleiotropic functions that are highly dependent on cellular and microenvironmental contexts. In certain cancers, circSPECC1 acts as a potent oncogenic driver by promoting the epithelial-mesenchymal transition. For instance, in prostate cancer, its overexpression enhances epithelial-mesenchymal transition by upregulating vascular endothelial growth factor, matrix metalloproteinase 2/9, Vimentin, and N-cadherin, and concurrently downregulating E-cadherin [28]. In contrast, circSPECC1 functions as a tumor suppressor in gastric cancer. It interacts with autophagy-related 4 B cysteine peptidase to promote its ubiquitination, subsequently suppressing anoikis resistance and limiting metastasis [29].

An additional layer of complexity arises from the reported protein-coding capacity of circSPECC1, which generates a 415–amino acid peptide, SPECC1-415aa. Cheng et al*.* emphasized the translational output of circSPECC1, suggesting that its oncogenic activity may, at least in part, be mediated by this peptide in recurrent GBM [17]. In contrast, our previous findings established a pro-tumorigenic role for circSPECC1 under hypoxic conditions [15], and Lan et al. reported that it facilitates glioma tumorigenesis via the miR-615-5p/huntingtin-interacting protein-1/AKT axis [16]. In this study, we identified a novel circRNA-RBP scaffolding mechanism of circSPECC1 under hypoxic conditions. We confirmed that it is directly transcriptionally regulated by HIF-1α and promotes glycolytic flux by facilitating the interaction between IGF2BP2 and PGK1 mRNA, ultimately conferring enhanced TMZ resistance in GBM cells. Hypoxia and m6A modifications have recently been recognized as pivotal factors that determine the fate of circRNAs. Emerging evidence suggests that the biological fate of m6A-modified RNAs is dictated by the competitive and sometimes antagonistic actions of different m6A readers [30, 31]. The YTH domain family proteins (including YTHDF1/2/3 and YTHDC1) are generally associated with translational promotion or RNA decay [32, 33], whereas the IGF2BP family (IGF2BP1/2/3) preferentially stabilizes target transcripts and enhances their persistence [34, 35]. Our findings provide a plausible explanation for the discrepancy in the role of circSPECC1 in GBM and underscore the importance of microenvironmental cues in dictating the fate and function of circRNAs. Future studies dissecting the precise m6A landscape of circSPECC1 and reader occupancy under different microenvironmental conditions will be critical to fully elucidate this regulatory axis.

Most studies have focused on the well-established function of circSPECC1 as an miRNA sponge, a canonical mechanism by which circRNAs sequester miRNAs to derepress target mRNA expression. In acute lymphoblastic leukemia, circSPECC1 drives proliferation, glycolysis, and ferroptosis resistance by sponging miR-494-3p to upregulate neuroepithelial cell transforming 1 [36] and miR-193b-3p to activate neurogenic locus notch homolog protein 1 [37]. Despite robust literature on the miRNA sponge function of circSPECC1, few studies have explored its interaction with RBPs. One notable example is RBP ELAVL1, which recognizes m6A modification of circSPECC1, suppresses its expression, and stabilizes autophagy-related 4 B cysteine peptidase to promote gastric cancer metastasis [29]. Another m6A reader, IGF2BP1, binds to circSPECC1, enhancing its stability and enabling translation of the tumor-suppressive protein SPECC1-415aa [17]. In this study, we identified a novel interaction between circSPECC1 and IGF2BP2, which further facilitated the binding of IGF2BP2 to PGK1 mRNA, leading to increased stability of PGK1 mRNA. This interaction enhances glycolysis and contributes to TMZ resistance in GBM. Notably, several PGK1-targeted miRNAs have been reported to be sponged by circRNAs in cancer [38–40], indicating that miRNA sponging may also represent a complementary or secondary layer of regulation and potential context-specific shifts in the regulation mechanism. Further investigation of these RBP-mediated mechanisms will provide a deeper understanding of circSPECC1’s multifaceted functions in cancer biology.

Similar to our study, circSPECC1 promotes glycolysis in ALL [36], indicating its critical role in cancer glycolysis. Glycolytic reprogramming in GBM can either promote or, in rare contexts, attenuate TMZ resistance depending on distinct or even paradoxical mechanisms. Enhanced glycolytic flux supplies energy and anabolic precursors and maintains the redox balance, particularly in stem-like and hypoxic tumor subpopulations. For example, circKIF4A sponges miR-335-5p to derepress aldolase A, a rate-limiting glycolytic enzyme, which then drives glycolysis and attenuates TMZ-induced apoptosis by sustaining nucleotide synthesis and redox homeostasis [41]. Paradoxically, inhibiting glycolysis can sometimes increase TMZ responsiveness owing to increased oxidative stress. Under hypoxia, TP53‑induced glycolysis and apoptosis regulator (TIGAR) shifts metabolism toward the pentose phosphate pathway, reducing glycolytic output. TIGAR silencing increases the glycolytic output and disrupts the NADPH-mediated redox balance, exacerbating ROS accumulation and TMZ-induced apoptosis under hypoxic conditions [42]. Notably, both the primary GBM cell lines were established from MGMT-unmethylated tumors. Although MGMT removes O^6^-methylguanine adducts to directly counteract TMZ-induced DNA damage, the circSPECC1/PGK1 axis appears to function in parallel by sustaining energy production, redox balance, and stress tolerance. Nevertheless, inhibition of the serine synthesis pathway, a branch of glycolysis that produces serine, can enhance TMZ efficacy by decreasing MGMT expression and reducing reactive oxygen species-mediated DNA damage in GBM [43]. This dual-layer model may explain why metabolic targeting sensitizes MGMT-unmethylated GBM cells to TMZ.

PGK1 is a pivotal enzyme in glycolysis that catalyzes the conversion of 1,3-bisphosphoglycerate to 3-phosphoglycerate to generate ATP [44]. In addition to its canonical metabolic role, accumulating evidence indicates that PGK1 functions as a protein kinase under hypoxic stress and acts as a central regulator of autophagy [45]. Therefore, hypoxia-induced stabilization of PGK1 by circSPECC1 is likely to promote TMZ resistance via two synergistic mechanisms. First, enhanced glycolytic flux ensures sustained ATP supply for energy-intensive DNA repair processes and diverts glycolytic intermediates into the pentose phosphate pathway, contributing to redox homeostasis and limiting TMZ-induced reactive oxygen species [46]. Second, under hypoxic conditions, PGK1 is acetylated by ARD1/NAA10 and translocated to interact with Beclin-1, where it phosphorylates BECN1 at Ser30 to activate the VPS34 complex and initiate autophagy [45, 47]. This hypoxia-induced autophagy promotes tumor cell survival by recycling damaged organelles and maintaining nutrient homeostasis, thereby further buffering TMZ-induced cytotoxicity. Collectively, these findings identify circSPECC1-mediated stabilization of PGK1 as a hypoxia-adaptive mechanism that integrates metabolic reprogramming and autophagy to drive TMZ resistance in GBM, while future studies are needed to define its therapeutic tractability and clinical relevance.

The exceptional stability and tumor-enriched expression of circSPECC1 position it as a promising candidate biomarker for GBM. CircRNAs are readily detectable in patient plasma and cerebrospinal fluid and can noninvasively reflect tumor-associated molecular state [48]. In particular, liquid biopsy–based detection of exosomal circRNAs provides a clinically tractable approach for longitudinal disease monitoring, enabling early diagnosis, assessment of treatment response, and surveillance for recurrence, thereby complementing tissue-based qRT-PCR analyses [49]. Therapeutic targeting of circSPECC1 requires the high specificity of its back-splice junction to preserve linear SPECC1 function. Although antisense oligonucleotides (ASOs) and siRNAs represent feasible strategies, their application in GBM remains constrained by limited blood–brain barrier (BBB) penetration and suboptimal tumor delivery [50]. Receptor-mediated transcytosis via the transferrin receptor has emerged as a promising solution, with engineered oligonucleotide delivery platforms achieving efficient BBB crossing and widespread central nervous system penetration in preclinical models [51]. In parallel, microbubble-assisted focused ultrasound can transiently disrupt the BBB to enhance local drug accumulation at tumor sites, although concerns regarding neuroinflammation, hemorrhage risk, and data on repeated BBB opening remain sparse [52]. Together, these advances support circSPECC1 as a compelling biomarker and therapeutic target in GBM, while underscoring the need for continued optimization of delivery strategies and rigorous evaluation of safety and efficacy to enable clinical translation.

This study has some limitations. First, our mechanistic findings were largely derived from GBM cell lines and mouse models, which do not fully recapitulate the cellular, molecular, and microenvironmental complexities of human tumors. Second, the cohort used for clinical validation was relatively small, which may have limited the statistical power and restricted the generalizability of our conclusions. In addition, interpatient heterogeneity, a hallmark of GBM, may contribute to variability in circSPECC1 expression and function, potentially affecting the reproducibility of these observations across independent cohorts. Although our data support a functional role of circSPECC1 in GBM progression and response to therapy, comprehensive clinical validation is still lacking and will be essential to establish its prognostic value and therapeutic relevance. Notably, the IC_50_ values of TMZ observed in our in vitro experiments exceeded the drug concentrations typically achievable in the human brain, underscoring the inherent limitations of in vitro drug sensitivity assays and caution against direct clinical extrapolation. Future studies incorporating larger multicenter patient cohorts and clinically relevant pharmacokinetic models are required to substantiate these findings and further evaluate the feasibility of circSPECC1 as a biomarker and therapeutic target in GBM.

In conclusion, this study establishes circSPECC1 as a critical hypoxia-responsive regulator of GBM progression and therapeutic resistance by orchestrating metabolic adaptation through the IGF2BP2–PGK1 axis. Future investigations will be required to define its cell-type–specific functions within the tumor microenvironment, and to assess the feasibility and safety of circSPECC1-directed interventions in clinically relevant models. Collectively, these insights provide a conceptual framework for integrating circRNA biology into precision therapeutic strategies aimed at improving outcomes for patients with GBM.

## Supplementary information

Supplementary Tables

Supplementary Tables

Uncropped original Western Blot

## Data Availability

All data supporting the findings and uncropped western blots are included within the article or its Supplementary Materials, which are available from the corresponding author upon reasonable request.
