# Supplementary material for: Hypoxia-induced circSPECC1 drives temozolomide resistance in glioblastoma via IGF2BP2-mediated PGK1 mRNA stabilization
Source: Cell Death Dis. 2026 Jun 17;17(1):756. doi: 10.1038/s41419-026-08879-8 (PMC13518933; doi:10.1038/s41419-026-08879-8)
Supplement: Supplementary file 1 [file 41419_2026_8879_MOESM2_ESM.pdf]

**Supplementary Table 1. Sequences of primers and siRNA/shRNA/probe used in this study**

| Gene/RNA | Forward primer (5'→3') / Target sequence | Reverse primer (5'→3') |
|---|---|---|
| **qRT-PCR** | | |
| circlSPECC1(divergent) | GAGAGCTGCGAAGTTCAAGA | AGGTGTTTCGTAGGAGTGGG |
| circlSPECC1(convergent) | TTCAGAAGGGGCAGCAAGTC | CTTGAGGAGCCTGATGTCCG |
| SPECC1 | CGGAAGCCCAACTGGAAATCA | TGCTCAAAGTCGGAAGCATCT |
| PGK1 | GAACAAGGTTAAAGCCGAGCC | GTGGCAGATTGACTCCTACCA |
| GAPDH | AGAAGGCTGGGGCTCATTTG | AGGGGCCATCCACAGTCTTC |
| ACTB | CATGTACGTTGCTATCCAGGC | CTCCTTAATGTCACGCACGAT |
| | | |
| **ChIP** | | |
| Binding site 1 | GCCATAGTATTCATTTTGGGTGAGT | AACAACTCGAGGAGGTGGTG |
| Binding site 2 | CGGCAGGATGCAGATGAAG | CCTTCCAGTTCCCAGCAG |
| | | |
| **RIP** | | |
| PGK1 | ACAAGCTGGACGTTAAAGGGA | CCAAGCAGAATTTGATGCTTGGG |
| | | |
| **siRNA** | | |
| siNC | | |
| si-HIF1A | GTGATGAAAGAATTACCGAAT | |
| si-circSPECC1 | AGTCTGCTGGCCAAGGGGCCT | |
| | GGCCAAGGGGCCTTTACAACA | |
| si-IGF2PB2 | AGTGAAGCTGGAAGCGCATAT | |
| | TTCCCGCATCATCACTCTTAT | |
| shcircSPECC1 | AGTCTGCTGGCCAAGGGGCCT | |
| | GGCCAAGGGGCCTTTACAACA | |
| | | |
| **RNA pulldown probe** | | |
| circSPECC1 pulldown | AGUUGU+UGUAAAGGCCCCU+UGGCCAGCAGAC+UGGA | |

**Supplementary Table 2. Clinicopathological data of 11 paraffin-embedded nontumor brain tissues and 51 glioma tissues used for fluorescence in situ hybridization (FISH)**

| ID | Gender (M=Male; F=Femal) | Age | Histological Diagnosis | Histology grading | FISH Score of circSPECC | Intensity(0 -3) | percentage (0-4) | OS Follow-up time | OS Outcome | ki-67(%) | P53 | IDH1 Mutation | MGMT Methylatio n |
|---|---|---|---|---|---|---|---|---|---|---|---|---|---|
| 01 | M | 44 | Oligodendroglioma | II | 1 | 1 | 1 | 2395 | 0 | 2 | 1 | 0 | 1 |
| 02 | M | 40 | Astrocytoma | II | 4 | 2 | 2 | 2534 | 0 | 4 | 1 | NA | 1 |
| 03 | F | 42 | Oligodendroglioma | II | 6 | 3 | 2 | 1434 | 1 | 1 | 1 | NA | 1 |
| 04 | F | 3 | Astrocytoma | II | 6 | 2 | 3 | 731 | 1 | 3 | 1 | 0 | 0 |
| 05 | F | 32 | Oligodendroglioma | II | 3 | 1 | 3 | 2314 | 0 | 10 | 1 | 0 | 0 |
| 06 | M | 65 | Oligodendroglioma | II | 4 | 2 | 2 | 629 | 1 | 20 | 1 | 0 | 1 |
| 07 | M | 14 | Astrocytoma | II | 2 | 1 | 2 | 2543 | 0 | 5 | 1 | NA | 0 |
| 08 | M | 24 | Oligodendroglioma | II | 3 | 3 | 1 | 2025 | 0 | 3 | 0 | 1 | 1 |
| 09 | M | 32 | Astrocytoma | II | 3 | 3 | 1 | 2018 | 0 | 2 | 0 | 0 | 1 |
| 10 | M | 22 | Astrocytoma | II | 0 | 1 | 0 | 2418 | 0 | 5 | 0 | 0 | 1 |
| 11 | F | 57 | Astrocytoma | II | 1 | 1 | 1 | 2296 | 0 | 10 | 1 | 0 | 1 |
| 12 | F | 56 | Astrocytoma | II | 6 | 3 | 2 | 1215 | 1 | 3 | 1 | 0 | 1 |
| 13 | F | 40 | Astrocytoma | II | 4 | 2 | 2 | 1942 | 0 | 5 | 0 | 0 | 0 |
| 14 | M | 27 | Oligodendroglioma | II | 4 | 2 | 2 | 3199 | 0 | 5 | NA | NA | 1 |
| 15 | M | 41 | Astrocytoma | II | 3 | 3 | 1 | 3521 | 0 | 2 | 0 | NA | 1 |
| 16 | M | 39 | Astrocytoma | II | 2 | 2 | 1 | 3510 | 0 | 1 | NA | NA | 1 |
| 17 | F | 41 | Astrocytoma | II | 6 | 3 | 2 | 1284 | 1 | 15 | 1 | NA | 0 |
| 18 | M | 35 | Astrocytoma | II | 6 | 3 | 2 | 1795 | 1 | 3 | 1 | 1 | 0 |
| 19 | F | 51 | Astrocytoma | II | 1 | 1 | 1 | 1949 | 0 | 7 | 1 | 0 | 0 |
| 20 | M | 44 | Astrocytoma | II | 4 | 2 | 2 | 613 | 1 | NA | 1 | NA | 0 |
| 21 | M | 43 | Astrocytoma | III | 2 | 2 | 1 | 1668 | 1 | 10 | 1 | NA | 1 |
| 22 | F | 35 | Oligodendroglioma | III | 4 | 2 | 2 | 2464 | 0 | 85 | 1 | NA | 0 |
| 23 | M | 22 | Oligodendroglioma | III | 9 | 3 | 3 | 213 | 1 | 5 | 1 | 0 | 0 |
| 24 | M | 54 | Astrocytoma | III | 12 | 3 | 4 | 132 | 1 | 50 | 1 | 1 | 1 |
| 25 | M | 41 | Astrocytoma | III | 6 | 2 | 3 | 959 | 1 | 2 | 0 | NA | 1 |
| 26 | M | 57 | Astrocytoma | III | 9 | 3 | 3 | 40 | 1 | 12.5 | 1 | 0 | 0 |
| 27 | M | 44 | Astrocytoma | III | 6 | 3 | 2 | 1932 | 0 | 10 | 1 | 0 | 1 |
| 28 | M | 36 | Oligodendroglioma | III | 3 | 3 | 1 | 3678 | 0 | 30 | 1 | NA | 1 |
| 29 | F | 34 | Astrocytoma | III | 6 | 2 | 3 | 1946 | 1 | 10 | 1 | NA | 1 |
| 30 | M | 19 | Astrocytoma | III | 4 | 2 | 2 | 2488 | 1 | 30 | 1 | NA | 1 |
| 31 | M | 22 | Astrocytoma | III | 8 | 2 | 4 | 520 | 1 | NA | NA | NA | 1 |
| 32 | F | 42 | Oligodendroglioma | III | 4 | 2 | 2 | 508 | 1 | 7.5 | 0 | NA | 0 |
| 33 | F | 31 | Astrocytoma | III | 2 | 2 | 1 | 4269 | 1 | 30 | 1 | NA | 1 |
| 34 | F | 34 | Glioblastoma | IV | 12 | 3 | 4 | 163 | 1 | 25 | 1 | 0 | 0 |
| 35 | F | 48 | Glioblastoma | IV | 2 | 2 | 1 | 913 | 1 | 90 | 1 | 0 | 1 |
| 36 | M | 66 | Glioblastoma | IV | 9 | 3 | 3 | 362 | 1 | 40 | 1 | NA | 1 |
| 37 | M | 56 | Glioblastoma | IV | 8 | 2 | 4 | 161 | 1 | 80 | 1 | 0 | 0 |

| | | | | | | | | | | | | | |
|---|---|---|---|---|---|---|---|---|---|---|---|---|---|
| 38 | F | 21 | Glioblastoma | IV | 12 | 3 | 4 | 2944 | 1 | 1 | 0 | NA | 1 |
| 39 | M | 73 | Glioblastoma | IV | 12 | 3 | 4 | 31 | 1 | 40 | 1 | NA | 0 |
| 40 | M | 37 | Glioblastoma | IV | 9 | 3 | 3 | 198 | 1 | 50 | 1 | 0 | 0 |
| 41 | M | 71 | Glioblastoma | IV | 6 | 2 | 3 | 204 | 1 | 30 | 1 | 0 | 0 |
| 42 | F | 36 | Glioblastoma | IV | 12 | 3 | 4 | 63 | 1 | 40 | 1 | NA | 1 |
| 43 | M | 52 | Glioblastoma | IV | 4 | 2 | 2 | 201 | 1 | 40 | 1 | 0 | 1 |
| 44 | M | 71 | Glioblastoma | IV | 2 | 2 | 1 | 611 | 1 | 3 | 1 | NA | 1 |
| 45 | M | 48 | Glioblastoma | IV | 6 | 2 | 3 | 207 | 1 | 20 | 1 | 1 | 1 |
| 46 | F | 49 | Glioblastoma | IV | 12 | 3 | 4 | 2527 | 0 | 20 | 1 | NA | 1 |
| 47 | M | 42 | Glioblastoma | IV | 12 | 3 | 4 | 2382 | 0 | 20 | 0 | 0 | 1 |
| 48 | M | 38 | Glioblastoma | IV | 12 | 3 | 4 | 1991 | 1 | 50 | 1 | 1 | 1 |
| 49 | F | 63 | Glioblastoma | IV | 12 | 3 | 4 | 921 | 1 | 20 | 1 | 0 | 0 |
| 50 | M | 41 | Glioblastoma | IV | 9 | 3 | 3 | 3242 | 0 | 30 | 1 | NA | 1 |
| 51 | M | 33 | Glioblastoma | IV | 8 | 2 | 4 | 1973 | 0 | 60 | 1 | 0 | 1 |
| 52 | M | 26 | Non-tumor brain tissues | NA | 0 | 1 | 0 | NA | NA | NA | NA | NA | NA |
| 53 | F | 24 | Non-tumor brain tissues | NA | 0 | 1 | 0 | NA | NA | NA | NA | NA | NA |
| 54 | M | 14 | Non-tumor brain tissues | NA | 2 | 2 | 1 | NA | NA | NA | NA | NA | NA |
| 55 | F | 14 | Non-tumor brain tissues | NA | 0 | 2 | 0 | NA | NA | NA | NA | NA | NA |
| 56 | F | 33 | Non-tumor brain tissues | NA | 1 | 1 | 1 | NA | NA | NA | NA | NA | NA |
| 57 | M | 21 | Non-tumor brain tissues | NA | 0 | 1 | 0 | NA | NA | NA | NA | NA | NA |
| 58 | M | 21 | Non-tumor brain tissues | NA | 0 | 1 | 0 | NA | NA | NA | NA | NA | NA |
| 59 | F | 29 | Non-tumor brain tissues | NA | 2 | 1 | 2 | NA | NA | NA | NA | NA | NA |
| 60 | M | 16 | Non-tumor brain tissues | NA | 2 | 2 | 1 | NA | NA | NA | NA | NA | NA |
| 61 | M | 54 | Non-tumor brain tissues | NA | 1 | 1 | 1 | NA | NA | NA | NA | NA | NA |
| 62 | M | 22 | Non-tumor brain tissues | NA | 0 | 2 | 0 | NA | NA | NA | NA | NA | NA |

**Supplementary Table 3. RNA binding proteins interacted with circSPECC1 identified by RNA-pulldown-MS**

| **Protein Name** | **Accession** | **Description** | **Abundances (Normalized): Probe_circSPECC1** | **Abundances (Normalized): Probe_negative** | **FC** | **Score** |
|---|---|---|---|---|---|---|
| PABPC1 | P11940 | Polyadenylate-binding protein 1 | 887702877 | 561489519 | 1.580979 | 99.8 |
| PABPC4 | Q13310 | Polyadenylate-binding protein 4 | 209930583 | 138799826 | 1.51247 | 58.06 |
| UPF1 | Q92900 | Regulator of nonsense transcrip | 109683824 | 48716028.4 | 2.251494 | 51.87 |
| RPS3 | P23396 | 40S ribosomal protein S3 OS=H | 178300266 | 97763320.2 | 1.823795 | 46.14 |
| IGF2BP3 | O00425 | Insulin-like growth factor 2 mR | 63565947.8 | 31552480.8 | 2.01461 | 20.16 |
| IGF2BP2 | Q9Y6M1 | Insulin-like growth factor 2 mR | 10733335.1 | 6674160.06 | 1.608193 | 8.05 |
| LARP1 | Q6PKG0 | La-related protein 1 OS=Homo | 89823260.4 | 41260056.9 | 2.177003 | 7.98 |
| ELAVL1 | Q15717 | ELAV-like protein 1 OS=Homo | 20216552.2 | 11616303.9 | 1.74036 | 7.82 |
| RBM14 | Q96PK6 | RNA-binding protein 14 OS=H | 45356546.1 | 34341618.5 | 1.320746 | 7.51 |
| CPSF6 | Q16630 | Cleavage and polyadenylation s | 2283188 | 930468.188 | 2.453806 | 4.09 |
| SSB | P05455 | Lupus La protein OS=Homo sa | 7085085.07 | 4444353.19 | 1.594177 | 3.71 |
| GAPDH | P04406 | Glyceraldehyde-3-phosphate de | 4354025.42 | 2322842.5 | 1.874439 | 2.4 |
| HNRNPC | P07910 | Heterogeneous nuclear ribonucl | 7184490.6 | 4923792.91 | 1.459137 | 1.74 |
| RALY | Q9UKM9 | RNA-binding protein Raly OS= | 4651595.3 | 2606077.31 | 1.784903 | 1.66 |
| CPSF7 | Q8N684 | Cleavage and polyadenylation s | 4508527.58 | 1603210.47 | 2.812187 | 0 |
| EIF3B | P55884 | Eukaryotic translation initiation | 3348641.25 | 1626045.13 | 2.059378 | 0 |
| LARP4 | Q71RC2 | La-related protein 4 OS=Homo | 3745659.38 | 2418279.38 | 1.548894 | 0 |
| MATR3 | P43243 | Matrin-3 OS=Homo sapiens OX | 2108838.83 | 960712.563 | 2.195078 | 0 |
| RBM39 | Q14498 | RNA-binding protein 39 OS=H | 15801267.4 | 5587164.25 | 2.828137 | 0 |
| RBM8A | Q9Y5S9 | RNA-binding protein 8A OS=H | 6464899.02 | 4575089.88 | 1.413065 | 0 |
| TNRC6A | Q8NDV7 | Trinucleotide repeat-containing | 1774656.42 | 737943.375 | 2.404868 | 0 |
| U2AF2 | P26368 | Splicing factor U2AF 65 kDa su | 2577190.91 | 592044.813 | 4.353034 | 0 |

**Supplementary Table 4. Differentially expressed genes in pGBM1 cells after circSPECC1 knockdown.**

| Gene ID | Gene Symbol | Chromosome | Start | End | Strand | Length | shNC-1 | shNC-2 | shNC-3 | hcircSPECC1-1 | hcircSPECC1-2 | hcircSPECC1-3 | FC | og2FoldChange | pvalue | padj |
|---|---|---|---|---|---|---|---|---|---|---|---|---|---|---|---|---|
| ENSG00000 | CD74 | 5 | 1.5E+08 | 1.5E+08 | - | 4526 | 69.16 | 67.06 | 68.8 | 25.26 | 24.73 | 25.61 | 0.35471499 | -1.495267795 | 0 | 0 |
| ENSG00000 | PGK1 | X | 77910739 | 78129295 | + | 5437 | 282.65 | 283.33 | 289.43 | 133.69 | 137.49 | 126.69 | 0.44748543 | -1.160087396 | 0 | 0 |
| ENSG00000 | TFPI2 | 7 | 93885396 | 93890753 | - | 2444 | 107.52 | 111.06 | 113.62 | 900.77 | 898.59 | 882.24 | 7.76761645 | 2.957471966 | 0 | 0 |
| ENSG00000 | NAMPT | 7 | 1.06E+08 | 1.06E+08 | - | 23584 | 25.55 | 25.38 | 25.39 | 55.56 | 53.52 | 53.73 | 2.05188509 | 1.036949941 | 0 | 0 |
| ENSG00000 | CA9 | 9 | 35673928 | 35681159 | + | 1598 | 131 | 129.12 | 134.56 | 25.95 | 23.22 | 25.64 | 0.18212439 | -2.457003988 | 0 | 0 |
| ENSG00000 | CSF3 | 17 | 40015361 | 40017813 | + | 2220 | 22.06 | 21.56 | 21.8 | 104.16 | 108.4 | 105.64 | 4.68109978 | 2.226847516 | 0 | 0 |
| ENSG00000 | COL1A1 | 17 | 50184101 | 50201632 | - | 9007 | 101.62 | 103.47 | 103.61 | 27.19 | 29.32 | 27.83 | 0.2630284 | -1.926709537 | 0 | 0 |
| ENSG00000 | ENO2 | 12 | 6913745 | 6923698 | + | 4281 | 69.88 | 73.11 | 73.22 | 30.43 | 29.66 | 28.36 | 0.39347692 | -1.345649063 | 0 | 0 |
| ENSG00000 | TGFBI | 5 | 1.36E+08 | 1.36E+08 | + | 7717 | 331.87 | 337.52 | 339.67 | 144.81 | 142.6 | 138.56 | 0.40611493 | -1.300040039 | 0 | 0 |
| ENSG00000 | IL1B | 2 | 1.13E+08 | 1.13E+08 | - | 3009 | 4.76 | 5.75 | 4.98 | 69.53 | 69.68 | 69.45 | 12.9672821 | 3.696804226 | 0 | 0 |
| ENSG00000 | COL5A1 | 9 | 1.35E+08 | 1.35E+08 | + | 11160 | 36.23 | 36.6 | 36.9 | 10.2 | 10.64 | 9.68 | 0.26760758 | -1.901809111 | 0 | 0 |
| ENSG00000 | MAP1B | 5 | 72107234 | 72209565 | + | 12540 | 23.08 | 23.16 | 22.89 | 56.28 | 55.65 | 55.42 | 2.3287291 | 1.219542823 | 0 | 0 |
| ENSG00000 | LOXL2 | 8 | 23296897 | 23425328 | - | 5985 | 99.84 | 102.16 | 101.91 | 43.69 | 45.96 | 42.15 | 0.41737509 | -1.2605836 | 0 | 0 |
| ENSG00000 | IL36B | 2 | 1.13E+08 | 1.13E+08 | - | 1871 | 66.46 | 61.26 | 61.88 | 185 | 190.68 | 198.23 | 2.91205115 | 1.542035694 | 0 | 0 |
| ENSG00000 | FGF2 | 4 | 1.23E+08 | 1.23E+08 | + | 6775 | 12.58 | 12.48 | 12.61 | 36.8 | 36.52 | 37.01 | 2.81743472 | 1.494382183 | 0 | 0 |
| ENSG00000 | COL6A1 | 21 | 45981770 | 46005050 | + | 5536 | 161.96 | 165.45 | 163.23 | 60.44 | 59.59 | 60.32 | 0.3536152 | -1.499747824 | 0 | 0 |
| ENSG00000 | IGFBP3 | 7 | 45912245 | 45921874 | - | 4569 | 85.98 | 87.83 | 90.04 | 22.19 | 21.93 | 22.86 | 0.24425292 | -2.033552281 | 0 | 0 |
| ENSG00000 | MMP3 | 11 | 1.03E+08 | 1.03E+08 | - | 1854 | 86.21 | 83.97 | 88.02 | 256.2 | 254.51 | 255.06 | 2.85333901 | 1.512651168 | 0 | 0 |
| ENSG00000 | PLOD2 | 3 | 1.46E+08 | 1.46E+08 | - | 7663 | 187.02 | 186.2 | 190.06 | 86.59 | 84.8 | 84.88 | 0.43768361 | -1.192039751 | 0 | 0 |
| ENSG00000 | COL1A2 | 7 | 94394895 | 94431227 | + | 10812 | 77.32 | 77.38 | 78.32 | 37.71 | 38.11 | 37.21 | 0.46675441 | -1.099264456 | 0 | 0 |
| ENSG00000 | FBN1 | 15 | 48408313 | 48645721 | - | 18525 | 36.57 | 36.3 | 35.91 | 14.38 | 14.18 | 14.3 | 0.37897944 | -1.399808506 | 0 | 0 |
| ENSG00000 | CXCL8 | 4 | 73740519 | 73743716 | + | 2588 | 16.29 | 15.95 | 16.98 | 120.64 | 119.18 | 118.31 | 7.00027878 | 2.807412378 | 0 | 0 |
| ENSG00000 | BNIP3 | 10 | 1.32E+08 | 1.32E+08 | - | 4050 | 136.55 | 136.61 | 136.33 | 67.07 | 66.55 | 63.33 | 0.462635 | -1.11205369 | 0 | 0 |
| ENSG00000 | ERO1A | 14 | 52639915 | 52695900 | - | 7549 | 30.8 | 30.97 | 31.58 | 12.12 | 12 | 11.76 | 0.3696812 | -1.435646429 | 0 | 0 |
| ENSG00000 | - | 3 | 44117299 | 44122365 | + | 5067 | 43.63 | 42.63 | 43.96 | 14.41 | 15.72 | 14.35 | 0.32896381 | -1.603999196 | 0 | 0 |
| ENSG00000 | RND3 | 2 | 1.5E+08 | 1.51E+08 | - | 3594 | 81.05 | 78.93 | 76.19 | 169.26 | 168.33 | 169.53 | 2.06554524 | 1.046522658 | .28811334491693e-303 | .31832396104946e-301 |
| ENSG00000 | GNG11 | 7 | 93921735 | 93928610 | + | 3019 | 67.84 | 67.95 | 66.41 | 149.89 | 148.35 | 154.9 | 2.15611306 | 1.108432829 | .17549028724582e-292 | .16917903680765e-289 |
| ENSG00000 | PCOLCE | 7 | 1.01E+08 | 1.01E+08 | + | 3346 | 62.52 | 66.19 | 65 | 23.86 | 24.97 | 24.18 | 0.36282759 | -1.462643942 | .21055709362544e-291 | .17228918121588e-289 |
| ENSG00000 | SEMA7A | 15 | 74409289 | 74433958 | - | 3800 | 74.31 | 78.37 | 77.82 | 35.38 | 36.02 | 35.44 | 0.44611598 | -1.164509282 | .09106870630779e-260 | 4.3041881244031e-257 |
| ENSG00000 | PTGES | 9 | 1.3E+08 | 1.3E+08 | - | 1891 | 18.76 | 18.68 | 15.86 | 71.07 | 71.27 | 67.86 | 3.79199696 | 1.922957809 | .32566567333497e-243 | .13043300331766e-241 |
| ENSG00000 | ARL6IP1 | 16 | 18791669 | 18801572 | - | 3558 | 54.46 | 52.91 | 56.24 | 116.44 | 112.07 | 113.9 | 2.01332542 | 1.009580382 | .51579025599881e-240 | .51981498348992e-237 |
| ENSG00000 | NDRG1 | 8 | 1.33E+08 | 1.33E+08 | - | 12002 | 15.1 | 14.56 | 14.13 | 5.66 | 5.13 | 5.33 | 0.35352633 | -1.500110431 | .64155541323157e-224 | .57994436233562e-221 |
| ENSG00000 | UBE2H | 7 | 1.3E+08 | 1.3E+08 | - | 6514 | 13.03 | 12.14 | 12.42 | 33.67 | 34.1 | 31.39 | 2.53640978 | 1.342787844 | 5.0657966294567e-222 | .05578297911727e-219 |
| ENSG00000 | ADAM12 | 10 | 1.26E+08 | 1.26E+08 | - | 9882 | 18.78 | 19.23 | 19.04 | 9.03 | 8.54 | 8.49 | 0.43925821 | -1.186858833 | .21112007522812e-193 | .39190967955391e-190 |
| ENSG00000 | CAMK2N1 | 1 | 20482391 | 20486210 | - | 2605 | 72.41 | 69.58 | 70.62 | 26.4 | 26.44 | 30.05 | 0.37523455 | -1.414135425 | .02620001797764e-192 | 3.7101795195428e-190 |
| ENSG00000 | SLC16A3 | 17 | 82217934 | 82261129 | + | 9236 | 16.03 | 16.33 | 16.87 | 6.46 | 6.73 | 6.96 | 0.39411305 | -1.343318577 | .93715896476822e-189 | .75796596744143e-187 |
| ENSG00000 | ALDH1A3 | 15 | 1.01E+08 | 1.01E+08 | + | 5335 | 15.73 | 15.64 | 15.42 | 38.98 | 36.56 | 36.42 | 2.30127168 | 1.202431313 | .86121699127068e-189 | .35233275640375e-186 |
| ENSG00000 | CRK | 17 | 1420689 | 1463162 | - | 4064 | 9.61 | 9.57 | 10.22 | 28.47 | 28.53 | 29.08 | 2.81759383 | 1.494463658 | .57647057383928e-181 | .22468622679902e-179 |
| ENSG00000 | SLC38A1 | 12 | 46183063 | 46270017 | - | 10015 | 11.51 | 12.11 | 11.71 | 26.71 | 24.96 | 25.2 | 2.09290735 | 1.065508444 | .79651699055942e-178 | .85635087824164e-176 |
| ENSG00000 | EREG | 4 | 74365145 | 74388749 | + | 5713 | 11.11 | 11.85 | 10.53 | 29.74 | 29.5 | 31.46 | 2.60612592 | 1.381906793 | .56064889155891e-178 | .21266180784661e-176 |
| ENSG00000 | MEST | 7 | 1.3E+08 | 1.31E+08 | + | 4767 | 25.56 | 27.03 | 27.7 | 10.47 | 10.44 | 9.49 | 0.36420639 | -1.457171848 | .30131177812872e-174 | .10739439810946e-172 |
| ENSG00000 | GREM1 | 15 | 32718004 | 32745106 | + | 14784 | 8.17 | 8.07 | 7.81 | 18.23 | 16.84 | 17.67 | 2.10895255 | 1.076526639 | .56527475815229e-170 | .71598311603912e-168 |
| ENSG00000 | ADM | 11 | 10305073 | 10307397 | + | 2325 | 53.46 | 57.34 | 57.96 | 22.62 | 23.01 | 21.11 | 0.38053697 | -1.393891477 | .59146944769213e-167 | .57814264225853e-165 |
| ENSG00000 | EFEMP1 | 2 | 55865967 | 55924139 | - | 3962 | 20.06 | 18.71 | 19.03 | 5.75 | 4.81 | 5.05 | 0.25915829 | -1.948094552 | 9.3994460939071e-162 | .70881138517888e-159 |
| ENSG00000 | DYNLT3 | X | 37836757 | 37847571 | - | 3120 | 29.87 | 28.12 | 28.97 | 63.67 | 62.98 | 66.09 | 2.13214875 | 1.092308093 | .96392395807269e-158 | .69418039866108e-155 |
| ENSG00000 | C5orf15 | 5 | 1.34E+08 | 1.34E+08 | - | 2301 | 73.63 | 73.51 | 74.03 | 37.04 | 37.64 | 36.57 | 0.48399253 | -1.046943313 | .57370311941533e-152 | 1.7906587195832e-149 |
| ENSG00000 | ODC1 | 2 | 10439968 | 10448327 | - | 3307 | 17.73 | 15.86 | 16.83 | 41.56 | 43.63 | 43.97 | 2.46460537 | 1.301356661 | .03530143652049e-151 | .74492920869467e-149 |
| ENSG00000 | GOLGA4 | 3 | 37243191 | 37366879 | + | 10837 | 7.38 | 6.82 | 7.02 | 16.26 | 16.52 | 17.68 | 2.28685144 | 1.19336265 | .61043845944062e-147 | .72958643113127e-144 |
| ENSG00000 | GOLM2 | 15 | 44288719 | 44415758 | + | 7105 | 12.82 | 13.11 | 13.08 | 27.95 | 26.33 | 27.02 | 2.00521054 | 1.00375372 | .35626894945001e-141 | .71395220241628e-139 |

| | | | | | | | | | | | | | | | | | |
|---|---|---|---|---|---|---|---|---|---|---|---|---|---|---|---|---|---|
| ENSG00000 | SLC2A1 | 1 | 42925353 | 42958893 | - | 6614 | 18.8 | 18.27 | 18.85 | 8 | 8.46 | 8.7 | 0.43314273 | -1.207085601 | .40398062231512e-134 | .26421358440866e-131 |
| ENSG00000 | MYL9 | 20 | 36541497 | 36551447 | + | 2808 | 69.61 | 68.48 | 71.66 | 36.64 | 32.13 | 31.24 | 0.4581205 | -1.126200972 | .44082861457165e-132 | .33149732566944e-130 |
| ENSG00000 | SERPINB2 | 18 | 63871692 | 63903888 | + | 2741 | 1.83 | 1.57 | 1.73 | 13.39 | 12.75 | 13.63 | 7.44982923 | 2.897207356 | .11157166750743e-131 | .77143211458924e-129 |
| ENSG00000 | FER1L4 | 20 | 35558737 | 35607562 | - | 10228 | 6.65 | 5.89 | 6.15 | 1.9 | 1.82 | 1.83 | 0.28563882 | -1.807736007 | .04754588798484e-125 | .29306935465138e-122 |
| ENSG00000 | BET1 | 7 | 93962762 | 94004382 | - | 5244 | 7.19 | 7.02 | 7.1 | 17.97 | 18.08 | 19.76 | 2.5195375 | 1.333158926 | .13099765187034e-115 | .39019498340629e-112 |
| ENSG00000 | PAX8-AS1 | 2 | 1.13E+08 | 1.13E+08 | + | 22105 | 1.18 | 1.31 | 1.37 | 3.67 | 3.65 | 3.52 | 2.70831586 | 1.437396005 | .70852836188285e-114 | .26673908423466e-112 |
| ENSG00000 | FAM162A | 3 | 1.22E+08 | 1.22E+08 | + | 11604 | 13.4 | 12.89 | 12.82 | 6.46 | 7.04 | 6.71 | 0.49769584 | -1.006663764 | .37619479564548e-112 | .45760693503871e-110 |
| ENSG00000 | HLA-DRA | 6 | 32439878 | 32445046 | + | 1244 | 86.88 | 86.26 | 79.9 | 38.44 | 36.57 | 36.45 | 0.42343635 | -1.239782981 | 1.3644804463697e-111 | .48923795193224e-109 |
| ENSG00000 | IL13RA2 | X | 1.15E+08 | 1.15E+08 | - | 1626 | 19.46 | 16.91 | 16.13 | 50.89 | 49.85 | 51.68 | 2.79048152 | 1.480514094 | .00369741042586e-109 | .28571000055913e-107 |
| ENSG00000 | VGF | 7 | 1.01E+08 | 1.01E+08 | - | 2680 | 8.24 | 8.43 | 7.56 | 24.08 | 24.85 | 23.83 | 2.88961893 | 1.530879247 | .76109919946486e-107 | .44736703287576e-105 |
| ENSG00000 | IL1RN | 2 | 1.13E+08 | 1.13E+08 | + | 3734 | 1.64 | 1.66 | 1.39 | 8.76 | 9.05 | 8.85 | 5.45809113 | 2.448396483 | .40352604927672e-103 | .68732062940063e-101 |
| ENSG00000 | IMPA1 | 8 | 81656914 | 81686331 | - | 4405 | 3.13 | 3 | 2.84 | 11.78 | 11.9 | 10.31 | 3.64313871 | 1.865181924 | .08377723449755e-101 | 1.78106696760197e-99 |
| ENSG00000 | - | 7 | 94022833 | 94066661 | + | 1064 | 26 | 21.08 | 22.47 | 74.43 | 67.67 | 68.52 | 2.90929899 | 1.540671568 | 1.83051806922531e-99 | 2.98359441037256e-97 |
| ENSG00000 | ECHDC1 | 6 | 1.27E+08 | 1.27E+08 | - | 5676 | 9.84 | 8.39 | 9.41 | 22.43 | 20.65 | 21.81 | 2.25751009 | 1.174732438 | 8.73026534850935e-98 | 1.4113928980901e-95 |
| ENSG00000 | MIF | 22 | 23894383 | 23895227 | + | 730 | 124.06 | 126.99 | 134.75 | 64.56 | 63.01 | 62.19 | 0.47315365 | -1.079619329 | 1.5841673724716e-88 | 2.33341986678501e-86 |
| ENSG00000 | DCAF7 | 17 | 63550222 | 63594286 | + | 12752 | 1.79 | 1.84 | 1.73 | 4.7 | 4.79 | 4.76 | 2.55269094 | 1.35201888 | 4.09511314599971e-86 | 5.9875974197209e-84 |
| ENSG00000 | PFKFB4 | 3 | 48517684 | 48562015 | - | 6107 | 14.91 | 15.31 | 16.01 | 8.05 | 7.13 | 7.09 | 0.46294863 | -1.111075982 | 3.8667357845124e-85 | 5.57174210688616e-83 |
| ENSG00000 | ARRDC4 | 15 | 97960703 | 97973833 | + | 4062 | 24.19 | 23.93 | 23.01 | 11.77 | 12.64 | 12.5 | 0.49949071 | -1.001470249 | 2.17625463896626e-78 | 3.00519607610029e-76 |
| ENSG00000 | OLFML2B | 1 | 1.62E+08 | 1.62E+08 | - | 3430 | 13.51 | 13.51 | 12.95 | 4.93 | 4.37 | 4.94 | 0.34235541 | -1.546433287 | 9.36964215218486e-78 | 1.27613242600134e-75 |
| ENSG00000 | MMP1 | 11 | 1.03E+08 | 1.03E+08 | - | 2738 | 1.47 | 1.46 | 1.49 | 8.37 | 8.64 | 8.68 | 5.60182434 | 2.485896744 | 7.1670375165573e-76 | 9.43818152428755e-74 |
| ENSG00000 | GPS1 | 17 | 82050691 | 82057470 | + | 6145 | 5.52 | 5.39 | 5.67 | 12.01 | 13.09 | 12.19 | 2.16420407 | 1.113836542 | 3.02215695210339e-75 | 3.90231110341402e-73 |
| ENSG00000 | PHLDA3 | 1 | 2.01E+08 | 2.01E+08 | - | 3243 | 15.19 | 15.78 | 16.02 | 34.02 | 35.22 | 30.24 | 2.03707897 | 1.026501911 | 1.85800083249086e-74 | 2.33837636418233e-72 |
| ENSG00000 | MNAT1 | 14 | 60734742 | 60969965 | + | 3877 | 5.08 | 5.62 | 5.14 | 14.97 | 13.27 | 15.63 | 2.66300088 | 1.413052907 | 7.39423979097982e-70 | 8.64908577903728e-68 |
| ENSG00000 | RRAD | 16 | 66921679 | 66925536 | - | 1601 | 19.05 | 20.74 | 20.98 | 46.02 | 46.6 | 42.84 | 2.1451572 | 1.101083373 | 6.68584678246408e-69 | 7.59703218681704e-67 |
| ENSG00000 | FURIN | 15 | 90868588 | 90883564 | + | 4960 | 4.17 | 4.31 | 4.58 | 10.97 | 11.77 | 10.48 | 2.44772801 | 1.291443254 | 2.16203699576189e-68 | 2.42893252320482e-66 |
| ENSG00000 | PNLIPRP3 | 10 | 1.16E+08 | 1.16E+08 | + | 2374 | 6.33 | 6.74 | 7.1 | 18.14 | 19.21 | 19.07 | 2.69420079 | 1.429857376 | 2.61463232625617e-68 | 2.92089684312382e-66 |
| ENSG00000 | LIF | 22 | 30240453 | 30246759 | - | 3871 | 5.15 | 5.77 | 5.72 | 13.95 | 14.05 | 13.58 | 2.40436587 | 1.265656448 | 7.35215534545946e-68 | 8.12208939135896e-66 |
| ENSG00000 | PPFIA4 | 1 | 2.03E+08 | 2.03E+08 | + | 9718 | 2.97 | 3.44 | 3.14 | 0.9 | 0.9 | 0.98 | 0.28106023 | -1.831048765 | 5.41531416868769e-65 | 5.72784692789121e-63 |
| ENSG00000 | ALDOC | 17 | 28573115 | 28576948 | - | 2612 | 16.4 | 18.32 | 17.68 | 7.56 | 6.91 | 6.27 | 0.38039002 | -1.394448685 | 1.11157146360715e-62 | 1.14526417377348e-60 |
| ENSG00000 | ZNF395 | 8 | 28345590 | 28402701 | - | 5808 | 8.68 | 8.51 | 9.37 | 3 | 3.92 | 3.63 | 0.38344157 | -1.382921341 | 3.17913260983315e-62 | 3.24190009982216e-60 |
| ENSG00000 | FPR1 | 19 | 51745172 | 51804115 | - | 2583 | 20.29 | 18.69 | 19.79 | 9.25 | 7.94 | 7.67 | 0.40596168 | -1.300584538 | 1.94846224486684e-61 | 1.94699355473252e-59 |
| ENSG00000 | DEPP1 | 10 | 44970981 | 44978809 | - | 2979 | 10.36 | 9.98 | 10.29 | 3.45 | 2.7 | 3.27 | 0.29517819 | -1.760341982 | 2.52467545906756e-61 | 2.51015857517792e-59 |
| ENSG00000 | APLN | X | 1.3E+08 | 1.3E+08 | - | 3224 | 19.15 | 17.77 | 16.65 | 7.31 | 8.31 | 7.81 | 0.42100616 | -1.248086761 | 2.77777666819829e-61 | 2.74806413169766e-59 |
| ENSG00000 | IL36RN | 2 | 1.13E+08 | 1.13E+08 | + | 3209 | 6.39 | 5.78 | 6.71 | 17.04 | 15.29 | 15.27 | 2.42351364 | 1.277100205 | 4.44063923142987e-59 | 4.18493417616033e-57 |
| ENSG00000 | PLXDC1 | 17 | 39063313 | 39154394 | - | 9255 | 3.2 | 3.09 | 3.17 | 1.1 | 0.94 | 1.01 | 0.3088881 | -1.69484381 | 9.54337417339051e-59 | 8.90938945717701e-57 |
| ENSG00000 | PDK1 | 2 | 1.73E+08 | 1.73E+08 | + | 14845 | 4.31 | 4.38 | 4.5 | 2.25 | 2.28 | 1.87 | 0.4664642 | -1.100161731 | 1.4890779754678e-58 | 1.37722397870591e-56 |
| ENSG00000 | IGFBP4 | 17 | 40443450 | 40457725 | + | 2205 | 22.36 | 19.38 | 20.21 | 8.24 | 8.64 | 8.59 | 0.39557493 | -1.337977109 | 2.61260858565024e-58 | 2.39167671350776e-56 |
| ENSG00000 | BEX1 | X | 1.03E+08 | 1.03E+08 | - | 795 | 9.26 | 8.66 | 9.85 | 32.75 | 33.75 | 33.94 | 3.47971036 | 1.798967226 | 4.79494707475928e-58 | 4.3339782991631e-56 |
| ENSG00000 | C1QL1 | 17 | 44959693 | 44968303 | - | 1527 | 29.72 | 28.39 | 27.2 | 11.48 | 12.66 | 10.53 | 0.39122744 | -1.353920523 | 9.77960998637551e-58 | 8.79943640629308e-56 |
| ENSG00000 | ASS1 | 9 | 1.3E+08 | 1.31E+08 | + | 3018 | 7.72 | 7.46 | 7.6 | 17.17 | 17.04 | 16.82 | 2.15506877 | 1.107733905 | 6.61044014906328e-55 | 5.49994152151144e-53 |
| ENSG00000 | MTMR6 | 13 | 25246222 | 25287488 | - | 5237 | 2.29 | 2.31 | 1.92 | 6.75 | 6.71 | 6.06 | 2.87897643 | 1.525555977 | 1.38270766241093e-53 | 1.10867507528393e-51 |
| ENSG00000 | OLFML2A | 9 | 1.25E+08 | 1.25E+08 | + | 6771 | 7.43 | 7.59 | 7.21 | 3.65 | 2.9 | 3.44 | 0.43140004 | -1.21290179 | 5.17872038207212e-52 | 4.05428562194898e-50 |
| ENSG00000 | IFITM3 | 11 | 319676 | 329475 | - | 2844 | 18.6 | 20.21 | 19.4 | 8.73 | 10.22 | 9.75 | 0.4752664 | -1.073191688 | 2.43980432571864e-50 | 1.83077392516661e-48 |
| ENSG00000 | GALNT10 | 5 | 1.54E+08 | 1.54E+08 | + | 10496 | 2.45 | 2.99 | 2.82 | 5.88 | 5.9 | 5.85 | 2.05710735 | 1.040617081 | 3.13976171370085e-50 | 2.34714893522336e-48 |
| ENSG00000 | ADAM19 | 5 | 1.57E+08 | 1.58E+08 | - | 7704 | 3.3 | 2.96 | 3.34 | 1.11 | 0.9 | 0.93 | 0.29430585 | -1.764611867 | 1.929577225114e-49 | 1.41585398971926e-47 |
| ENSG00000 | ANGPTL4 | 19 | 8363289 | 8374370 | + | 2475 | 11.04 | 10.94 | 9.41 | 3.23 | 2.31 | 3.37 | 0.27223339 | -1.877084054 | 3.52106938122394e-48 | 2.50955070414473e-46 |
| ENSG00000 | CRABP2 | 1 | 1.57E+08 | 1.57E+08 | - | 1082 | 44.98 | 45.57 | 41.19 | 20.49 | 22.39 | 20.04 | 0.45967305 | -1.12132002 | 2.45695208080994e-47 | 1.73866520024575e-45 |
| ENSG00000 | TMEM45A | 3 | 1E+08 | 1.01E+08 | + | 6005 | 6.58 | 7.18 | 6.69 | 2.61 | 3.34 | 2.74 | 0.40985632 | -1.28680985 | 4.89627871790344e-47 | 3.41622815107052e-45 |
| ENSG00000 | OLFML3 | 1 | 1.14E+08 | 1.14E+08 | + | 2821 | 17.37 | 17.33 | 15.65 | 8.4 | 8.11 | 7.35 | 0.45546239 | -1.134596163 | 5.26607396404001e-47 | 3.66139443269005e-45 |
| ENSG00000 | MIR210HG | 11 | 565657 | 568457 | - | 2324 | 9.12 | 8.38 | 9.25 | 2.9 | 2.61 | 2.31 | 0.28030123 | -1.834950002 | 2.35976704605964e-45 | 1.55377376526145e-43 |
| ENSG00000 | PLEKHG4 | 16 | 67277510 | 67289499 | + | 7328 | 6.23 | 6.45 | 6.35 | 3.38 | 3.21 | 2.98 | 0.48339084 | -1.048737951 | 4.28093000968545e-45 | 2.79102600795394e-43 |

| | | | | | | | | | | | | | | | | |
|---|---|---|---|---|---|---|---|---|---|---|---|---|---|---|---|---|
| ENSG00000 | ATP6V0D2 | 8 | 85987323 | 86154225 | + | 2940 | 1.84 | 1.23 | 1.47 | 6.61 | 6.25 | 6.26 | 4.03553023 | 2.012758244 | 1.92621824687484e-43 | 1.20449213330522e-41 |
| ENSG00000 | RPLP0P2 | 11 | 61615036 | 61639449 | + | 4060 | 1.35 | 1.43 | 1.15 | 5.3 | 4.69 | 4.79 | 3.61489566 | 1.853954007 | 2.33284750213265e-43 | 1.44964601812212e-41 |
| ENSG00000 | CCDC30 | 1 | 42463221 | 42657190 | + | 25199 | 0.31 | 0.34 | 0.22 | 1.05 | 0.94 | 1.07 | 3.3717482 | 1.753496802 | 5.1609786545172e-43 | 3.16747100447761e-41 |
| ENSG00000 | DHRS3 | 1 | 12567910 | 12618210 | - | 3515 | 1.76 | 2.48 | 2.26 | 6.58 | 7.44 | 7.59 | 3.21267576 | 1.683775386 | 9.65523970651999e-43 | 5.85348907207774e-41 |
| ENSG00000 | MTSS2 | 16 | 70661204 | 70686053 | - | 4979 | 7.59 | 7.3 | 7.44 | 3.52 | 3.19 | 3.64 | 0.44562025 | -1.166113293 | 3.31311258383494e-42 | 1.96660429043456e-40 |
| ENSG00000 | LEPR | 1 | 65420652 | 65641559 | + | 11370 | 4.13 | 4.35 | 3.71 | 2.09 | 1.79 | 1.62 | 0.43356544 | -1.205678326 | 3.7007557699141e-42 | 2.18366553367187e-40 |
| ENSG00000 | SPAG4 | 20 | 35615829 | 35621094 | + | 2348 | 12.27 | 15.22 | 15.8 | 5.76 | 5.87 | 5.06 | 0.37117457 | -1.429830229 | 5.54612886613731e-42 | 3.23415755141174e-40 |
| ENSG00000 | RPS6KA2 | 6 | 1.66E+08 | 1.67E+08 | - | 8251 | 0.16 | 0.19 | 0.21 | 1.42 | 1.37 | 1.36 | 7.09823538 | 2.827460415 | 1.28519711461331e-41 | 7.40757815190891e-40 |
| ENSG00000 | ESRRA | 11 | 64305497 | 64316743 | + | 3101 | 4.41 | 4.91 | 5.28 | 13.48 | 12.18 | 10.84 | 2.40324339 | 1.264982764 | 4.63433517537749e-40 | 2.55982652673282e-38 |
| ENSG00000 | SERPINB7 | 18 | 63752935 | 63805376 | + | 2637 | 4.91 | 5.22 | 5.12 | 11.85 | 12.56 | 14.59 | 2.46276949 | 1.3002816 | 1.77578730188743e-39 | 9.67439191726889e-38 |
| ENSG00000 | DPYSL4 | 10 | 1.32E+08 | 1.32E+08 | + | 3111 | 5.98 | 6.79 | 6.41 | 2.08 | 2.2 | 1.91 | 0.31061016 | -1.686823084 | 2.30302339354442e-39 | 1.24444620056062e-37 |
| ENSG00000 | TCAF2 | 7 | 1.44E+08 | 1.44E+08 | + | 6410 | 2.48 | 2.23 | 2.51 | 0.67 | 0.52 | 0.65 | 0.24420743 | -2.033821007 | 2.53436976508424e-38 | 1.31926551776702e-36 |
| ENSG00000 | RASSF4 | 10 | 44959407 | 44995891 | + | 9481 | 3.97 | 4.04 | 3.72 | 2.03 | 1.86 | 1.86 | 0.47082631 | -1.086733156 | 1.3427597029243e-37 | 6.88164347748706e-36 |
| ENSG00000 | - | 3 | 44115818 | 44117487 | - | 1670 | 18.95 | 19.48 | 19.62 | 8.67 | 9.71 | 7.4 | 0.42763726 | -1.225540545 | 3.32992371171591e-37 | 1.68487361342165e-35 |
| ENSG00000 | H1-0 | 22 | 37805229 | 37807432 | + | 2204 | 8.4 | 9.38 | 10.36 | 18.93 | 21.32 | 20.04 | 2.06541369 | 1.046430775 | 1.7124254779678e-36 | 8.4705424451218e-35 |
| ENSG00000 | ADAMTS14 | 10 | 70672506 | 70762441 | + | 5566 | 1.67 | 1.77 | 2.21 | 0.25 | 0.29 | 0.25 | 0.13515196 | -2.887345644 | 1.93166124135386e-36 | 9.48421821835099e-35 |
| ENSG00000 | CPTP | 1 | 1324756 | 1328896 | + | 2555 | 4.38 | 4.86 | 6.01 | 12.03 | 13.35 | 13.82 | 2.47691973 | 1.308547114 | 4.0122461662928e-36 | 1.94593939065201e-34 |
| ENSG00000 | LINC02392 | 12 | 90280894 | 90300974 | + | 2841 | 1.82 | 1.84 | 2.38 | 6.62 | 6.79 | 6.65 | 3.19727767 | 1.676844041 | 4.25858472951543e-36 | 2.05538731423336e-34 |
| ENSG00000 | ZP1 | 11 | 60867542 | 60875693 | + | 2456 | 6.18 | 5.29 | 5.92 | 1.29 | 1.76 | 1.07 | 0.22894775 | -2.126909709 | 1.05282251589747e-34 | 4.83495975256841e-33 |
| ENSG00000 | CCR1 | 3 | 46201711 | 46208313 | - | 2646 | 13.65 | 13.86 | 14.66 | 6.98 | 6.59 | 7.73 | 0.48610962 | -1.040646404 | 2.94785167547305e-34 | 1.34754093256969e-32 |
| ENSG00000 | TENM2 | 5 | 1.67E+08 | 1.68E+08 | + | 21297 | 0.92 | 0.8 | 0.86 | 0.32 | 0.29 | 0.25 | 0.32203364 | -1.634716709 | 1.56616894507863e-33 | 6.98279584593914e-32 |
| ENSG00000 | SCUBE3 | 6 | 35213956 | 35253079 | + | 7819 | 1.56 | 1.18 | 0.99 | 0.16 | 0.13 | 0.13 | 0.1108974 | -3.17270256 | 2.40214227412737e-33 | 1.06621873037997e-31 |
| ENSG00000 | NRXN3 | 14 | 78170373 | 79868291 | + | 15534 | 0.6 | 0.66 | 0.56 | 0.13 | 0.08 | 0.08 | 0.14721127 | -2.764039934 | 4.23373568117506e-32 | 1.77237545305613e-30 |
| ENSG00000 | SPINK1 | 5 | 1.48E+08 | 1.48E+08 | - | 1361 | 1.1 | 1.14 | 1.1 | 6.77 | 6.29 | 7.28 | 5.8632451 | 2.551699366 | 9.28011156349165e-31 | 3.71297823823001e-29 |
| ENSG00000 | WNT5B | 12 | 1529891 | 1647212 | + | 4240 | 6.37 | 8.42 | 6.96 | 3 | 3.55 | 2.63 | 0.40619127 | -1.299768851 | 2.7283980132974e-29 | 1.05758663731811e-27 |
| ENSG00000 | CCL2 | 17 | 34255274 | 34257208 | + | 1935 | 15.71 | 15.39 | 17.63 | 8.44 | 8.38 | 8.04 | 0.49084373 | -1.026664305 | 4.42980250558658e-29 | 1.69072212713223e-27 |
| ENSG00000 | C10orf90 | 10 | 1.26E+08 | 1.27E+08 | - | 5659 | 16.26 | 15.13 | 14.21 | 5.78 | 7.32 | 6.76 | 0.41922103 | -1.254217014 | 5.05284783493787e-29 | 1.92482527198735e-27 |
| ENSG00000 | SLC7A11 | 4 | 1.38E+08 | 1.38E+08 | - | 9645 | 0.61 | 0.51 | 0.46 | 1.61 | 1.65 | 1.76 | 3.06375991 | 1.615303244 | 5.56391681349506e-29 | 2.11545862019788e-27 |
| ENSG00000 | LCP1 | 13 | 46125920 | 46211871 | - | 5076 | 1.96 | 1.81 | 1.5 | 4.64 | 4.77 | 4.18 | 2.47923579 | 1.309895486 | 1.4194615506991e-28 | 5.27588653002832e-27 |
| ENSG00000 | GPR137B | 1 | 2.36E+08 | 2.36E+08 | + | 2307 | 0.47 | 0.52 | 0.69 | 3.72 | 3.54 | 3.51 | 6.16078089 | 2.623113227 | 1.87245082182074e-28 | 6.92075921782629e-27 |
| ENSG00000 | UBE2Q2 | 15 | 75843307 | 75901078 | + | 3830 | 3.6 | 4.07 | 3.51 | 8.18 | 7.54 | 8.41 | 2.07637046 | 1.054063866 | 4.27750578081492e-28 | 1.5635698980056e-26 |
| ENSG00000 | MED11 | 17 | 4731428 | 4733608 | + | 1475 | 1.72 | 2.07 | 2.38 | 7.32 | 9.49 | 8.22 | 3.91536482 | 1.969146738 | 7.17524608515652e-28 | 2.60364540882002e-26 |
| ENSG00000 | HDAC9 | 7 | 18086949 | 19002416 | + | 17269 | 0.73 | 0.75 | 0.7 | 1.69 | 1.61 | 1.5 | 2.11026769 | 1.077426016 | 7.53238285822481e-28 | 2.72825925566121e-26 |
| ENSG00000 | CSTA | 3 | 1.22E+08 | 1.22E+08 | + | 1366 | 0.47 | 1.1 | 0.53 | 6.09 | 5 | 6.27 | 7.97230402 | 2.994996727 | 1.49093937201656e-27 | 5.28472895054356e-26 |
| ENSG00000 | PCLO | 7 | 82754012 | 83162930 | - | 22874 | 0.49 | 0.47 | 0.45 | 1.09 | 1.06 | 1.04 | 2.18212947 | 1.125736701 | 3.03279125505376e-27 | 1.06174391033e-25 |
| ENSG00000 | FIBCD1 | 9 | 1.31E+08 | 1.31E+08 | - | 4056 | 3.4 | 3.73 | 3.61 | 1.36 | 1.14 | 1.26 | 0.33662017 | -1.570806482 | 1.06468842078709e-26 | 3.65653354876535e-25 |
| ENSG00000 | LINC01119 | 2 | 46816697 | 46859009 | + | 4240 | 8.17 | 6.73 | 7.75 | 4.03 | 3.58 | 3.92 | 0.48873537 | -1.032874565 | 1.10017387451059e-26 | 3.77188922321432e-25 |
| ENSG00000 | CXCL2 | 4 | 74097040 | 74099196 | - | 1398 | 6.91 | 5.04 | 5.6 | 14.58 | 15.3 | 15.67 | 2.49577227 | 1.319486299 | 1.44566700746117e-26 | 4.94786375961539e-25 |
| ENSG00000 | LRCH2 | X | 1.15E+08 | 1.15E+08 | - | 4953 | 0.99 | 1.11 | 1.11 | 3.3 | 3.2 | 2.87 | 2.80086976 | 1.485874898 | 8.21322793881154e-26 | 2.72200062605446e-24 |
| ENSG00000 | MYEF2 | 15 | 48134632 | 48178353 | - | 13901 | 0.7 | 0.75 | 0.64 | 1.64 | 1.53 | 1.79 | 2.28764979 | 1.193866209 | 1.36495290610076e-25 | 4.46415929898249e-24 |
| ENSG00000 | DACT1 | 14 | 58633967 | 58648321 | + | 4584 | 1.64 | 1.19 | 1.38 | 3.74 | 4.01 | 3.85 | 2.65088021 | 1.406471481 | 1.87503530513385e-25 | 6.08239429732246e-24 |
| ENSG00000 | SEMA3A | 7 | 83955777 | 84492724 | - | 9259 | 0.83 | 1.07 | 1.01 | 2.26 | 2.25 | 2.29 | 2.2532002 | 1.171975502 | 1.4795820753413e-23 | 4.41101792626112e-22 |
| ENSG00000 | SLC16A6 | 17 | 68267026 | 68291267 | - | 4056 | 0.1 | 0.18 | 0.18 | 1.41 | 1.51 | 1.35 | 9.06020562 | 3.179543792 | 1.92384687660803e-23 | 5.66751039131121e-22 |
| ENSG00000 | UNC5B | 10 | 71212570 | 71302864 | + | 6841 | 1.85 | 1.42 | 1.94 | 4.17 | 3.82 | 3.66 | 2.14389106 | 1.100231597 | 2.98891193164885e-23 | 8.70197858870241e-22 |
| ENSG00000 | LINC00520 | 14 | 55781132 | 55796731 | - | 3482 | 1.74 | 2.04 | 1.61 | 4.71 | 4.5 | 5.24 | 2.57696704 | 1.365674085 | 3.69887134548006e-23 | 1.07062673515096e-21 |
| ENSG00000 | TRIM9 | 14 | 50975262 | 51096061 | - | 8945 | 2.51 | 2.23 | 2.56 | 1.2 | 0.98 | 1.23 | 0.44770974 | -1.159364376 | 5.14679349667767e-23 | 1.48324621277443e-21 |
| ENSG00000 | DCC | 18 | 52340197 | 53535903 | + | 13135 | 1.11 | 1.03 | 1.06 | 0.48 | 0.33 | 0.35 | 0.34648699 | -1.529126915 | 6.48271074506284e-23 | 1.85747410901404e-21 |
| ENSG00000 | PPP2R2B | 5 | 1.47E+08 | 1.47E+08 | - | 13610 | 1.61 | 1.72 | 1.61 | 0.67 | 0.86 | 0.86 | 0.46787882 | -1.095793159 | 2.93604224460468e-22 | 8.15407821703409e-21 |
| ENSG00000 | PLEKHG4B | 5 | 92168 | 189972 | + | 13287 | 0.5 | 0.5 | 0.43 | 0.02 | 0.06 | 0.1 | 0.12832152 | -2.96216499 | 4.78122477035312e-22 | 1.3113745456341e-20 |
| ENSG00000 | GCH1 | 14 | 54842008 | 54902826 | - | 2925 | 1.7 | 1.72 | 2.22 | 5.32 | 4.86 | 5.08 | 2.60484253 | 1.381196159 | 1.64526991019768e-21 | 4.43308836914376e-20 |
| ENSG00000 | CTSO | 4 | 1.56E+08 | 1.56E+08 | - | 7908 | 2.63 | 2.36 | 2.98 | 1.41 | 1.16 | 1.08 | 0.44048266 | -1.182842866 | 1.92895601181952e-21 | 5.16248859960043e-20 |

| | | | | | | | | | | | | | | | | |
|---|---|---|---|---|---|---|---|---|---|---|---|---|---|---|---|---|
| ENSG00000 | IL11 | 19 | 55364382 | 55370463 | - | 2678 | 2.66 | 3.38 | 3.06 | 7.85 | 7.99 | 6.27 | 2.33736009 | 1.224880012 | 4.21958312225671e-21 | 1.10987315325496e-19 |
| ENSG00000 | HNMT | 2 | 1.38E+08 | 1.38E+08 | + | 4756 | 2.17 | 2.48 | 2.1 | 4.53 | 4.7 | 5.06 | 2.03731983 | 1.02667248 | 1.63046167503922e-20 | 4.16731753318186e-19 |
| ENSG00000 | IL33 | 9 | 6215786 | 6257983 | + | 3253 | 1.41 | 0.81 | 1.24 | 3.51 | 3.55 | 3.87 | 3.04169388 | 1.604874964 | 1.91778669161883e-19 | 4.57805382507088e-18 |
| ENSG00000 | - | 14 | 61570405 | 61658696 | + | 2861 | 4.97 | 5.16 | 4.11 | 1.86 | 1.57 | 2.12 | 0.37483794 | -1.415661123 | 3.25396585582449e-19 | 7.6213322783357e-18 |
| ENSG00000 | IGFBP1 | 7 | 45888360 | 45893660 | + | 1642 | 8.85 | 9.3 | 8.98 | 3.71 | 3.72 | 4.54 | 0.42514226 | -1.233982428 | 4.09125927793814e-19 | 9.50405265675233e-18 |
| ENSG00000 | CXCL14 | 5 | 1.36E+08 | 1.36E+08 | - | 1975 | 7.26 | 6.79 | 7.17 | 3.12 | 3.32 | 2.8 | 0.41903755 | -1.254848557 | 5.46934850894574e-19 | 1.26315906039937e-17 |
| ENSG00000 | ADA | 20 | 44584896 | 44652252 | - | 12177 | 1.72 | 2.14 | 1.77 | 0.84 | 1.03 | 0.98 | 0.48740871 | -1.036796065 | 5.62422132970591e-19 | 1.29742043087241e-17 |
| ENSG00000 | APOL2 | 22 | 36226209 | 36239954 | - | 3417 | 2.5 | 2.86 | 3.11 | 6.56 | 5.84 | 5.59 | 2.04169392 | 1.029766604 | 2.524765614955e-18 | 5.6157678135772e-17 |
| ENSG00000 | PI3 | 20 | 45174902 | 45176544 | + | 551 | 20.59 | 17.11 | 17.3 | 34.93 | 39.42 | 43.47 | 2.06175513 | 1.043873 | 2.94514715489746e-18 | 6.52163153397951e-17 |
| ENSG00000 | LDAF1 | 16 | 21158377 | 21180616 | + | 2558 | 3.19 | 2.45 | 3 | 8.67 | 6.93 | 5.95 | 2.39179443 | 1.258093399 | 4.67835809028604e-18 | 1.01560208106264e-16 |
| ENSG00000 | HSF4 | 16 | 67164681 | 67169945 | + | 3913 | 3.97 | 3.38 | 4.03 | 1.92 | 1.72 | 1.39 | 0.42356439 | -1.239346796 | 1.09990630663683e-17 | 2.33671334481553e-16 |
| ENSG00000 | DYDC2 | 10 | 80344745 | 80368073 | + | 2465 | 0.48 | 0.74 | 0.48 | 2.55 | 2.5 | 2.67 | 4.37554958 | 2.129464233 | 1.57762759619013e-17 | 3.30919037449797e-16 |
| ENSG00000 | TMEM119 | 12 | 1.09E+08 | 1.09E+08 | - | 3184 | 3.34 | 4.59 | 4.4 | 1.84 | 1.59 | 1.57 | 0.38943976 | -1.360527901 | 2.54948872381657e-17 | 5.25898166733326e-16 |
| ENSG00000 | DCLK1 | 13 | 35768652 | 36131382 | - | 14451 | 0.54 | 0.52 | 0.47 | 1.2 | 1.35 | 0.99 | 2.23447408 | 1.159935309 | 3.3140012009189e-17 | 6.79370246188375e-16 |
| ENSG00000 | MAMDC4 | 9 | 1.37E+08 | 1.37E+08 | + | 5490 | 2.19 | 2.2 | 2.42 | 1.03 | 1.04 | 0.87 | 0.41542871 | -1.267327173 | 3.94038418700253e-17 | 8.00352804479523e-16 |
| ENSG00000 | - | 19 | 28606688 | 28615229 | + | 4778 | 0.94 | 0.7 | 0.99 | 0.07 | 0.14 | 0.05 | 0.09420629 | -3.408032725 | 4.63566458928288e-17 | 9.32997878116297e-16 |
| ENSG00000 | COPG2IT1 | 7 | 1.31E+08 | 1.31E+08 | + | 7947 | 0.98 | 0.89 | 0.93 | 0.25 | 0.31 | 0.29 | 0.29343426 | -1.768890764 | 1.03297076530704e-16 | 2.02770223772265e-15 |
| ENSG00000 | CXCL3 | 4 | 74036589 | 74038807 | - | 2219 | 2.56 | 2.71 | 1.76 | 5.41 | 6.73 | 6.43 | 2.54445303 | 1.347355562 | 1.04818587642878e-16 | 2.05351489190013e-15 |
| ENSG00000 | WASF3 | 13 | 26557683 | 26688948 | + | 5182 | 1.35 | 1.5 | 1.46 | 3.32 | 2.96 | 3.1 | 2.09147871 | 1.064523311 | 1.05859881013001e-16 | 2.07187375388143e-15 |
| ENSG00000 | STARD9 | 15 | 42575606 | 42720998 | + | 20776 | 0.63 | 0.77 | 0.76 | 0.24 | 0.37 | 0.32 | 0.41484231 | -1.269365054 | 1.06420569363155e-16 | 2.08079943145167e-15 |
| ENSG00000 | EIF1AXP1 | 1 | 16685621 | 16686055 | - | 435 | 16.93 | 17.14 | 19.19 | 40.15 | 40.23 | 34.43 | 2.07361885 | 1.052150739 | 2.49154827169091e-16 | 4.74109448637069e-15 |
| ENSG00000 | SH3D21 | 1 | 36306368 | 36329340 | + | 8142 | 1.84 | 1.62 | 1.46 | 0.81 | 0.64 | 0.7 | 0.41999796 | -1.251545771 | 2.59813820527180e-16 | 4.9391948577275e-15 |
| ENSG00000 | IFITM2 | 11 | 303655 | 309397 | + | 1606 | 7 | 7.06 | 5.61 | 2.1 | 2.84 | 2.58 | 0.36819728 | -1.441449117 | 4.82917997119736e-16 | 8.97460221750089e-15 |
| ENSG00000 | PCK2 | 14 | 24094053 | 24110598 | + | 4177 | 1.26 | 1.53 | 2.02 | 4.49 | 3.72 | 3.5 | 2.34240839 | 1.227992625 | 1.00807202352595e-15 | 1.84072655535478e-14 |
| ENSG00000 | GTF2IP20 | 1 | 2.24E+08 | 2.24E+08 | - | 4199 | 3.52 | 3.55 | 3.55 | 1.74 | 1.89 | 1.76 | 0.48919026 | -1.031532405 | 1.92774820097348e-15 | 3.45968167656658e-14 |
| ENSG00000 | BEAN1 | 16 | 66427295 | 66493529 | + | 7681 | 1.69 | 1.63 | 1.69 | 0.9 | 0.64 | 0.75 | 0.43749341 | -1.192666805 | 2.31804040789854e-15 | 4.12660998308527e-14 |
| ENSG00000 | C4orf47 | 4 | 1.85E+08 | 1.85E+08 | + | 1366 | 8.41 | 7.62 | 6 | 2.6 | 2.93 | 2.82 | 0.36465308 | -1.455403524 | 2.84488896043063e-15 | 5.02403347941057e-14 |
| ENSG00000 | LRRC32 | 11 | 76657524 | 76670747 | - | 4459 | 1.91 | 2.43 | 2.24 | 1.01 | 0.75 | 0.77 | 0.36877158 | -1.439200622 | 3.70512245655124e-15 | 6.47989094534049e-14 |
| ENSG00000 | MIRLET7IHG | 12 | 62602752 | 62622213 | + | 8693 | 1.29 | 0.87 | 1.11 | 0.31 | 0.31 | 0.48 | 0.32316588 | -1.629653189 | 4.7110003571304e-15 | 8.17436667552687e-14 |
| ENSG00000 | DAPL1 | 2 | 1.59E+08 | 1.59E+08 | + | 1325 | 2.4 | 3.52 | 3.19 | 0.34 | 0.42 | 0.62 | 0.14557347 | -2.78018068 | 1.38829898799894e-14 | 2.32768342127815e-13 |
| ENSG00000 | LYPLAL1-DT | 1 | 2.19E+08 | 2.19E+08 | - | 6275 | 0.19 | 0.22 | 0.07 | 0.78 | 0.84 | 0.87 | 5.0040293 | 2.323090236 | 1.74000389933042e-14 | 2.89782056433713e-13 |
| ENSG00000 | CSF1R | 5 | 1.5E+08 | 1.5E+08 | - | 5151 | 3.27 | 2.73 | 3.21 | 1.13 | 1.32 | 1.88 | 0.45318862 | -1.141816454 | 9.77519225469262e-14 | 1.54637786781673e-12 |
| ENSG00000 | PLCXD2 | 3 | 1.12E+08 | 1.12E+08 | + | 8974 | 0.12 | 0.08 | 0.11 | 0.45 | 0.57 | 0.46 | 4.74709921 | 2.247046203 | 1.860394881727e-13 | 2.87220125956067e-12 |
| ENSG00000 | SCG5 | 15 | 32641676 | 32697098 | + | 2196 | 3.63 | 2.71 | 3.14 | 7.35 | 6.48 | 6.32 | 2.03892153 | 1.027806252 | 2.72122036872388e-13 | 4.14012754644792e-12 |
| ENSG00000 | RAB39B | X | 1.55E+08 | 1.55E+08 | - | 3413 | 2.42 | 2.73 | 1.75 | 4.26 | 5.4 | 5.53 | 2.12016518 | 1.084176671 | 3.91141806620331e-13 | 5.89231426109491e-12 |
| ENSG00000 | CHAC1 | 15 | 40952962 | 40956512 | + | 2051 | 1.94 | 1.24 | 2.06 | 5.05 | 5.09 | 4.09 | 2.61054856 | 1.384352995 | 4.68420536521288e-13 | 7.02454175620347e-12 |
| ENSG00000 | TRIB3 | 20 | 362835 | 397559 | + | 3291 | 1.48 | 1.43 | 1.32 | 3.11 | 3.83 | 3.03 | 2.26893036 | 1.18201233 | 7.16313875666176e-13 | 1.06139354825797e-11 |
| ENSG00000 | - | 10 | 1.26E+08 | 1.26E+08 | + | 2971 | 2.43 | 3.17 | 2.55 | 0.83 | 1.22 | 1.02 | 0.3639711 | -1.458104211 | 8.4380478939251e-13 | 1.24105460333358e-11 |
| ENSG00000 | HLA-DQA1 | 6 | 32628179 | 32647062 | + | 6883 | 1.43 | 1.38 | 1.58 | 0.67 | 0.75 | 0.57 | 0.43699878 | -1.194298839 | 1.1578280691858e-12 | 1.68299789150289e-11 |
| ENSG00000 | FOS | 14 | 75278826 | 75282230 | + | 3186 | 3 | 2.7 | 2.78 | 1.18 | 1.45 | 0.9 | 0.39980011 | -1.322649227 | 1.54898982951163e-12 | 2.23362311528925e-11 |
| ENSG00000 | - | 6 | 1.36E+08 | 1.36E+08 | - | 8029 | 0.22 | 0.28 | 0.36 | 1.08 | 0.79 | 0.83 | 3.01954996 | 1.594333543 | 1.68571268081732e-12 | 2.41327549733999e-11 |
| ENSG00000 | HLA-F | 6 | 29722775 | 29738528 | + | 4315 | 2.7 | 2.9 | 2.48 | 1.34 | 1.35 | 1.35 | 0.48085454 | -1.056327552 | 2.18088293860801e-12 | 3.0844137435434e-11 |
| ENSG00000 | PAPPA2 | 1 | 1.76E+08 | 1.77E+08 | + | 10926 | 0.36 | 0.3 | 0.25 | 0.81 | 0.74 | 0.82 | 2.49501894 | 1.319050769 | 2.82482915054108e-12 | 3.96133481371716e-11 |
| ENSG00000 | EVI2B | 17 | 31303770 | 31314105 | - | 2061 | 1.93 | 1.66 | 1.75 | 4.04 | 4.18 | 5.29 | 2.43367254 | 1.28313506 | 3.26720902381311e-12 | 4.54961144527476e-11 |
| ENSG00000 | SH3BGR | 21 | 39445855 | 39515506 | + | 1448 | 2.51 | 2.32 | 1.6 | 5.83 | 5.33 | 6.24 | 2.59815237 | 1.377486038 | 3.82355555634674e-12 | 5.30205036526882e-11 |
| ENSG00000 | RILPL2 | 12 | 1.23E+08 | 1.23E+08 | - | 1752 | 4.03 | 4.38 | 3.12 | 7.5 | 8.66 | 7.85 | 2.00498835 | 1.003593855 | 5.57819483041853e-12 | 7.65510035906643e-11 |
| ENSG00000 | LINC01326 | 3 | 1.67E+08 | 1.67E+08 | - | 755 | 0.07 | 0.48 | 0.14 | 2.94 | 3.53 | 3.71 | 14.488819 | 3.856868101 | 8.86509446480471e-12 | 1.2016523751373e-10 |
| ENSG00000 | FLG-AS1 | 1 | 1.52E+08 | 1.52E+08 | + | 14980 | 0.09 | 0.08 | 0.16 | 0.37 | 0.44 | 0.37 | 3.38117932 | 1.75752653 | 8.90194210083634e-12 | 1.20500421153935e-10 |
| ENSG00000 | INAFM1 | 19 | 47274885 | 47275723 | + | 839 | 14.65 | 23.4 | 20.51 | 9.54 | 8.48 | 11.04 | 0.47784588 | -1.065382704 | 1.20219192046757e-11 | 1.61197480367483e-10 |
| ENSG00000 | - | 12 | 93640822 | 93641586 | - | 765 | 10.93 | 10.97 | 13.2 | 5.48 | 5.45 | 5.03 | 0.43740266 | -1.192966116 | 1.65556817929375e-11 | 2.18598759928661e-10 |
| ENSG00000 | - | 3 | 1.46E+08 | 1.46E+08 | - | 2095 | 3.14 | 2.18 | 2.09 | 0.97 | 0.56 | 0.5 | 0.26091285 | -1.938360088 | 1.78788225706143e-11 | 2.35132530963403e-10 |

| | | | | | | | | | | | | | | | | |
|---|---|---|---|---|---|---|---|---|---|---|---|---|---|---|---|---|
| ENSG00000 | SP6 | 17 | 47844908 | 47855874 | - | 4045 | 3.1 | 3.12 | 3.03 | 1.22 | 1.7 | 1.82 | 0.49491324 | -1.014752452 | 2.13811500056315e-11 | 2.78796175647202e-10 |
| ENSG00000 | ARTN | 1 | 43933320 | 43937240 | + | 2657 | 2.83 | 4.19 | 3.98 | 1.72 | 1.28 | 1.73 | 0.41336391 | -1.274515664 | 2.4595965776915e-11 | 3.19458379800101e-10 |
| ENSG00000 | TNFSF14 | 19 | 6661253 | 6670588 | - | 4780 | 0.3 | 0.23 | 0.22 | 1.2 | 0.8 | 0.9 | 3.72794063 | 1.898378883 | 5.14822643270432e-11 | 6.45475930733452e-10 |
| ENSG00000 | LINC01807 | 2 | 2.28E+08 | 2.29E+08 | - | 1947 | 0.08 | 0.08 | 0.24 | 1.7 | 0.94 | 1.44 | 9.94750038 | 3.314334049 | 5.48609830442642e-11 | 6.86971440702263e-10 |
| ENSG00000 | CXCL5 | 4 | 73995642 | 73998677 | - | 2436 | 0.47 | 0.38 | 0.36 | 1.49 | 1.46 | 2.25 | 4.1227732 | 2.043615098 | 6.41630949942096e-11 | 7.9543836905228e-10 |
| ENSG00000 | PRSS3 | 9 | 33750679 | 33799231 | + | 1425 | 1.36 | 1.34 | 1.41 | 3.83 | 3.9 | 4.46 | 2.85045642 | 1.511192946 | 6.46234884236678e-11 | 8.00646770906315e-10 |
| ENSG00000 | - | 21 | 44331234 | 44335851 | + | 4354 | 1.57 | 1.49 | 1.53 | 0.69 | 0.65 | 0.29 | 0.34153234 | -1.549905894 | 7.30686766034365e-11 | 9.03027118868449e-10 |
| ENSG00000 | FBLN5 | 14 | 91869411 | 91947987 | - | 4845 | 0.15 | 0.25 | 0.23 | 0.81 | 0.79 | 0.94 | 3.86877941 | 1.95187847 | 8.65691244903042e-11 | 1.06326562105602e-09 |
| ENSG00000 | FCRLA | 1 | 1.62E+08 | 1.62E+08 | + | 3275 | 0.94 | 0.89 | 1.1 | 2.95 | 2.58 | 1.98 | 2.46016486 | 1.298754997 | 1.05100983527546e-10 | 1.28216751990506e-09 |
| ENSG00000 | ANK1 | 8 | 41653220 | 41896762 | - | 10797 | 1.52 | 1.1 | 1.34 | 0.72 | 0.78 | 0.47 | 0.47979092 | -1.059522243 | 1.09489625040675e-10 | 1.33325241514624e-09 |
| ENSG00000 | WFDC3 | 20 | 45747944 | 45791932 | - | 2405 | 3.56 | 3.81 | 4.03 | 1.87 | 1.89 | 1.79 | 0.46966763 | -1.090287943 | 1.11307131540075e-10 | 1.35372618389871e-09 |
| ENSG00000 | RNASE1 | 14 | 20801228 | 20802855 | - | 1083 | 8.73 | 6.12 | 8.61 | 3.99 | 2.46 | 3.28 | 0.39709354 | -1.332449214 | 1.14514169762009e-10 | 1.39017964940021e-09 |
| ENSG00000 | TNFSF13B | 13 | 1.08E+08 | 1.08E+08 | + | 3059 | 3.14 | 3.62 | 3.34 | 1.76 | 1.58 | 1.81 | 0.49082248 | -1.026726758 | 1.20665511460548e-10 | 1.45950954707603e-09 |
| ENSG00000 | PHETA1 | 12 | 1.11E+08 | 1.11E+08 | - | 3361 | 0.99 | 0.86 | 0.98 | 2.46 | 2.05 | 2.46 | 2.3608508 | 1.239306873 | 1.46724169709557e-10 | 1.76077858459538e-09 |
| ENSG00000 | CHRDL1 | X | 1.11E+08 | 1.11E+08 | - | 3920 | 1.54 | 1.64 | 1.79 | 0.7 | 0.77 | 0.49 | 0.37875867 | -1.400649185 | 1.58790554475972e-10 | 1.89870726142797e-09 |
| ENSG00000 | CSDC2 | 22 | 41561010 | 41577741 | + | 3003 | 0.25 | 0.5 | 0.55 | 1.67 | 1.79 | 1.32 | 3.55819195 | 1.831144342 | 2.03485368836855e-10 | 2.40995030334774e-09 |
| ENSG00000 | - | 11 | 35014075 | 35067192 | - | 3401 | 0.51 | 0.49 | 0.47 | 1.87 | 1.81 | 1.13 | 3.14495583 | 1.653039756 | 2.62944209206882e-10 | 3.08111113734758e-09 |
| ENSG00000 | FGF7 | 15 | 49423237 | 49488775 | + | 6516 | 0.72 | 0.56 | 0.55 | 1.61 | 1.08 | 2.01 | 2.47256045 | 1.306005795 | 3.86251556005134e-10 | 4.46028582529738e-09 |
| ENSG00000 | - | 4 | 1.12E+08 | 1.12E+08 | + | 4813 | 0.34 | 0.43 | 0.47 | 1.02 | 1.57 | 1.13 | 2.88410543 | 1.528123903 | 4.77285735177573e-10 | 5.44198786926952e-09 |
| ENSG00000 | SCG2 | 2 | 2.24E+08 | 2.24E+08 | - | 2577 | 3.84 | 3.86 | 4.58 | 2.42 | 2.22 | 1.74 | 0.49890423 | -1.003165201 | 7.88639333149982e-10 | 8.75116804670055e-09 |
| ENSG00000 | GABRG3-AS1 | 15 | 27038978 | 27161319 | - | 2864 | 3.3 | 3.35 | 3.72 | 1.37 | 1.67 | 2.12 | 0.48020767 | -1.058269635 | 8.00614531836867e-10 | 8.8790964671367e-09 |
| ENSG00000 | MUC1 | 1 | 1.55E+08 | 1.55E+08 | - | 2401 | 1.33 | 1.25 | 1.61 | 0.48 | 0.3 | 0.22 | 0.22671922 | -2.141021397 | 8.15127713343984e-10 | 9.02998026732319e-09 |
| ENSG00000 | MIR3681HG | 2 | 11833926 | 12702913 | + | 9856 | 0.88 | 0.8 | 1.04 | 0.39 | 0.51 | 0.41 | 0.46663584 | -1.099630976 | 9.51046246717297e-10 | 1.04541484886531e-08 |
| ENSG00000 | TUSC3 | 8 | 15417215 | 15766649 | + | 5190 | 0.49 | 0.49 | 0.59 | 1.25 | 1.84 | 1.11 | 2.5895106 | 1.372679464 | 9.87696536415021e-10 | 1.08330643279717e-08 |
| ENSG00000 | DNAH10 | 12 | 1.24E+08 | 1.24E+08 | + | 16234 | 0.25 | 0.15 | 0.2 | 0.64 | 0.45 | 0.45 | 2.47786801 | 1.309099339 | 1.01455778922679e-09 | 1.1109296056594e-08 |
| ENSG00000 | - | 3 | 11193439 | 11225877 | - | 1000 | 1.39 | 1.35 | 0.57 | 3.87 | 4.22 | 4.49 | 3.65588996 | 1.870222647 | 1.07676918987058e-09 | 1.17840150470977e-08 |
| ENSG00000 | KCNS1 | 20 | 45091214 | 45101127 | - | 5649 | 0.85 | 0.76 | 0.93 | 0.22 | 0.37 | 0.27 | 0.32959146 | -1.601249223 | 1.2805551247195e-09 | 1.38465680560344e-08 |
| ENSG00000 | GBAP1 | 1 | 1.55E+08 | 1.55E+08 | - | 4163 | 2.34 | 2.24 | 2.07 | 1.02 | 1.28 | 1.09 | 0.49223606 | -1.022577741 | 1.62229384941266e-09 | 1.73998453050543e-08 |
| ENSG00000 | PCDHB7 | 5 | 1.41E+08 | 1.41E+08 | + | 3740 | 1.64 | 1.97 | 1.9 | 0.82 | 0.92 | 0.7 | 0.42662063 | -1.228974365 | 1.80235611885112e-09 | 1.9227388102658e-08 |
| ENSG00000 | ALOXE3 | 17 | 8095900 | 8119047 | - | 3761 | 0.19 | 0.22 | 0.29 | 0.96 | 1.05 | 0.79 | 3.89410831 | 1.96129301 | 1.83802109157514e-09 | 1.95973455259902e-08 |
| ENSG00000 | CCDC178 | 18 | 32937402 | 33441101 | - | 4475 | 1.21 | 1.47 | 1.3 | 0.54 | 0.51 | 0.6 | 0.39800827 | -1.329129697 | 1.95679895790173e-09 | 2.0830271562032e-08 |
| ENSG00000 | - | 7 | 76071469 | 76074963 | - | 3495 | 1.15 | 0.84 | 0.77 | 2.25 | 2.38 | 1.98 | 2.29988201 | 1.20155985 | 2.11493644359412e-09 | 2.24535564233151e-08 |
| ENSG00000 | TNFAIP6 | 2 | 1.51E+08 | 1.51E+08 | + | 1577 | 2.15 | 2.4 | 3.27 | 5.44 | 5.46 | 6.47 | 2.14319368 | 1.099762231 | 2.67061701031055e-09 | 2.8097999603188e-08 |
| ENSG00000 | LINC00601 | 10 | 1.26E+08 | 1.26E+08 | - | 1168 | 4.52 | 6.56 | 5.74 | 2.83 | 2.33 | 1.6 | 0.38538307 | -1.375634886 | 2.79679820617021e-09 | 2.9378939424033e-08 |
| ENSG00000 | GNB3 | 12 | 6839954 | 6847393 | + | 3437 | 1.82 | 1.64 | 2.14 | 0.65 | 0.81 | 0.92 | 0.40793016 | -1.293605917 | 2.85990074796486e-09 | 3.00100930729716e-08 |
| ENSG00000 | NPL | 1 | 1.83E+08 | 1.83E+08 | + | 4975 | 0.77 | 0.91 | 0.55 | 1.75 | 1.64 | 1.68 | 2.18860156 | 1.130009332 | 3.88602902860967e-09 | 4.0079713295593e-08 |
| ENSG00000 | LINC03024 | 8 | 1.34E+08 | 1.34E+08 | - | 609 | 0.41 | 0.09 | 0.68 | 3.34 | 2.74 | 4.69 | 8.84545173 | 3.144935822 | 4.50894467369108e-09 | 4.62166829053336e-08 |
| ENSG00000 | - | 1 | 70947379 | 70951493 | + | 749 | 0.66 | 0.76 | 0.21 | 2.8 | 3.26 | 4.05 | 5.95931149 | 2.575145659 | 5.53395987342001e-09 | 5.62303485349806e-08 |
| ENSG00000 | NR3C2 | 4 | 1.48E+08 | 1.48E+08 | - | 6607 | 0.41 | 0.45 | 0.33 | 1.09 | 1.04 | 0.83 | 2.39075631 | 1.257467084 | 6.89241382826404e-09 | 6.91501760721646e-08 |
| ENSG00000 | PTER | 10 | 16436943 | 16513745 | + | 4003 | 0.51 | 0.39 | 0.5 | 1.06 | 1.83 | 1.22 | 2.83568161 | 1.503695554 | 8.27560728645902e-09 | 8.26106681180912e-08 |
| ENSG00000 | ADAM32 | 8 | 39106990 | 39284917 | + | 10050 | 0.61 | 0.59 | 0.4 | 0.24 | 0.17 | 0.2 | 0.36279298 | -1.462781548 | 9.49829137052156e-09 | 9.40137003000604e-08 |
| ENSG00000 | - | X | 1.35E+08 | 1.35E+08 | + | 2227 | 3.66 | 3.86 | 3.1 | 1.94 | 1.57 | 1.75 | 0.47537465 | -1.072863131 | 1.0315550592995e-08 | 1.01546892842428e-07 |
| ENSG00000 | WFDC21P | 17 | 60083562 | 60091885 | - | 1103 | 2.84 | 1.74 | 2.66 | 6.28 | 6.75 | 5.13 | 2.40774449 | 1.267682301 | 1.06596755678082e-08 | 1.04727099143215e-07 |
| ENSG00000 | OSR1 | 2 | 19351485 | 19358623 | - | 5279 | 1.53 | 1.81 | 1.4 | 0.52 | 0.64 | 0.98 | 0.43733344 | -1.193194437 | 1.06971618856441e-08 | 1.05043488442485e-07 |
| ENSG00000 | MLXIPL | 7 | 73593194 | 73624543 | - | 4815 | 0.12 | 0.08 | 0.05 | 0.47 | 0.62 | 0.41 | 5.72392639 | 2.51700512 | 1.18767885456502e-08 | 1.16168194899289e-07 |
| ENSG00000 | IL1A | 2 | 1.13E+08 | 1.13E+08 | - | 2017 | 1.11 | 0.46 | 0.46 | 2.96 | 2.26 | 1.96 | 3.37774727 | 1.756061388 | 1.24607382998596e-08 | 1.21640540546248e-07 |
| ENSG00000 | FAM107B | 10 | 14518557 | 14774897 | - | 7019 | 0.19 | 0.18 | 0.3 | 0.58 | 0.82 | 0.63 | 2.9308443 | 1.551316325 | 1.47277155106652e-08 | 1.42789187191408e-07 |
| ENSG00000 | MGAM | 7 | 1.42E+08 | 1.42E+08 | + | 10767 | 0.02 | 0.02 | 0.05 | 0.18 | 0.18 | 0.27 | 6.33590315 | 2.663550282 | 1.71326969478202e-08 | 1.64900134950341e-07 |
| ENSG00000 | PRRT2 | 16 | 29811382 | 29815892 | + | 3755 | 2.16 | 2.87 | 2.66 | 0.98 | 1.54 | 1.4 | 0.49248219 | -1.021856538 | 1.72538823228522e-08 | 1.65745628014452e-07 |
| ENSG00000 | MPP3 | 17 | 43800799 | 43833170 | - | 4491 | 1.7 | 1.83 | 1.92 | 1.07 | 0.83 | 0.92 | 0.49784414 | -1.006233939 | 2.10672431106077e-08 | 1.99962830193047e-07 |
| ENSG00000 | SNED1 | 2 | 2.41E+08 | 2.41E+08 | + | 13812 | 0.42 | 0.46 | 0.43 | 0.21 | 0.19 | 0.2 | 0.43991152 | -1.184714699 | 2.25146400545404e-08 | 2.1319219880216e-07 |

| | | | | | | | | | | | | | | | | |
|---|---|---|---|---|---|---|---|---|---|---|---|---|---|---|---|---|
| ENSG00000 | PCDHB14 | 5 | 1.41E+08 | 1.41E+08 | + | 4417 | 1.33 | 1.93 | 1.76 | 0.76 | 0.93 | 0.63 | 0.44634511 | -1.163768476 | 2.34030444750892e-08 | 2.20973190592189e-07 |
| ENSG00000 | RHBDL3 | 17 | 32265832 | 32324659 | + | 5746 | 0.41 | 0.36 | 0.35 | 0.07 | 0.07 | 0.06 | 0.16769057 | -2.576126556 | 2.37738753558636e-08 | 2.24368064286354e-07 |
| ENSG00000 | MMP12 | 11 | 1.03E+08 | 1.03E+08 | - | 1822 | 0.87 | 1.34 | 1.3 | 2.86 | 2.81 | 3.52 | 2.52258962 | 1.334905526 | 2.38063681172229e-08 | 2.24568135678832e-07 |
| ENSG00000 | SERPIND1 | 22 | 20774113 | 20787720 | + | 2319 | 1.07 | 1.16 | 0.82 | 2.88 | 2.18 | 2.64 | 2.41688128 | 1.273146607 | 2.48983008121809e-08 | 2.34091116619488e-07 |
| ENSG00000 | ERC2 | 3 | 55508311 | 56469095 | - | 8219 | 0.05 | 0.04 | 0.06 | 0.28 | 0.27 | 0.28 | 5.32899326 | 2.413863007 | 2.52647322671537e-08 | 2.37311856935452e-07 |
| ENSG00000 | ACP3 | 3 | 1.32E+08 | 1.32E+08 | + | 4280 | 1.9 | 2.37 | 2.4 | 1.07 | 0.92 | 1.46 | 0.49821866 | -1.005149053 | 3.72706268095704e-08 | 3.43591290731714e-07 |
| ENSG00000 | SYTL5 | X | 38006553 | 38128819 | + | 4820 | 0.88 | 0.84 | 0.82 | 0.28 | 0.39 | 0.24 | 0.34619646 | -1.530337102 | 4.04449835387948e-08 | 3.70963329183087e-07 |
| ENSG00000 | NNT-AS1 | 5 | 43571594 | 43603230 | - | 6987 | 0.28 | 0.32 | 0.38 | 0.81 | 0.74 | 0.87 | 2.37417824 | 1.247428247 | 4.51579392099219e-08 | 4.10029964013377e-07 |
| ENSG00000 | - | 13 | 76552807 | 76846524 | - | 7045 | 0.04 | 0.04 | 0.11 | 0.29 | 0.35 | 0.4 | 5.08749114 | 2.346954375 | 5.6507400487801e-08 | 5.06833404916519e-07 |
| ENSG00000 | PTGFR | 1 | 78303884 | 78540701 | + | 5764 | 0.38 | 0.4 | 0.42 | 0.03 | 0.13 | 0.06 | 0.18606438 | -2.426126168 | 5.71425073949463e-08 | 5.12067940310278e-07 |
| ENSG00000 | CACHD1 | 1 | 64470129 | 64693058 | + | 7022 | 0.4 | 0.34 | 0.4 | 0.78 | 0.88 | 0.99 | 2.22009215 | 1.15061956 | 8.11010790985574e-08 | 7.1326623523875e-07 |
| ENSG00000 | THBD | 20 | 23045633 | 23049672 | - | 4040 | 0.23 | 0.19 | 0.43 | 0.93 | 1.25 | 0.76 | 3.30062656 | 1.72273992 | 8.69090325502647e-08 | 7.6098023437341e-07 |
| ENSG00000 | WDR48 | 3 | 39052013 | 39096671 | + | 7473 | 0.67 | 0.56 | 1.05 | 1.93 | 2.38 | 2.21 | 2.75081641 | 1.459859858 | 9.4329692916952e-08 | 8.21254791442027e-07 |
| ENSG00000 | TRABD2B | 1 | 47760528 | 47997385 | - | 7524 | 0.46 | 0.61 | 0.44 | 0.2 | 0.14 | 0.18 | 0.33273541 | -1.587552701 | 9.80697201376839e-08 | 8.52323594815492e-07 |
| ENSG00000 | MAGED4 | X | 52184876 | 52192268 | + | 4756 | 0.69 | 0.69 | 0.95 | 0.17 | 0.33 | 0.27 | 0.32072525 | -1.640590158 | 1.10536914946487e-07 | 9.54833429066418e-07 |
| ENSG00000 | NME5 | 5 | 1.38E+08 | 1.38E+08 | - | 2389 | 1.67 | 0.85 | 1.62 | 2.87 | 3.3 | 3.32 | 2.21078535 | 1.144558955 | 1.23330032535848e-07 | 1.06165268267331e-06 |
| ENSG00000 | TRPM8 | 2 | 2.34E+08 | 2.34E+08 | + | 7928 | 0.77 | 0.73 | 0.75 | 0.43 | 0.34 | 0.28 | 0.45173964 | -1.146436575 | 1.42501761089512e-07 | 1.22139979278661e-06 |
| ENSG00000 | GUSBP11 | 22 | 23638487 | 23717356 | - | 7764 | 0.67 | 0.75 | 0.6 | 0.29 | 0.29 | 0.33 | 0.4330217 | -1.207488779 | 1.74894796102912e-07 | 1.48750343049889e-06 |
| ENSG00000 | G0S2 | 1 | 2.1E+08 | 2.1E+08 | + | 876 | 6.36 | 6.92 | 8.12 | 3.19 | 4.25 | 2.66 | 0.45552437 | -1.134399867 | 2.16508419630352e-07 | 1.81274523130508e-06 |
| ENSG00000 | RCN1P2 | 13 | 45390353 | 45391267 | - | 915 | 1.9 | 2.94 | 2.03 | 0.62 | 0.3 | 0.45 | 0.19043164 | -2.392654882 | 2.39313083308571e-07 | 1.99444285900709e-06 |
| ENSG00000 | ZNF337 | 20 | 25673195 | 25696853 | - | 4855 | 1 | 0.94 | 0.58 | 0.33 | 0.31 | 0.29 | 0.35099349 | -1.51048382 | 2.4691669026591e-07 | 2.05436752549692e-06 |
| ENSG00000 | - | 20 | 35262727 | 35264187 | - | 1461 | 4.02 | 4.43 | 4.13 | 1.35 | 1.67 | 2.67 | 0.43727072 | -1.193401355 | 2.52259942494207e-07 | 2.09619262703607e-06 |
| ENSG00000 | FDCSP | 4 | 70226124 | 70235252 | + | 513 | 2.13 | 2.73 | 2.81 | 8.05 | 6.39 | 7.73 | 2.77913121 | 1.474633949 | 2.91701175658897e-07 | 2.40284916237662e-06 |
| ENSG00000 | DLX5 | 7 | 97020396 | 97024950 | - | 1744 | 3.99 | 3.15 | 3.37 | 2.08 | 1.53 | 1.47 | 0.46225238 | -1.113247361 | 3.0510441841641e-07 | 2.50806174460947e-06 |
| ENSG00000 | CA2 | 8 | 85463968 | 85481493 | + | 2007 | 0.57 | 0.44 | 0.28 | 1.55 | 1.61 | 1.36 | 3.35813313 | 1.747659427 | 3.17589116246522e-07 | 2.59994218878637e-06 |
| ENSG00000 | PSG5 | 19 | 43166256 | 43186536 | - | 4678 | 0.36 | 0.4 | 0.28 | 0.05 | 0.05 | 0.04 | 0.12877994 | -2.957020231 | 3.64368568749814e-07 | 2.95854184956719e-06 |
| ENSG00000 | KCNN3 | 1 | 1.55E+08 | 1.55E+08 | - | 13599 | 0.02 | 0 | 0.01 | 0.14 | 0.09 | 0.1 | 10.663999 | 3.414676649 | 4.00627986351121e-07 | 3.23188108618711e-06 |
| ENSG00000 | STXBP5L | 3 | 1.21E+08 | 1.21E+08 | + | 11085 | 0.19 | 0.08 | 0.15 | 0.42 | 0.39 | 0.37 | 2.70903052 | 1.437776647 | 4.12365331600101e-07 | 3.31308469449212e-06 |
| ENSG00000 | IGFBP5 | 2 | 2.17E+08 | 2.17E+08 | - | 6466 | 1.49 | 1.52 | 1.09 | 0.77 | 0.48 | 0.86 | 0.49224137 | -1.022562174 | 4.43187583700888e-07 | 3.55354237979523e-06 |
| ENSG00000 | SLC22A25 | 11 | 63158437 | 63243599 | - | 10252 | 0.01 | 0.02 | 0.03 | 0.23 | 0.11 | 0.14 | 8.29267141 | 3.051836927 | 4.83832921399943e-07 | 3.85611929540595e-06 |
| ENSG00000 | PCDHGA1 | 5 | 1.41E+08 | 1.42E+08 | + | 6303 | 0.66 | 0.81 | 0.67 | 0.24 | 0.37 | 0.3 | 0.40891349 | -1.290132433 | 5.28205672424508e-07 | 4.18460948054237e-06 |
| ENSG00000 | C1QTNF6 | 22 | 37180166 | 37199385 | - | 7144 | 0.84 | 0.92 | 1.24 | 0.55 | 0.53 | 0.47 | 0.4975347 | -1.007130955 | 6.49663203246047e-07 | 5.09005232330482e-06 |
| ENSG00000 | LHX8 | 1 | 75128434 | 75161533 | + | 4471 | 0.42 | 0.68 | 0.58 | 1.14 | 1.49 | 1.23 | 2.21024206 | 1.14420438 | 7.57294160834786e-07 | 5.88694073033609e-06 |
| ENSG00000 | KCNK15 | 20 | 44745865 | 44752313 | + | 2514 | 0.61 | 0.68 | 0.41 | 0 | 0.02 | 0.05 | 0.03959416 | -4.658568432 | 8.02406248042652e-07 | 6.22545776134535e-06 |
| ENSG00000 | ADAMTS13 | 9 | 1.33E+08 | 1.33E+08 | + | 5708 | 0.86 | 0.9 | 1.04 | 0.52 | 0.46 | 0.17 | 0.39505859 | -1.33986147 | 8.60150196104291e-07 | 6.65011145005203e-06 |
| ENSG00000 | - | 11 | 50184250 | 50208794 | + | 1432 | 1.95 | 1.34 | 2.73 | 4.52 | 4.85 | 4.19 | 2.16964159 | 1.117456742 | 8.65846783399728e-07 | 6.69155199685332e-06 |
| ENSG00000 | CARMN | 5 | 1.49E+08 | 1.49E+08 | + | 18220 | 0.1 | 0.14 | 0.12 | 0.03 | 0.02 | 0.04 | 0.23130433 | -2.112135835 | 1.04489467721369e-06 | 7.98836242075902e-06 |
| ENSG00000 | PARM1 | 4 | 74933095 | 75050115 | + | 5034 | 0.37 | 0.29 | 0.46 | 0.82 | 0.85 | 1.12 | 2.41454621 | 1.271752075 | 1.07820357604103e-06 | 8.23035628006751e-06 |
| ENSG00000 | RPL12P14 | 1 | 15792796 | 15793285 | + | 490 | 0.61 | 0.74 | 0.74 | 3.37 | 4.76 | 2.97 | 5.13613408 | 2.360682865 | 1.37775467438251e-06 | 1.03559715445034e-05 |
| ENSG00000 | JDP2-AS1 | 14 | 75423683 | 75427741 | - | 4059 | 0.2 | 0.17 | 0.14 | 0.5 | 0.78 | 0.57 | 3.56140717 | 1.832447385 | 1.49104884300133e-06 | 1.11464309184517e-05 |
| ENSG00000 | C2orf92 | 2 | 97664217 | 97703066 | + | 6503 | 0.41 | 0.49 | 0.38 | 0.14 | 0.12 | 0.17 | 0.31866659 | -1.649880342 | 1.54988646336797e-06 | 1.15558651383847e-05 |
| ENSG00000 | RENBP | X | 1.54E+08 | 1.54E+08 | - | 2047 | 0.36 | 0.61 | 0.6 | 1.43 | 1.47 | 1.85 | 2.90560928 | 1.538840716 | 1.5593935827519e-06 | 1.16223918264698e-05 |
| ENSG00000 | HES7 | 17 | 8120592 | 8124106 | - | 1880 | 1.11 | 0.91 | 0.9 | 2.77 | 2.25 | 2.11 | 2.3355593 | 1.223768074 | 1.74352598087095e-06 | 1.28932741277868e-05 |
| ENSG00000 | LINC02015 | 3 | 1.78E+08 | 1.78E+08 | + | 4384 | 0.37 | 0.25 | 0.26 | 0.85 | 0.89 | 0.7 | 2.66395546 | 1.413569959 | 1.87287022693411e-06 | 1.37933423935499e-05 |
| ENSG00000 | RNF150 | 4 | 1.41E+08 | 1.41E+08 | - | 11012 | 0.03 | 0.03 | 0.05 | 0.17 | 0.22 | 0.14 | 4.56746426 | 2.191393439 | 2.12618160582233e-06 | 1.55038948411357e-05 |
| ENSG00000 | EPHA3 | 3 | 89107621 | 89482134 | + | 6577 | 0.63 | 0.63 | 0.53 | 0.29 | 0.25 | 0.24 | 0.41927071 | -1.254046057 | 2.75485013577029e-06 | 1.97691068025233e-05 |
| ENSG00000 | RPL17P50 | 10 | 73005833 | 73006595 | - | 546 | 10.48 | 7.97 | 8.3 | 5.47 | 3.97 | 2.24 | 0.41704617 | -1.261720979 | 2.8297688179261e-06 | 2.02701559598201e-05 |
| ENSG00000 | LBP | 20 | 38346482 | 38377013 | + | 1825 | 0.85 | 1.05 | 0.73 | 0.14 | 0.12 | 0.22 | 0.17733947 | -2.495414399 | 2.92177348515801e-06 | 2.0899088400132e-05 |
| ENSG00000 | CCN5 | 20 | 44714844 | 44728509 | + | 4981 | 1.47 | 0.71 | 1.34 | 0.01 | 0.13 | 0.15 | 0.08232662 | -3.602497246 | 2.99766837825927e-06 | 2.13651024020379e-05 |
| ENSG00000 | TBC1D3L | 17 | 37978155 | 37989060 | - | 3505 | 1.45 | 1.68 | 1.79 | 0.76 | 0.78 | 1 | 0.49609056 | -1.011324579 | 3.00013483677831e-06 | 2.13750201466631e-05 |
| ENSG00000 | GGT5 | 22 | 24219654 | 24245108 | - | 6020 | 0.22 | 0.15 | 0.23 | 0.01 | 0.01 | 0.01 | 0.04639409 | -4.429915185 | 3.48634297379746e-06 | 2.45662402671731e-05 |

| | | | | | | | | | | | | | | | | |
|---|---|---|---|---|---|---|---|---|---|---|---|---|---|---|---|---|
| ENSG00000 | P2RY8 | X | 1462581 | 1537185 | - | 4528 | 0.32 | 0.43 | 0.22 | 0.84 | 0.8 | 0.91 | 2.53307965 | 1.340892441 | 3.49986243979609e-06 | 2.46440384615245e-05 |
| ENSG00000 | TNIP3 | 4 | 1.21E+08 | 1.21E+08 | - | 3749 | 0.6 | 0.59 | 0.54 | 1.3 | 1.19 | 1.35 | 2.13850464 | 1.096602338 | 3.56546717467087e-06 | 2.50704790552794e-05 |
| ENSG00000 | CCL26 | 7 | 75769533 | 75789896 | - | 703 | 1.7 | 2.06 | 1.98 | 4.88 | 5.85 | 4.39 | 2.53813813 | 1.343770587 | 3.7049944862249e-06 | 2.59597658064067e-05 |
| ENSG00000 | SLC38A4 | 12 | 46764761 | 46832408 | - | 4145 | 1.16 | 1.4 | 0.98 | 0.35 | 0.43 | 0.76 | 0.41949647 | -1.253269416 | 3.81739092751768e-06 | 2.67096476402847e-05 |
| ENSG00000 | CYP2E1 | 10 | 1.34E+08 | 1.34E+08 | + | 9622 | 0.46 | 0.51 | 0.43 | 0.16 | 0.18 | 0.28 | 0.43448676 | -1.20261588 | 3.98406372174002e-06 | 2.77878313247283e-05 |
| ENSG00000 | RAB38 | 11 | 88113251 | 88175443 | - | 1432 | 0.76 | 0.33 | 0.36 | 1.51 | 2.13 | 1.63 | 3.48638774 | 1.80173303 | 4.29857265636824e-06 | 2.98245349867001e-05 |
| ENSG00000 | SUSD2 | 22 | 24181487 | 24189106 | + | 5047 | 0.28 | 0.67 | 0.39 | 0.11 | 0.11 | 0.12 | 0.24516191 | -2.02819325 | 4.32063122918012e-06 | 2.99671266104802e-05 |
| ENSG00000 | RAB40B | 17 | 82654973 | 82698698 | - | 6164 | 0.55 | 0.6 | 0.69 | 0.27 | 0.26 | 0.27 | 0.42049008 | -1.249856335 | 4.43779096758502e-06 | 3.07047576167113e-05 |
| ENSG00000 | - | 7 | 94071759 | 94077157 | - | 446 | 1.34 | 1.97 | 0.92 | 3.7 | 6.85 | 5.49 | 3.65586483 | 1.870212729 | 4.47652052255023e-06 | 3.09404277340672e-05 |
| ENSG00000 | SLN | 11 | 1.08E+08 | 1.08E+08 | - | 906 | 3.13 | 2.34 | 2.62 | 0.28 | 0.8 | 1.22 | 0.27637444 | -1.85530391 | 4.54197563112855e-06 | 3.13601338281219e-05 |
| ENSG00000 | - | 7 | 1.56E+08 | 1.56E+08 | + | 5295 | 0.36 | 0.6 | 0.39 | 1.12 | 0.91 | 0.97 | 2.14653875 | 1.10201222 | 4.66349831136714e-06 | 3.21433843748824e-05 |
| ENSG00000 | GRAMD2B | 5 | 1.26E+08 | 1.26E+08 | + | 5051 | 0.28 | 0.22 | 0.13 | 0.79 | 0.47 | 0.63 | 2.91396052 | 1.542981331 | 5.17275981307247e-06 | 3.53958461400365e-05 |
| ENSG00000 | - | 7 | 1.44E+08 | 1.44E+08 | - | 7144 | 0.38 | 0.43 | 0.34 | 0.15 | 0.05 | 0.17 | 0.31300936 | -1.675722314 | 5.37212612524804e-06 | 3.66214357218229e-05 |
| ENSG00000 | ALPL | 1 | 21509397 | 21578410 | + | 2980 | 0.65 | 0.5 | 0.85 | 0.19 | 0.21 | 0.12 | 0.24703109 | -2.017235485 | 5.39992758766481e-06 | 3.67857348683504e-05 |
| ENSG00000 | SLC16A14 | 2 | 2.3E+08 | 2.3E+08 | - | 4932 | 0.24 | 0.26 | 0.22 | 0.8 | 0.52 | 0.69 | 2.65273681 | 1.407481545 | 5.83397095070246e-06 | 3.95124360881193e-05 |
| ENSG00000 | SLC9A9 | 3 | 1.43E+08 | 1.44E+08 | - | 4041 | 0.64 | 0.79 | 0.59 | 1.46 | 1.64 | 1.15 | 2.02373367 | 1.01701944 | 6.26041911284732e-06 | 4.21994691725319e-05 |
| ENSG00000 | DMBX1 | 1 | 46489836 | 46516216 | + | 5068 | 0.32 | 0.32 | 0.27 | 0.05 | 0.08 | 0.06 | 0.19487964 | -2.359344737 | 6.37043292870824e-06 | 4.28683786082448e-05 |
| ENSG00000 | MYBPH | 1 | 2.03E+08 | 2.03E+08 | - | 1818 | 1.07 | 1.2 | 1.53 | 0.38 | 0.15 | 0.54 | 0.27318184 | -1.872066503 | 6.54367316219537e-06 | 4.39004523718809e-05 |
| ENSG00000 | - | 3 | 1.78E+08 | 1.78E+08 | + | 5278 | 0.01 | 0 | 0 | 0.2 | 0.11 | 0.32 | 58.3688213 | 5.86712603 | 7.02334223483764e-06 | 4.68497686480196e-05 |
| ENSG00000 | PSG2 | 19 | 43064209 | 43083045 | - | 2833 | 0.35 | 0.57 | 0.4 | 0.07 | 0.06 | 0.04 | 0.1216614 | -3.039056591 | 7.50594070713603e-06 | 4.98017686657991e-05 |
| ENSG00000 | PELATON | 20 | 50247643 | 50283250 | + | 7595 | 0.17 | 0.15 | 0.15 | 0.51 | 0.37 | 0.39 | 2.58710618 | 1.371339265 | 8.88856915692074e-06 | 5.8333068543026e-05 |
| ENSG00000 | - | 1 | 1.71E+08 | 1.71E+08 | + | 2045 | 1.97 | 2 | 2.19 | 0.99 | 0.92 | 1.05 | 0.4637824 | -1.108480022 | 9.25319576265888e-06 | 6.05063458534929e-05 |
| ENSG00000 | HLA-C | 6 | 31268749 | 31272130 | - | 2507 | 20.48 | 15.89 | 27.42 | 10.59 | 10.7 | 11.72 | 0.49843541 | -1.004521539 | 9.3918619157969e-06 | 6.13727158053307e-05 |
| ENSG00000 | IFITM1 | 11 | 310041 | 315272 | + | 1666 | 1.19 | 1.18 | 0.84 | 0.27 | 0.23 | 0.31 | 0.24378283 | -2.036331548 | 1.04322257948985e-05 | 6.76376947934652e-05 |
| ENSG00000 | SUN3 | 7 | 47987148 | 48029119 | - | 1720 | 0.29 | 0.15 | 0.18 | 1.29 | 0.68 | 1.02 | 4.60638776 | 2.203635861 | 1.10287972032027e-05 | 7.12500430102943e-05 |
| ENSG00000 | NDRG2 | 14 | 21016763 | 21070872 | - | 7550 | 0.11 | 0.16 | 0.13 | 0.53 | 0.37 | 0.28 | 2.87228409 | 1.522198449 | 1.15671320287521e-05 | 7.44135944327841e-05 |
| ENSG00000 | LINC00972 | 7 | 85421122 | 85489293 | + | 813 | 0.12 | 0.19 | 0.06 | 1.48 | 1.57 | 1 | 10.3594968 | 3.372882029 | 1.22059481621058e-05 | 7.81691720462072e-05 |
| ENSG00000 | KCNMB4 | 12 | 70366290 | 70434292 | + | 4860 | 0.34 | 0.46 | 0.35 | 0.98 | 0.88 | 0.74 | 2.18439268 | 1.127232228 | 1.48371744845384e-05 | 9.37518953368436e-05 |
| ENSG00000 | CADM4 | 19 | 43622368 | 43639850 | - | 2257 | 1.9 | 2.29 | 2.1 | 1.46 | 0.86 | 0.7 | 0.45885077 | -1.123903056 | 1.51965341656489e-05 | 9.59006924417419e-05 |
| ENSG00000 | MANSC1 | 12 | 12326056 | 12350242 | - | 5716 | 0.17 | 0.2 | 0.19 | 0.43 | 0.66 | 0.46 | 2.66286622 | 1.412979952 | 1.57185946524072e-05 | 9.89127388174421e-05 |
| ENSG00000 | VNN1 | 6 | 1.33E+08 | 1.33E+08 | - | 3853 | 0.32 | 0.24 | 0.21 | 0.64 | 0.82 | 0.73 | 2.70542851 | 1.43585712 | 1.58200442019425e-05 | 9.9519639024241e-05 |
| ENSG00000 | NAT2 | 8 | 18391282 | 18401218 | + | 1285 | 0.04 | 0 | 0.08 | 0.69 | 0.65 | 1.04 | 19.2910895 | 4.26986272 | 1.66872925272307e-05 | 0.000104611 |
| ENSG00000 | - | 16 | 17227023 | 17228000 | - | 978 | 3.05 | 2.91 | 1.79 | 0.84 | 0.97 | 0.89 | 0.33543327 | -1.575902304 | 1.69564303332828e-05 | 0.000106198 |
| ENSG00000 | - | 15 | 99805793 | 99916818 | + | 7716 | 0.63 | 0.61 | 0.55 | 0.35 | 0.27 | 0.3 | 0.49355366 | -1.018721135 | 1.78655142278231e-05 | 0.00011161 |
| ENSG00000 | RPSAP44 | 6 | 54624605 | 54625488 | + | 884 | 0.23 | 0.18 | 0.23 | 1.44 | 1.95 | 0.92 | 6.53422556 | 2.708016257 | 1.87949447375482e-05 | 0.000117086 |
| ENSG00000 | LINC02381 | 12 | 54126019 | 54147485 | + | 4346 | 0.46 | 0.52 | 0.55 | 1.08 | 1.09 | 1.02 | 2.00835296 | 1.006012837 | 2.15309654191121e-05 | 0.000132675 |
| ENSG00000 | - | 9 | 5457477 | 5629748 | - | 3047 | 1.42 | 1.5 | 1.72 | 0.96 | 0.6 | 0.82 | 0.49191125 | -1.023530039 | 2.36160379520557e-05 | 0.000144716 |
| ENSG00000 | TMEM91 | 19 | 41350911 | 41384083 | + | 2487 | 1.48 | 2.21 | 2.15 | 0.59 | 1.21 | 1.01 | 0.46491707 | -1.104954691 | 2.75714737103991e-05 | 0.00016705 |
| ENSG00000 | PADI2 | 1 | 17066761 | 17119451 | - | 6000 | 0.29 | 0.37 | 0.27 | 0.12 | 0.06 | 0.11 | 0.28433476 | -1.814337596 | 3.33125488820108e-05 | 0.000199164 |
| ENSG00000 | CXCL1 | 4 | 73869393 | 73871308 | + | 1362 | 1.5 | 0.91 | 0.76 | 3.22 | 3.22 | 1.8 | 2.49067665 | 1.316537737 | 3.38502163914281e-05 | 0.000202075 |
| ENSG00000 | - | 8 | 1.35E+08 | 1.35E+08 | - | 2186 | 1.62 | 1.18 | 0.83 | 0.61 | 0.38 | 0.32 | 0.34473076 | -1.536458078 | 3.76046084486637e-05 | 0.00022189 |
| ENSG00000 | RPTN | 1 | 1.52E+08 | 1.52E+08 | - | 3569 | 0.43 | 0.25 | 0.1 | 1.46 | 1.49 | 0.96 | 4.80791291 | 2.265410762 | 3.76312208563055e-05 | 0.000221981 |
| ENSG00000 | KLHDC7B | 22 | 50545899 | 50551023 | + | 5125 | 0.07 | 0.04 | 0.04 | 0.42 | 0.17 | 0.23 | 5.27652192 | 2.399587274 | 4.06212780639028e-05 | 0.000237855 |
| ENSG00000 | MTFP1 | 22 | 30425623 | 30429054 | + | 1604 | 2.73 | 2.52 | 3.53 | 1.19 | 2.01 | 1.16 | 0.48005187 | -1.058737811 | 4.143238498467e-05 | 0.000242461 |
| ENSG00000 | RHPN1 | 8 | 1.43E+08 | 1.43E+08 | + | 4184 | 0.26 | 0.28 | 0.21 | 0.02 | 0.03 | 0.06 | 0.12520395 | -2.997648042 | 4.18582576343527e-05 | 0.000244593 |
| ENSG00000 | NR2F2-AS1 | 15 | 96110040 | 96327361 | - | 11966 | 0.25 | 0.35 | 0.31 | 0.15 | 0.13 | 0.15 | 0.45318842 | -1.141817106 | 4.32638384663164e-05 | 0.00025214 |
| ENSG00000 | - | 6 | 1.43E+08 | 1.43E+08 | + | 1138 | 4.07 | 2.96 | 3.12 | 2.01 | 1.42 | 1.49 | 0.46285655 | -1.11136296 | 4.65522367113813e-05 | 0.000269566 |
| ENSG00000 | - | 7 | 1.31E+08 | 1.31E+08 | + | 623 | 4.31 | 3.57 | 5.29 | 1.02 | 2.14 | 1.96 | 0.37726317 | -1.406356812 | 4.71674597934165e-05 | 0.000272891 |
| ENSG00000 | DNAH11 | 7 | 21543039 | 21901839 | + | 16147 | 0.03 | 0.04 | 0.07 | 0.14 | 0.11 | 0.18 | 2.98792694 | 1.57914487 | 4.78609173483031e-05 | 0.000276661 |
| ENSG00000 | SYT9-AS1 | 11 | 7418826 | 7513665 | - | 11515 | 0.04 | 0.04 | 0.04 | 0.17 | 0.11 | 0.18 | 3.64799559 | 1.867103984 | 4.93488543246935e-05 | 0.000284532 |
| ENSG00000 | LCNL1 | 9 | 1.37E+08 | 1.37E+08 | + | 4052 | 0.1 | 0.15 | 0.15 | 0 | 0 | 0 | 0.01915672 | -5.706005925 | 4.98499165975681e-05 | 0.000287074 |

| | | | | | | | | | | | | | | | | |
|---|---|---|---|---|---|---|---|---|---|---|---|---|---|---|---|---|
| ENSG00000 | - | 15 | 30344307 | 30348236 | - | 3930 | 0.2 | 0.16 | 0.09 | 0.6 | 0.61 | 0.36 | 3.30634589 | 1.725237658 | 5.25676785455648e-05 | 0.000301763 |
| ENSG00000 | - | 4 | 59152834 | 59176146 | + | 327 | 2.28 | 2.22 | 2.52 | 7.96 | 5.95 | 6.77 | 2.82858051 | 1.500078235 | 5.53243484162219e-05 | 0.000316674 |
| ENSG00000 | GDAP1L1 | 20 | 44247099 | 44280947 | + | 3404 | 0.16 | 0.08 | 0.09 | 0.71 | 0.57 | 0.22 | 4.37845215 | 2.130420946 | 5.70877921885121e-05 | 0.000326111 |
| ENSG00000 | FRAS1 | 4 | 78057323 | 78544269 | + | 20323 | 0.04 | 0.06 | 0.03 | 0.14 | 0.14 | 0.1 | 2.69219339 | 1.428782048 | 7.00829315947342e-05 | 0.000392342 |
| ENSG00000 | NECAB2 | 16 | 83968244 | 84002776 | + | 2908 | 1.15 | 1.03 | 1.33 | 0.59 | 0.53 | 0.56 | 0.46187632 | -1.114421518 | 7.18471693688782e-05 | 0.000401766 |
| ENSG00000 | - | 16 | 67261108 | 67263784 | + | 2677 | 1.19 | 1.37 | 1.48 | 0.69 | 0.66 | 0.65 | 0.4769959 | -1.067951233 | 7.30619627363289e-05 | 0.000407871 |
| ENSG00000 | MYO3B | 2 | 1.7E+08 | 1.71E+08 | + | 8236 | 0.25 | 0.2 | 0.32 | 0.77 | 0.45 | 0.48 | 2.10226699 | 1.071945906 | 7.32854495972566e-05 | 0.000409004 |
| ENSG00000 | BRSK2 | 11 | 1389899 | 1462689 | + | 6903 | 0.2 | 0.08 | 0.07 | 0 | 0.01 | 0 | 0.02506294 | -5.318300344 | 7.72221777463272e-05 | 0.000429048 |
| ENSG00000 | DRICH1 | 22 | 23608452 | 23632321 | - | 1302 | 0.88 | 0.8 | 1.27 | 0.29 | 0.21 | 0.13 | 0.20812166 | -2.264500999 | 8.456438562434e-05 | 0.00046542 |
| ENSG00000 | PI4KAP1 | 22 | 18533646 | 18577968 | - | 4812 | 0.62 | 0.6 | 0.81 | 0.34 | 0.33 | 0.28 | 0.45166752 | -1.146666933 | 8.4892369130401e-05 | 0.000466838 |
| ENSG00000 | DACH1 | 13 | 71437966 | 71867204 | - | 5401 | 0.15 | 0.1 | 0.1 | 0.42 | 0.27 | 0.43 | 3.08818564 | 1.626759479 | 8.52990069896459e-05 | 0.000468944 |
| ENSG00000 | CYSLTR1 | X | 78271468 | 78327691 | - | 5567 | 0.4 | 0.33 | 0.31 | 0.13 | 0.15 | 0.08 | 0.3312488 | -1.594012879 | 0.000100773 | 0.000546013 |
| ENSG00000 | C1QTNF3 | 5 | 34017858 | 34043213 | - | 4547 | 0.42 | 0.32 | 0.18 | 0.1 | 0.04 | 0.08 | 0.21897381 | -2.191169795 | 0.00010315 | 0.000557982 |
| ENSG00000 | TPRXL | 3 | 13937273 | 14082811 | + | 3748 | 0.24 | 0.03 | 0.16 | 0 | 0 | 0 | 0.01933607 | -5.692561224 | 0.000109077 | 0.000588122 |
| ENSG00000 | PPIL6 | 6 | 1.09E+08 | 1.09E+08 | - | 4481 | 0.28 | 0.29 | 0.43 | 0.69 | 0.89 | 0.68 | 2.20092178 | 1.138107873 | 0.000109858 | 0.000592175 |
| ENSG00000 | SPTBN1-AS2 | 2 | 54661011 | 54680045 | - | 1236 | 1.33 | 0.84 | 1.29 | 0.36 | 0.27 | 0.28 | 0.2524865 | -1.985721856 | 0.000113677 | 0.000609785 |
| ENSG00000 | SRD5A2 | 2 | 31522480 | 31580938 | - | 4519 | 0.04 | 0.08 | 0.02 | 0.24 | 0.28 | 0.26 | 5.10953682 | 2.353192517 | 0.000115587 | 0.000618861 |
| ENSG00000 | CCDC69 | 5 | 1.51E+08 | 1.51E+08 | - | 4941 | 0.35 | 0.31 | 0.19 | 0.6 | 0.67 | 0.71 | 2.23417553 | 1.159742536 | 0.000122444 | 0.00065206 |
| ENSG00000 | FER1L6 | 8 | 1.24E+08 | 1.24E+08 | + | 6051 | 0.08 | 0.11 | 0.2 | 0.37 | 0.46 | 0.31 | 2.81370781 | 1.492472521 | 0.000125553 | 0.000666119 |
| ENSG00000 | PLAAT4 | 11 | 63536808 | 63546462 | + | 1300 | 2.64 | 2.67 | 3.01 | 1.32 | 1.62 | 1.3 | 0.49167596 | -1.024220271 | 0.0001297 | 0.000686655 |
| ENSG00000 | - | 17 | 82249067 | 82251844 | + | 2778 | 0.68 | 0.47 | 0.56 | 0.25 | 0.1 | 0.15 | 0.27769993 | -1.848401268 | 0.000130588 | 0.000690413 |
| ENSG00000 | LINC01141 | 1 | 20360579 | 20431234 | - | 4734 | 0.24 | 0.31 | 0.23 | 0.03 | 0.07 | 0.07 | 0.21463655 | -2.220032331 | 0.000132484 | 0.000699348 |
| ENSG00000 | MSH5 | 6 | 31739677 | 31762676 | + | 6284 | 0.56 | 0.62 | 0.51 | 0.31 | 0.25 | 0.3 | 0.48734487 | -1.036985048 | 0.000138535 | 0.000728966 |
| ENSG00000 | CACNG7 | 19 | 53909278 | 53943950 | + | 2866 | 0.57 | 0.31 | 0.61 | 0.98 | 1.16 | 1.32 | 2.23370538 | 1.159438912 | 0.000157855 | 0.000822357 |
| ENSG00000 | DAZL | 3 | 16586792 | 16670306 | - | 3555 | 0.2 | 0.07 | 0.04 | 0.32 | 0.47 | 0.51 | 3.98212595 | 1.993538852 | 0.000158503 | 0.000825304 |
| ENSG00000 | MCHR1 | 22 | 40679273 | 40682812 | + | 2436 | 0.51 | 0.68 | 0.68 | 0.21 | 0.16 | 0.19 | 0.28754805 | -1.798125059 | 0.000160827 | 0.000836748 |
| ENSG00000 | SMKR1 | 7 | 1.3E+08 | 1.3E+08 | + | 969 | 1.18 | 1.92 | 2.55 | 3.67 | 4.3 | 4.21 | 2.07951395 | 1.056246362 | 0.000161034 | 0.000837383 |
| ENSG00000 | CDH23 | 10 | 71396920 | 71815947 | + | 18592 | 0.19 | 0.18 | 0.19 | 0.08 | 0.1 | 0.1 | 0.49237768 | -1.022162716 | 0.000163397 | 0.000847626 |
| ENSG00000 | HAS3 | 16 | 69105653 | 69118719 | + | 5200 | 0.52 | 0.56 | 0.48 | 0.23 | 0.25 | 0.22 | 0.43581412 | -1.198215145 | 0.000164149 | 0.000851135 |
| ENSG00000 | BCL2A1 | 15 | 79960892 | 79971196 | - | 995 | 0.15 | 0.36 | 0.16 | 1.4 | 0.95 | 1.11 | 4.98180559 | 2.316668722 | 0.000168227 | 0.00087024 |
| ENSG00000 | ELMO1 | 7 | 36852906 | 37449223 | - | 10999 | 0.29 | 0.18 | 0.21 | 0.08 | 0.09 | 0.11 | 0.39897108 | -1.325643919 | 0.000173125 | 0.000893481 |
| ENSG00000 | FHIT | 3 | 59747277 | 61251459 | - | 7472 | 0.52 | 0.43 | 0.5 | 0.23 | 0.24 | 0.28 | 0.49824085 | -1.00508479 | 0.000175741 | 0.000906281 |
| ENSG00000 | MIAT | 22 | 26646411 | 26676475 | + | 15892 | 0.18 | 0.11 | 0.16 | 0.04 | 0.07 | 0.07 | 0.38269894 | -1.385718187 | 0.000176388 | 0.000909377 |
| ENSG00000 | HOXD10 | 2 | 1.76E+08 | 1.76E+08 | + | 2488 | 0.36 | 0.44 | 0.62 | 1.1 | 1.16 | 1.1 | 2.2839547 | 1.191534039 | 0.000214708 | 0.001089153 |
| ENSG00000 | SYN3 | 22 | 32507820 | 33058381 | - | 10185 | 0.05 | 0.02 | 0.07 | 0 | 0 | 0 | 0.02281084 | -5.45413684 | 0.000217475 | 0.001102344 |
| ENSG00000 | CCL20 | 2 | 2.28E+08 | 2.28E+08 | + | 1594 | 0.37 | 0.16 | 0.16 | 0.56 | 1.25 | 0.99 | 3.85241059 | 1.945761476 | 0.000231435 | 0.001167745 |
| ENSG00000 | TLE2 | 19 | 2997639 | 3047635 | - | 4150 | 0.22 | 0.34 | 0.12 | 0.03 | 0.04 | 0.03 | 0.14091595 | -2.827093207 | 0.000235721 | 0.001187864 |
| ENSG00000 | DENND3-AS1 | 8 | 1.41E+08 | 1.41E+08 | - | 2828 | 0.97 | 1.43 | 1.09 | 0.49 | 0.65 | 0.58 | 0.47583249 | -1.071474321 | 0.000237548 | 0.001195253 |
| ENSG00000 | UBAP1L | 15 | 65092760 | 65115200 | - | 8549 | 0.28 | 0.29 | 0.31 | 0.15 | 0.13 | 0.12 | 0.43154678 | -1.212411142 | 0.000243111 | 0.001219851 |
| ENSG00000 | CISH | 3 | 50606489 | 50611774 | - | 5056 | 0.32 | 0.25 | 0.25 | 0.04 | 0.09 | 0.1 | 0.26988065 | -1.889606531 | 0.000243417 | 0.001221079 |
| ENSG00000 | IGF2BP1 | 17 | 48997385 | 49056145 | + | 9631 | 0.05 | 0.06 | 0.09 | 0.01 | 0 | 0 | 0.03145815 | -4.990422467 | 0.000244183 | 0.00122461 |
| ENSG00000 | ABCD2 | 12 | 39550033 | 39619803 | - | 6290 | 0.03 | 0.02 | 0.04 | 0.22 | 0.19 | 0.09 | 5.47914089 | 2.453949701 | 0.000246317 | 0.00123438 |
| ENSG00000 | - | 8 | 53177571 | 53242683 | + | 842 | 0 | 0.18 | 0 | 0.91 | 0.66 | 1.18 | 14.4636293 | 3.854357705 | 0.000249314 | 0.00124751 |
| ENSG00000 | CCDC190 | 1 | 1.63E+08 | 1.63E+08 | - | 5861 | 0.23 | 0.27 | 0.12 | 0.02 | 0.06 | 0.06 | 0.2176763 | -2.199743788 | 0.000252249 | 0.001260294 |
| ENSG00000 | - | X | 1.49E+08 | 1.5E+08 | - | 9702 | 0.14 | 0.18 | 0.15 | 0.05 | 0.06 | 0.04 | 0.30821329 | -1.697999039 | 0.000256284 | 0.001278845 |
| ENSG00000 | - | 12 | 1.19E+08 | 1.2E+08 | - | 1469 | 2.95 | 3.38 | 1.47 | 1.38 | 1.17 | 1.19 | 0.46034476 | -1.119213371 | 0.00025816 | 0.001287241 |
| ENSG00000 | AKR7L | 1 | 19265982 | 19274194 | - | 2768 | 0.95 | 0.6 | 0.63 | 0.32 | 0.28 | 0.23 | 0.36565085 | -1.45146139 | 0.000263902 | 0.001313564 |
| ENSG00000 | - | 7 | 56288230 | 56291425 | + | 2884 | 0.55 | 0.34 | 0.43 | 0.97 | 0.96 | 1.09 | 2.19665726 | 1.135309785 | 0.000275455 | 0.001365828 |
| ENSG00000 | KLRC2 | 12 | 10426854 | 10442300 | - | 1506 | 0.89 | 0.79 | 0.92 | 2.45 | 1.96 | 1.55 | 2.18906154 | 1.130312514 | 0.000276153 | 0.001368721 |
| ENSG00000 | VAC14-AS1 | 16 | 70755035 | 70773251 | + | 9145 | 0.22 | 0.24 | 0.12 | 0.03 | 0.11 | 0.03 | 0.28404939 | -1.815786279 | 0.000286379 | 0.001416932 |
| ENSG00000 | CXXC4 | 4 | 1.04E+08 | 1.04E+08 | - | 8112 | 0.06 | 0.04 | 0.03 | 0.18 | 0.17 | 0.14 | 3.73150939 | 1.899759316 | 0.000300696 | 0.001481868 |

| | | | | | | | | | | | | | | | | |
|---|---|---|---|---|---|---|---|---|---|---|---|---|---|---|---|---|
| ENSG00000 | ACSM3 | 16 | 20610243 | 20797581 | + | 7510 | 0.11 | 0.11 | 0.12 | 0.35 | 0.17 | 0.41 | 2.67813644 | 1.421229462 | 0.000309015 | 0.001519475 |
| ENSG00000 | TNXB | 6 | 32041153 | 32115334 | - | 15909 | 0.01 | 0.01 | 0.05 | 0.09 | 0.07 | 0.11 | 4.22759465 | 2.079837056 | 0.000313455 | 0.001539026 |
| ENSG00000 | H2BC11 | 6 | 27125897 | 27132795 | - | 1046 | 1.33 | 1.58 | 1.13 | 3.04 | 3.19 | 2.62 | 2.10020506 | 1.070530197 | 0.000317088 | 0.001554176 |
| ENSG00000 | - | 6 | 1528364 | 1528911 | - | 548 | 2.09 | 0.95 | 1.13 | 0.12 | 0 | 0.21 | 0.07475763 | -3.741635263 | 0.000318833 | 0.001561576 |
| ENSG00000 | PCDHGA6 | 5 | 1.41E+08 | 1.42E+08 | + | 7929 | 0.16 | 0.12 | 0.16 | 0.03 | 0.05 | 0.03 | 0.24095556 | -2.053160982 | 0.000321212 | 0.001572063 |
| ENSG00000 | - | 2 | 37208875 | 37212677 | + | 3803 | 0.47 | 0.5 | 0.26 | 0.18 | 0.13 | 0.06 | 0.29125939 | -1.779623516 | 0.00034069 | 0.001658006 |
| ENSG00000 | SERPINB4 | 18 | 63637259 | 63644256 | - | 1971 | 0.33 | 0.6 | 0.71 | 1.1 | 1.44 | 1.45 | 2.35030251 | 1.232846461 | 0.000360049 | 0.001745388 |
| ENSG00000 | NEIL1 | 15 | 75346955 | 75357115 | + | 6939 | 0.38 | 0.31 | 0.32 | 0.12 | 0.14 | 0.18 | 0.42152341 | -1.246315344 | 0.000365352 | 0.001768509 |
| ENSG00000 | SEC31B | 10 | 1E+08 | 1.01E+08 | - | 7934 | 0.51 | 0.39 | 0.34 | 0.2 | 0.15 | 0.26 | 0.47739358 | -1.06674893 | 0.000367128 | 0.001775805 |
| ENSG00000 | FCER1G | 1 | 1.61E+08 | 1.61E+08 | + | 964 | 0.26 | 0.16 | 0.21 | 0.99 | 1.15 | 1.03 | 4.80948299 | 2.265881816 | 0.000367988 | 0.001779103 |
| ENSG00000 | RPL21P89 | 10 | 49815096 | 49815562 | + | 467 | 1.17 | 1.33 | 1.32 | 0.14 | 0 | 0 | 0.03416492 | -4.871340288 | 0.000376566 | 0.0018166 |
| ENSG00000 | - | 11 | 78223815 | 78225909 | + | 1753 | 0.88 | 1.03 | 0.38 | 0.18 | 0.16 | 0.27 | 0.25341428 | -1.980430288 | 0.000391997 | 0.001880091 |
| ENSG00000 | GABRR2 | 6 | 89254464 | 89315299 | - | 4974 | 0.15 | 0.29 | 0.29 | 0.11 | 0.02 | 0.04 | 0.22192901 | -2.17182981 | 0.000400536 | 0.001917807 |
| ENSG00000 | LINC02232 | 4 | 64885649 | 65024485 | - | 6564 | 0 | 0 | 0 | 0.06 | 0.09 | 0.04 | 41.8846674 | 5.388350311 | 0.000401456 | 0.001921752 |
| ENSG00000 | CROCCP3 | 1 | 16467436 | 16499257 | - | 6905 | 0.45 | 0.46 | 0.43 | 0.3 | 0.11 | 0.23 | 0.45931333 | -1.122449448 | 0.00043004 | 0.002046755 |
| ENSG00000 | PTPRF | 1 | 43525187 | 43623666 | + | 10712 | 0.12 | 0.13 | 0.15 | 0.02 | 0.05 | 0.05 | 0.30227923 | -1.726046227 | 0.000430185 | 0.002046957 |
| ENSG00000 | NR4A3 | 9 | 99821855 | 99866891 | + | 6314 | 0.2 | 0.24 | 0.24 | 0.35 | 0.63 | 0.51 | 2.12358213 | 1.086499905 | 0.000434796 | 0.002065438 |
| ENSG00000 | IGSF22 | 11 | 18704312 | 18726230 | - | 4834 | 0.12 | 0.02 | 0.11 | 0.25 | 0.3 | 0.37 | 3.52108289 | 1.816019191 | 0.000447786 | 0.002120556 |
| ENSG00000 | - | 12 | 55761550 | 55762628 | - | 529 | 0.56 | 1.17 | 1.66 | 0.12 | 0 | 0 | 0.03407916 | -4.874966463 | 0.000448145 | 0.002121755 |
| ENSG00000 | DNM1P35 | 15 | 75727670 | 75738629 | - | 3714 | 0.5 | 0.4 | 0.4 | 0.07 | 0.22 | 0.14 | 0.32450887 | -1.623670201 | 0.000458924 | 0.002168657 |
| ENSG00000 | - | 8 | 1.24E+08 | 1.24E+08 | + | 2464 | 0.67 | 0.59 | 0.4 | 1.44 | 1.24 | 0.99 | 2.13301102 | 1.092891421 | 0.000459342 | 0.002169094 |
| ENSG00000 | - | 8 | 87540835 | 87755718 | - | 2921 | 0 | 0 | 0 | 0.13 | 0.15 | 0.12 | 40.1684245 | 5.327989973 | 0.00046073 | 0.002174608 |
| ENSG00000 | - | 8 | 1.03E+08 | 1.03E+08 | + | 2556 | 0.66 | 0.53 | 0.4 | 0.15 | 0.15 | 0.18 | 0.29210553 | -1.77543844 | 0.000467724 | 0.002202911 |
| ENSG00000 | NPIPB12 | 16 | 29484690 | 29505999 | - | 3560 | 0.48 | 0.32 | 0.46 | 0.09 | 0.17 | 0.15 | 0.31444843 | -1.669104677 | 0.000468006 | 0.002203716 |
| ENSG00000 | NLRX1 | 11 | 1.19E+08 | 1.19E+08 | + | 5320 | 0.29 | 0.43 | 0.35 | 0.12 | 0.14 | 0.16 | 0.37944477 | -1.3980382 | 0.000484775 | 0.002274064 |
| ENSG00000 | - | 1 | 1059687 | 1069355 | + | 2658 | 0.41 | 0.23 | 0.39 | 0.76 | 0.88 | 0.92 | 2.38617635 | 1.254700666 | 0.000510547 | 0.002383837 |
| ENSG00000 | - | 18 | 12040101 | 12040300 | - | 200 | 2.99 | 3.11 | 6.96 | 0.64 | 0.56 | 0.87 | 0.1525571 | -2.712578741 | 0.000516698 | 0.002406779 |
| ENSG00000 | FOXS1 | 20 | 31844303 | 31845604 | - | 1302 | 1.3 | 1.19 | 1.62 | 0.29 | 0.17 | 0.9 | 0.3184599 | -1.650816395 | 0.000555512 | 0.00257611 |
| ENSG00000 | LRRC2 | 3 | 46515385 | 46606948 | - | 5774 | 0 | 0 | 0 | 0.08 | 0.1 | 0.03 | 40.0973539 | 5.325435128 | 0.000566001 | 0.002619861 |
| ENSG00000 | ZNF691-DT | 1 | 42832522 | 42846427 | - | 3784 | 0.37 | 0.31 | 0.49 | 0.84 | 0.81 | 0.82 | 2.02026858 | 1.014547103 | 0.000568336 | 0.002629446 |
| ENSG00000 | NCF2 | 1 | 1.84E+08 | 1.84E+08 | - | 4319 | 0.08 | 0.02 | 0.07 | 0.16 | 0.37 | 0.26 | 4.32438053 | 2.112493481 | 0.00059012 | 0.002721996 |
| ENSG00000 | SLITRK3 | 3 | 1.65E+08 | 1.65E+08 | - | 5274 | 0.01 | 0 | 0.01 | 0.12 | 0.14 | 0.11 | 18.3228184 | 4.19556953 | 0.00060509 | 0.002784085 |
| ENSG00000 | COL5A3 | 19 | 9959561 | 10010504 | - | 6783 | 0.28 | 0.4 | 0.21 | 0.13 | 0.11 | 0.11 | 0.38621011 | -1.372542164 | 0.000612366 | 0.002812466 |
| ENSG00000 | TMEM178B | 7 | 1.41E+08 | 1.41E+08 | + | 10786 | 0.06 | 0.06 | 0.02 | 0.14 | 0.15 | 0.15 | 3.06142539 | 1.614203526 | 0.000625524 | 0.002867347 |
| ENSG00000 | RFPL4A | 19 | 55759097 | 55763421 | + | 1198 | 0 | 0 | 0 | 0.16 | 0.23 | 0.58 | 40.0789127 | 5.324771466 | 0.000627995 | 0.002878011 |
| ENSG00000 | IL1RL1 | 2 | 1.02E+08 | 1.02E+08 | + | 5746 | 0.06 | 0.07 | 0.12 | 0.28 | 0.2 | 0.31 | 3.06766718 | 1.61714197 | 0.000650098 | 0.002969721 |
| ENSG00000 | LINC01723 | 20 | 12950288 | 13008417 | + | 2900 | 0.03 | 0.05 | 0.05 | 0.33 | 0.23 | 0.3 | 5.86312631 | 2.551670136 | 0.000663095 | 0.00302215 |
| ENSG00000 | ROR1 | 1 | 63774017 | 64181498 | + | 6718 | 0.48 | 0.37 | 0.32 | 0.18 | 0.23 | 0.15 | 0.45820699 | -1.125928613 | 0.000671115 | 0.003054503 |
| ENSG00000 | CALCRL | 2 | 1.87E+08 | 1.87E+08 | - | 7309 | 0.15 | 0.09 | 0.14 | 0.33 | 0.24 | 0.37 | 2.35711204 | 1.237020333 | 0.000673718 | 0.003062843 |
| ENSG00000 | ARHGAP15 | 2 | 1.43E+08 | 1.44E+08 | + | 6127 | 0.02 | 0.03 | 0.08 | 0.29 | 0.13 | 0.17 | 4.22652545 | 2.079472137 | 0.000675838 | 0.00307178 |
| ENSG00000 | RPS6KL1 | 14 | 74903951 | 74923302 | - | 7526 | 0.03 | 0 | 0.04 | 0.15 | 0.17 | 0.07 | 5.55996709 | 2.475076342 | 0.000678372 | 0.00308189 |
| ENSG00000 | GPSM3 | 6 | 32190766 | 32195523 | - | 1833 | 0.05 | 0.17 | 0.2 | 0.55 | 0.52 | 0.73 | 4.14397516 | 2.051015355 | 0.000679127 | 0.003084613 |
| ENSG00000 | LINC01561 | 10 | 1.21E+08 | 1.21E+08 | + | 2176 | 0.59 | 0.69 | 0.92 | 0.09 | 0.43 | 0.19 | 0.3141514 | -1.670468092 | 0.000683442 | 0.003101379 |
| ENSG00000 | MYO7B | 2 | 1.28E+08 | 1.28E+08 | + | 8910 | 0.19 | 0.09 | 0.13 | 0.05 | 0.01 | 0.05 | 0.24733437 | -2.015465368 | 0.00068963 | 0.003126604 |
| ENSG00000 | CNKSR2 | X | 21372801 | 21654695 | + | 31865 | 0.02 | 0.03 | 0.02 | 0.07 | 0.05 | 0.08 | 2.45786339 | 1.297404734 | 0.000691017 | 0.003132182 |
| ENSG00000 | CDKL2 | 4 | 75576496 | 75630716 | - | 5290 | 0.22 | 0.23 | 0.24 | 0.37 | 0.44 | 0.78 | 2.24827858 | 1.168820808 | 0.000694062 | 0.003143834 |
| ENSG00000 | CDNF | 10 | 14819245 | 14838575 | - | 1703 | 0.15 | 0.27 | 0.06 | 0.71 | 0.72 | 0.58 | 4.02246213 | 2.008078838 | 0.000696416 | 0.003153058 |
| ENSG00000 | LINC02104 | 5 | 39520398 | 39524726 | + | 650 | 0 | 0.88 | 0.24 | 1.86 | 1.71 | 1.79 | 4.66184921 | 2.220902341 | 0.000717273 | 0.00323534 |
| ENSG00000 | REC8 | 14 | 24171853 | 24180257 | + | 3843 | 0.62 | 0.63 | 0.42 | 0.15 | 0.22 | 0.33 | 0.40448725 | -1.305833853 | 0.000735586 | 0.003304819 |
| ENSG00000 | FLRT2-AS1 | 14 | 85517967 | 85531660 | - | 6444 | 0.29 | 0.15 | 0.3 | 0.66 | 0.42 | 0.5 | 2.04062357 | 1.029010078 | 0.000763734 | 0.003416615 |
| ENSG00000 | POU2F3 | 11 | 1.2E+08 | 1.2E+08 | + | 6700 | 0.02 | 0 | 0.03 | 0.08 | 0.14 | 0.15 | 6.62186084 | 2.727236692 | 0.000784401 | 0.003501978 |

| | | | | | | | | | | | | | | | | |
|---|---|---|---|---|---|---|---|---|---|---|---|---|---|---|---|---|
| ENSG00000 | S1PR1 | 1 | 1.01E+08 | 1.01E+08 | + | 5223 | 0.2 | 0.25 | 0.14 | 0.45 | 0.47 | 0.45 | 2.2391423 | 1.162946214 | 0.000800693 | 0.00356591 |
| ENSG00000 | CHRNA10 | 11 | 3665587 | 3671384 | - | 2703 | 0.64 | 0.44 | 0.61 | 0.21 | 0.16 | 0.24 | 0.34705756 | -1.526753152 | 0.000807277 | 0.00359362 |
| ENSG00000 | ACAD9-DT | 3 | 1.29E+08 | 1.29E+08 | - | 3591 | 0.79 | 0.62 | 0.79 | 0.32 | 0.46 | 0.29 | 0.47164781 | -1.084218112 | 0.000825511 | 0.0036674 |
| ENSG00000 | - | 6 | 45421079 | 45422005 | - | 927 | 2.31 | 3.3 | 2.72 | 1.58 | 1.02 | 1.45 | 0.46518635 | -1.104119344 | 0.000826203 | 0.003669655 |
| ENSG00000 | RAPGEF5 | 7 | 22118236 | 22357154 | - | 10257 | 0.08 | 0.07 | 0.08 | 0.17 | 0.2 | 0.2 | 2.41230646 | 1.270413202 | 0.000858242 | 0.003800077 |
| ENSG00000 | - | 12 | 1.04E+08 | 1.04E+08 | - | 701 | 0.43 | 0.66 | 0.37 | 1.63 | 1.82 | 1.75 | 3.43610009 | 1.780772062 | 0.000873278 | 0.003858063 |
| ENSG00000 | - | 7 | 16695871 | 16719898 | - | 648 | 0.46 | 0.16 | 0.4 | 0.98 | 1.89 | 1.8 | 4.41231458 | 2.141535653 | 0.000908009 | 0.004000835 |
| ENSG00000 | DUSP5-DT | 10 | 1.1E+08 | 1.1E+08 | - | 3430 | 0.23 | 0.12 | 0.15 | 0.37 | 0.49 | 0.59 | 2.77335594 | 1.471632787 | 0.000951794 | 0.004167899 |
| ENSG00000 | - | 5 | 1.33E+08 | 1.33E+08 | + | 2034 | 0.44 | 0.23 | 0.61 | 0.62 | 1.31 | 1.43 | 2.5480779 | 1.349409382 | 0.000978481 | 0.004276283 |
| ENSG00000 | ACOT1 | 14 | 73537143 | 73543796 | + | 1686 | 0.65 | 0.58 | 0.64 | 1.21 | 1.55 | 1.45 | 2.16208039 | 1.112420169 | 0.001033862 | 0.00448872 |
| ENSG00000 | ZNF652-AS1 | 17 | 49361150 | 49369998 | + | 2377 | 0.31 | 0.5 | 0.37 | 0.05 | 0.14 | 0.07 | 0.22013973 | -2.183508544 | 0.001041277 | 0.004517954 |
| ENSG00000 | SPATA6L | 9 | 4553386 | 4666674 | - | 9565 | 0.05 | 0.1 | 0.08 | 0.21 | 0.19 | 0.18 | 2.5203389 | 1.333617742 | 0.001089576 | 0.004698813 |
| ENSG00000 | GRM1 | 6 | 1.46E+08 | 1.46E+08 | + | 7408 | 0.03 | 0.03 | 0.03 | 0.15 | 0.13 | 0.14 | 3.86082372 | 1.948908683 | 0.00109477 | 0.004719165 |
| ENSG00000 | COL21A1 | 6 | 56056590 | 56394094 | - | 6912 | 0.16 | 0.19 | 0.24 | 0.03 | 0.12 | 0.02 | 0.27628001 | -1.855796921 | 0.001099646 | 0.004736075 |
| ENSG00000 | - | 20 | 311731 | 313237 | + | 629 | 2.37 | 2.96 | 3.36 | 1.11 | 1.68 | 0.74 | 0.39240693 | -1.349577561 | 0.001104932 | 0.00475678 |
| ENSG00000 | CDH3 | 16 | 68636189 | 68727468 | + | 6392 | 0.31 | 0.46 | 0.32 | 0.08 | 0.24 | 0.15 | 0.42411695 | -1.237465946 | 0.001115768 | 0.004794089 |
| ENSG00000 | FEZF1 | 7 | 1.22E+08 | 1.22E+08 | - | 2942 | 0.41 | 0.39 | 0.19 | 0.8 | 0.7 | 0.83 | 2.26757601 | 1.181150912 | 0.001145081 | 0.004911547 |
| ENSG00000 | STARD4-AS1 | 5 | 1.12E+08 | 1.12E+08 | + | 9263 | 0.87 | 0.73 | 0.67 | 0.2 | 0.5 | 0.33 | 0.43486594 | -1.201357365 | 0.001167661 | 0.004998694 |
| ENSG00000 | LMNTD1 | 12 | 25409307 | 25648579 | - | 3083 | 0 | 0 | 0 | 0.04 | 0.14 | 0.15 | 35.6351462 | 5.155228938 | 0.001287334 | 0.005463958 |
| ENSG00000 | LINC00571 | 13 | 38050817 | 38350259 | - | 1625 | 0.09 | 0.03 | 0.06 | 0.35 | 0.48 | 0.47 | 6.64833484 | 2.732993044 | 0.001298056 | 0.005507114 |
| ENSG00000 | - | 12 | 4909867 | 5026012 | + | 9655 | 0.02 | 0.08 | 0.02 | 0 | 0 | 0 | 0.02905839 | -5.104901393 | 0.001319852 | 0.005591237 |
| ENSG00000 | - | 8 | 1.44E+08 | 1.45E+08 | - | 4894 | 0.31 | 0.23 | 0.33 | 0.06 | 0.12 | 0.12 | 0.3465407 | -1.528903313 | 0.001357181 | 0.005737148 |
| ENSG00000 | TRAC | 14 | 22547506 | 22552156 | + | 978 | 2.49 | 2.22 | 1.58 | 0.45 | 0.74 | 1.43 | 0.40288085 | -1.311574848 | 0.001371258 | 0.00579296 |
| ENSG00000 | - | 6 | 1.43E+08 | 1.43E+08 | - | 2556 | 0 | 0.02 | 0.08 | 0.4 | 0.11 | 0.34 | 8.13487622 | 3.024120393 | 0.001428821 | 0.00601824 |
| ENSG00000 | PCAT6 | 1 | 2.03E+08 | 2.03E+08 | + | 1624 | 0.98 | 1.5 | 1.24 | 0.66 | 0.44 | 0.57 | 0.4343761 | -1.202983357 | 0.00148058 | 0.006206017 |
| ENSG00000 | LINC00310 | 21 | 34157724 | 34190244 | + | 3280 | 0.24 | 0.33 | 0.36 | 0.08 | 0.14 | 0.04 | 0.25742933 | -1.957751681 | 0.001483255 | 0.006215915 |
| ENSG00000 | TNFSF15 | 9 | 1.15E+08 | 1.15E+08 | - | 7785 | 0.01 | 0.01 | 0.01 | 0.09 | 0.09 | 0.07 | 7.34930186 | 2.87760721 | 0.001511442 | 0.006322455 |
| ENSG00000 | MMP10 | 11 | 1.03E+08 | 1.03E+08 | - | 1759 | 0 | 0.03 | 0 | 0.51 | 0.09 | 0.23 | 25.5162298 | 4.673343274 | 0.00151403 | 0.006331544 |
| ENSG00000 | LINC01252 | 12 | 11548030 | 11590369 | + | 4456 | 0.36 | 0.41 | 0.69 | 0.21 | 0.1 | 0.3 | 0.40491209 | -1.304319381 | 0.001516339 | 0.006339866 |
| ENSG00000 | LINC02593 | 1 | 916865 | 921016 | - | 4152 | 0.7 | 0.37 | 0.69 | 0.34 | 0.23 | 0.28 | 0.45851009 | -1.124974615 | 0.001523534 | 0.006365933 |
| ENSG00000 | GABRG1 | 4 | 46035769 | 46124054 | - | 6758 | 0 | 0 | 0.03 | 0.12 | 0.07 | 0.1 | 9.25654024 | 3.210473066 | 0.001547818 | 0.006459256 |
| ENSG00000 | LINC01426 | 21 | 34745756 | 34784886 | + | 5397 | 0.19 | 0.13 | 0.15 | 0.39 | 0.29 | 0.48 | 2.29989335 | 1.201566965 | 0.00157142 | 0.006541277 |
| ENSG00000 | PKP1 | 1 | 2.01E+08 | 2.01E+08 | + | 6211 | 0.09 | 0.05 | 0.02 | 0 | 0 | 0 | 0.03084248 | -5.018937518 | 0.001573375 | 0.006548044 |
| ENSG00000 | - | 21 | 44978832 | 44979274 | + | 443 | 6.51 | 3.74 | 6.75 | 3.01 | 2.76 | 2.63 | 0.47455093 | -1.075365163 | 0.001610904 | 0.00668604 |
| ENSG00000 | DGCR6 | 22 | 18906028 | 18914238 | + | 5165 | 0.08 | 0.41 | 0.33 | 0.04 | 0.13 | 0.06 | 0.26551501 | -1.9131347 | 0.001635038 | 0.006777732 |
| ENSG00000 | ESM1 | 5 | 54977867 | 55022671 | - | 2692 | 0.2 | 0.21 | 0.1 | 0.57 | 0.5 | 0.48 | 2.88862382 | 1.530382337 | 0.00166291 | 0.006888951 |
| ENSG00000 | C6orf52 | 6 | 10671418 | 10694797 | - | 891 | 2.23 | 2.33 | 3.7 | 1.78 | 1.12 | 1.11 | 0.46564906 | -1.102685014 | 0.001671517 | 0.006920284 |
| ENSG00000 | CLDN14 | 21 | 36460621 | 36576569 | - | 2382 | 0.04 | 0.22 | 0.04 | 0.43 | 0.19 | 0.81 | 4.54205891 | 2.183346418 | 0.001711669 | 0.007063066 |
| ENSG00000 | SOX9-AS1 | 17 | 72067099 | 72237203 | - | 15844 | 0.01 | 0 | 0 | 0.04 | 0.03 | 0.04 | 10.8356837 | 3.43771828 | 0.001787621 | 0.007343903 |
| ENSG00000 | SPRR2D | 1 | 1.53E+08 | 1.53E+08 | - | 1052 | 0.14 | 0.2 | 0.49 | 1.69 | 0.9 | 0.66 | 3.77144466 | 1.915117256 | 0.001800188 | 0.007391437 |
| ENSG00000 | LINC00161 | 21 | 28539318 | 28540355 | + | 870 | 1.54 | 1.61 | 1.66 | 0.44 | 0.64 | 0.74 | 0.36473243 | -1.455089599 | 0.001813422 | 0.007441166 |
| ENSG00000 | NCKAP1L | 12 | 54497752 | 54548243 | + | 10560 | 0 | 0 | 0 | 0.03 | 0.06 | 0.04 | 23.5113806 | 4.555287353 | 0.001833055 | 0.007515526 |
| ENSG00000 | CPEB1-AS1 | 15 | 82647770 | 82692820 | + | 5014 | 0.05 | 0.09 | 0.06 | 0.29 | 0.24 | 0.16 | 3.28524855 | 1.716002524 | 0.001867739 | 0.007641973 |
| ENSG00000 | FGL2 | 7 | 77193369 | 77199848 | - | 6480 | 0.03 | 0.02 | 0.05 | 0.08 | 0.13 | 0.22 | 4.42161287 | 2.144572718 | 0.001873765 | 0.007663473 |
| ENSG00000 | SCIRT | 6 | 43931572 | 44074655 | - | 3691 | 0.44 | 0.31 | 0.4 | 0.12 | 0.17 | 0.16 | 0.36992366 | -1.434700508 | 0.001933628 | 0.007893302 |
| ENSG00000 | GRID2 | 4 | 92303966 | 93810157 | + | 10617 | 0.06 | 0.1 | 0.09 | 0.03 | 0.01 | 0.03 | 0.23257858 | -2.104209861 | 0.001951358 | 0.007961173 |
| ENSG00000 | NGF | 1 | 1.15E+08 | 1.15E+08 | - | 4110 | 0.39 | 0.19 | 0.29 | 0.54 | 0.74 | 0.58 | 2.07440346 | 1.052696518 | 0.001979104 | 0.008062949 |
| ENSG00000 | PDPR2P | 16 | 74332402 | 74368240 | - | 3828 | 0.39 | 0.41 | 0.42 | 0.17 | 0.12 | 0.21 | 0.39193568 | -1.351311175 | 0.001979143 | 0.008062949 |
| ENSG00000 | GGN | 19 | 38384267 | 38388082 | - | 2445 | 0.63 | 0.72 | 0.78 | 0.39 | 0.2 | 0.33 | 0.41643595 | -1.263833493 | 0.002008856 | 0.008166568 |
| ENSG00000 | FAM83C-AS1 | 20 | 35285251 | 35285756 | + | 352 | 0.42 | 0.15 | 0.88 | 3.25 | 3 | 1.16 | 4.90640679 | 2.294666854 | 0.002011388 | 0.00817255 |
| ENSG00000 | - | 3 | 16647666 | 16697604 | - | 1483 | 0.27 | 0.1 | 0.14 | 0.99 | 0.45 | 0.63 | 3.83893843 | 1.940707422 | 0.002021888 | 0.008203478 |

| | | | | | | | | | | | | | | | | |
|---|---|---|---|---|---|---|---|---|---|---|---|---|---|---|---|---|
| ENSG00000 | ANO7 | 2 | 2.41E+08 | 2.41E+08 | + | 6294 | 0.15 | 0.2 | 0.23 | 0.08 | 0.05 | 0.06 | 0.32966936 | -1.600908284 | 0.002049341 | 0.008308234 |
| ENSG00000 | - | 7 | 1.51E+08 | 1.51E+08 | - | 1230 | 1.29 | 2.15 | 1.13 | 0.41 | 0.54 | 0.95 | 0.40225831 | -1.31380586 | 0.002051891 | 0.008315032 |
| ENSG00000 | - | 7 | 12469621 | 12542222 | + | 3737 | 0.03 | 0.06 | 0.03 | 0.25 | 0.12 | 0.23 | 5.33031779 | 2.414221547 | 0.002068921 | 0.008382333 |
| ENSG00000 | GCGR | 17 | 81804132 | 81814008 | + | 3236 | 0.86 | 0.5 | 0.86 | 0.29 | 0.45 | 0.34 | 0.47161587 | -1.084315841 | 0.002077074 | 0.008408515 |
| ENSG00000 | SPANXB1 | X | 1.41E+08 | 1.41E+08 | + | 466 | 0.32 | 0.44 | 0.44 | 3.54 | 1.07 | 1.25 | 4.64667303 | 2.21619813 | 0.002084205 | 0.008427088 |
| ENSG00000 | C3orf85 | 3 | 1.09E+08 | 1.09E+08 | + | 3434 | 0.52 | 0.47 | 0.83 | 0.44 | 0.21 | 0.12 | 0.4056522 | -1.301684795 | 0.002099639 | 0.008487765 |
| ENSG00000 | C1QL4 | 12 | 49332409 | 49337188 | - | 2073 | 1.06 | 0.67 | 0.97 | 0.37 | 0.38 | 0.48 | 0.43512206 | -1.200507932 | 0.002216125 | 0.008916966 |
| ENSG00000 | INE1 | X | 47204921 | 47205865 | + | 945 | 0.37 | 0.44 | 0.38 | 1.28 | 1.88 | 0.74 | 3.15895504 | 1.659447402 | 0.002275706 | 0.00913452 |
| ENSG00000 | PCDHB11 | 5 | 1.41E+08 | 1.41E+08 | + | 4170 | 0.43 | 0.5 | 0.58 | 0.24 | 0.21 | 0.28 | 0.47018421 | -1.088702017 | 0.002356549 | 0.009426018 |
| ENSG00000 | SLC38A4-AS1 | 12 | 46383433 | 46876784 | + | 5227 | 0.54 | 0.47 | 0.39 | 0.17 | 0.24 | 0.3 | 0.49263802 | -1.021400122 | 0.002359693 | 0.009435449 |
| ENSG00000 | PDK1-AS1 | 2 | 1.72E+08 | 1.73E+08 | - | 2447 | 0.85 | 1.08 | 0.95 | 0.52 | 0.48 | 0.5 | 0.49937559 | -1.001802804 | 0.00238955 | 0.009547157 |
| ENSG00000 | RAB11FIP1 | 8 | 37858618 | 37899497 | - | 9632 | 0.05 | 0.11 | 0.02 | 0.01 | 0.01 | 0 | 0.06714395 | -3.896598672 | 0.00241951 | 0.009655218 |
| ENSG00000 | - | 20 | 40696499 | 40698616 | - | 1669 | 0.98 | 0.9 | 1.42 | 0.68 | 0.47 | 0.31 | 0.42417232 | -1.237277603 | 0.002472995 | 0.009850861 |
| ENSG00000 | MIR4280HG | 5 | 87048858 | 87138397 | - | 6281 | 0.3 | 0.24 | 0.31 | 0.07 | 0.12 | 0.18 | 0.42175651 | -1.24551776 | 0.002477537 | 0.009866978 |
| ENSG00000 | SIRLNT | 8 | 40298697 | 40353133 | - | 1974 | 0 | 0 | 0.05 | 0.19 | 0.23 | 0.38 | 14.9575908 | 3.902805917 | 0.00248077 | 0.0098741 |
| ENSG00000 | - | 21 | 32841496 | 32841811 | - | 316 | 0.16 | 0.66 | 2.45 | 0 | 0 | 0 | 0.03059519 | -5.030551218 | 0.002480815 | 0.0098741 |
| ENSG00000 | KCNK6 | 19 | 38319845 | 38332076 | + | 6168 | 0.02 | 0.04 | 0.02 | 0.13 | 0.09 | 0.15 | 4.83314068 | 2.272960989 | 0.002525417 | 0.010031544 |
| ENSG00000 | - | 10 | 33684755 | 33709868 | + | 1986 | 0.05 | 0.05 | 0.08 | 0.51 | 0.14 | 0.44 | 5.81617721 | 2.540071228 | 0.002533331 | 0.010058962 |
| ENSG00000 | CHCHD3P3 | 1 | 27200834 | 27201473 | - | 640 | 0.7 | 0.4 | 0.32 | 0 | 0 | 0 | 0.03424648 | -4.867900579 | 0.002620093 | 0.010372397 |
| ENSG00000 | - | 17 | 77444704 | 77447167 | + | 2464 | 0.08 | 0.13 | 0.33 | 0 | 0 | 0.02 | 0.04591666 | -4.444838413 | 0.002648263 | 0.010470313 |
| ENSG00000 | HLCS-AS1 | 21 | 36966461 | 36975164 | + | 4412 | 0.08 | 0.12 | 0.11 | 0.2 | 0.3 | 0.38 | 2.84528453 | 1.508572929 | 0.002706258 | 0.010687973 |
| ENSG00000 | SERF1B | 5 | 70025247 | 70043113 | + | 3051 | 0.44 | 0.95 | 0.42 | 0.15 | 0.29 | 0.31 | 0.39710684 | -1.332400879 | 0.002738136 | 0.01080813 |
| ENSG00000 | SNORD3A | 17 | 19188016 | 19188714 | + | 699 | 0.5 | 1.11 | 0.22 | 1.45 | 3.26 | 1.33 | 3.18782122 | 1.672570721 | 0.002742523 | 0.010822598 |
| ENSG00000 | IL6ST-DT | 5 | 55995153 | 56003649 | + | 2563 | 0.04 | 0.18 | 0.14 | 0.35 | 0.46 | 0.45 | 3.3706001 | 1.753005471 | 0.002845031 | 0.011189368 |
| ENSG00000 | CDHR2 | 5 | 1.77E+08 | 1.77E+08 | + | 5397 | 0.32 | 0.5 | 0.34 | 0.07 | 0.25 | 0.21 | 0.43658705 | -1.195658772 | 0.002859212 | 0.011227377 |
| ENSG00000 | SMG1P2 | 16 | 29527568 | 29594966 | - | 4221 | 0.26 | 0.16 | 0.39 | 1.34 | 1.49 | 0.4 | 3.82987176 | 1.937296085 | 0.002878558 | 0.011292192 |
| ENSG00000 | ELOA-AS1 | 1 | 23706901 | 23778325 | - | 3246 | 0.35 | 0.32 | 0.35 | 0.61 | 0.98 | 0.59 | 2.05412789 | 1.038526007 | 0.002884226 | 0.011312195 |
| ENSG00000 | - | 6 | 1.01E+08 | 1.01E+08 | + | 1544 | 0.35 | 0.27 | 0.27 | 1.23 | 0.86 | 0.53 | 2.82216147 | 1.496800533 | 0.002960194 | 0.011578587 |
| ENSG00000 | - | 18 | 24725762 | 24986980 | + | 8281 | 0.04 | 0.06 | 0.01 | 0 | 0 | 0 | 0.03409048 | -4.87448724 | 0.002977203 | 0.011640126 |
| ENSG00000 | LCN9 | 9 | 1.36E+08 | 1.36E+08 | + | 1657 | 0.33 | 0.31 | 0.5 | 1.23 | 0.84 | 0.84 | 2.44851009 | 1.291904142 | 0.002982429 | 0.011653684 |
| ENSG00000 | RNF43 | 17 | 58353676 | 58417595 | - | 6329 | 0.1 | 0.11 | 0.18 | 0.37 | 0.33 | 0.22 | 2.24930585 | 1.169479845 | 0.003029061 | 0.011809307 |
| ENSG00000 | - | 14 | 87710419 | 87872291 | - | 1845 | 0.32 | 0.06 | 0.11 | 0.59 | 0.45 | 0.76 | 3.48729115 | 1.802106816 | 0.003029976 | 0.011809307 |
| ENSG00000 | LINC00598 | 13 | 40079106 | 40535807 | - | 19673 | 0.03 | 0.01 | 0.03 | 0.09 | 0.06 | 0.04 | 3.07774449 | 1.621873467 | 0.003078796 | 0.01196707 |
| ENSG00000 | - | 5 | 73213930 | 73295258 | + | 4119 | 0.02 | 0.06 | 0.03 | 0.19 | 0.15 | 0.21 | 4.69769782 | 2.231953916 | 0.003080934 | 0.011972711 |
| ENSG00000 | - | 5 | 8207019 | 8366437 | - | 3087 | 0.45 | 0.25 | 0.5 | 0.06 | 0.2 | 0.15 | 0.33220303 | -1.589862866 | 0.003093218 | 0.012015752 |
| ENSG00000 | ZSCAN12P1 | 6 | 28091137 | 28093664 | + | 1674 | 0.86 | 0.71 | 0.46 | 0.87 | 1.66 | 1.95 | 2.12340378 | 1.086378735 | 0.003094932 | 0.012020064 |
| ENSG00000 | CEP192-DT | 18 | 12984694 | 12991173 | - | 474 | 1.26 | 0.98 | 0.98 | 3.48 | 1.76 | 3.81 | 2.69388841 | 1.429690091 | 0.003138488 | 0.012160725 |
| ENSG00000 | ESRRG | 1 | 2.17E+08 | 2.17E+08 | - | 10932 | 0.16 | 0.11 | 0.06 | 0.26 | 0.21 | 0.23 | 2.05848299 | 1.041581531 | 0.003142529 | 0.012174009 |
| ENSG00000 | EVA1A-AS | 2 | 75524068 | 75542706 | + | 1185 | 1.26 | 1.22 | 0.91 | 0.38 | 0.38 | 0.59 | 0.37997194 | -1.396035222 | 0.00339071 | 0.013031363 |
| ENSG00000 | BTN2A3P | 6 | 26421391 | 26432383 | + | 4217 | 0.44 | 0.48 | 0.64 | 0.23 | 0.22 | 0.33 | 0.48599933 | -1.040973766 | 0.003401538 | 0.013062877 |
| ENSG00000 | - | 19 | 19516227 | 19536076 | + | 1846 | 0.65 | 0.31 | 0.78 | 0.21 | 0.12 | 0.22 | 0.30200823 | -1.727340215 | 0.003411671 | 0.013094204 |
| ENSG00000 | CHRM5 | 15 | 33968497 | 34067458 | + | 6041 | 0.03 | 0.07 | 0.04 | 0 | 0 | 0 | 0.0360085 | -4.795518683 | 0.003427621 | 0.01314274 |
| ENSG00000 | PCOLCE-AS1 | 7 | 1.01E+08 | 1.01E+08 | - | 3265 | 0.34 | 0.14 | 0.33 | 0.08 | 0.09 | 0.05 | 0.2570424 | -1.959921741 | 0.003453881 | 0.013233222 |
| ENSG00000 | LINC01711 | 20 | 58634772 | 58635738 | + | 967 | 1.54 | 1.93 | 2.34 | 1.25 | 0.46 | 0.96 | 0.43852054 | -1.18928366 | 0.003482603 | 0.013327862 |
| ENSG00000 | EPS15P1 | 7 | 46781373 | 46783443 | + | 2071 | 0.05 | 0.18 | 0.07 | 0.4 | 0.4 | 0.42 | 3.97023869 | 1.989225744 | 0.003541366 | 0.01353451 |
| ENSG00000 | - | 1 | 36329630 | 36335406 | + | 4878 | 0.34 | 0.21 | 0.36 | 0.14 | 0.09 | 0.14 | 0.39839526 | -1.327727625 | 0.003544055 | 0.013542186 |
| ENSG00000 | XKR3 | 22 | 16783412 | 16825411 | - | 1811 | 0.19 | 0.31 | 0 | 0.67 | 0.49 | 0.71 | 3.53162286 | 1.820331285 | 0.003559481 | 0.013593295 |
| ENSG00000 | ZNF618 | 9 | 1.14E+08 | 1.14E+08 | + | 10386 | 0.23 | 0.17 | 0.19 | 0.14 | 0.09 | 0.06 | 0.4668539 | -1.098956957 | 0.003622917 | 0.013809029 |
| ENSG00000 | LINC02280 | 14 | 1.05E+08 | 1.05E+08 | - | 3816 | 0.48 | 0.48 | 0.36 | 0.13 | 0.25 | 0.21 | 0.43530933 | -1.199887162 | 0.003678494 | 0.013994042 |
| ENSG00000 | MEGF6 | 1 | 3487951 | 3611508 | - | 9021 | 0.06 | 0.2 | 0.1 | 0.04 | 0.05 | 0.01 | 0.28026766 | -1.835122832 | 0.003713267 | 0.014112827 |
| ENSG00000 | SPTBN2 | 11 | 66682497 | 66744670 | - | 12609 | 0.12 | 0.11 | 0.11 | 0.03 | 0.07 | 0.04 | 0.39362717 | -1.345098276 | 0.003720651 | 0.014138188 |

| | | | | | | | | | | | | | | | | |
|---|---|---|---|---|---|---|---|---|---|---|---|---|---|---|---|---|
| ENSG00000 | LINC02846 | 9 | 1.36E+08 | 1.36E+08 | - | 1334 | 1.04 | 1.01 | 0.81 | 0.05 | 0.67 | 0.22 | 0.31905986 | -1.64810097 | 0.003773927 | 0.014321476 |
| ENSG00000 | - | 2 | 1.77E+08 | 1.77E+08 | + | 1624 | 0.37 | 0.61 | 0.22 | 0.08 | 0.07 | 0.07 | 0.17529234 | -2.512165119 | 0.003802492 | 0.014418871 |
| ENSG00000 | FABP4 | 8 | 81478419 | 81483236 | - | 1369 | 0.25 | 0.19 | 0.26 | 0.74 | 0.61 | 0.94 | 3.11051037 | 1.637151314 | 0.003864117 | 0.014637996 |
| ENSG00000 | TNNI3 | 19 | 55151767 | 55157773 | - | 2034 | 0.27 | 0.13 | 0.41 | 0.87 | 0.44 | 0.92 | 2.6688514 | 1.416218982 | 0.003915576 | 0.014794076 |
| ENSG00000 | KLRA1P | 12 | 10588063 | 10599669 | - | 2457 | 0.57 | 0.93 | 0.75 | 0.21 | 0.52 | 0.28 | 0.43698384 | -1.194348151 | 0.003925847 | 0.014830066 |
| ENSG00000 | - | 4 | 1.13E+08 | 1.13E+08 | - | 498 | 36.17 | 89.33 | 60.84 | 26.91 | 24.88 | 36.04 | 0.45417387 | -1.138683398 | 0.003948486 | 0.014898603 |
| ENSG00000 | SCN5A | 3 | 38548057 | 38649687 | - | 10028 | 0.07 | 0.02 | 0.04 | 0 | 0 | 0.01 | 0.0501868 | -4.31654822 | 0.004067058 | 0.015282209 |
| ENSG00000 | THY1 | 11 | 1.19E+08 | 1.19E+08 | - | 6900 | 0.14 | 0.08 | 0.15 | 0.04 | 0.02 | 0.04 | 0.26808608 | -1.899231759 | 0.004195563 | 0.015708674 |
| ENSG00000 | MIR138-1 | 3 | 44114212 | 44114310 | + | 99 | 7.04 | 10.99 | 8.85 | 1.28 | 2.24 | 4.12 | 0.2763476 | -1.855443987 | 0.004209045 | 0.015742662 |
| ENSG00000 | PKP3 | 11 | 392614 | 404908 | + | 3795 | 0.31 | 0.11 | 0.14 | 0.45 | 0.42 | 0.51 | 2.36626128 | 1.242609381 | 0.004217117 | 0.015765625 |
| ENSG00000 | RAB43 | 3 | 1.29E+08 | 1.29E+08 | - | 4991 | 0.01 | 0.04 | 0.1 | 0.2 | 0.2 | 0.18 | 3.62360505 | 1.857425718 | 0.004268252 | 0.01594181 |
| ENSG00000 | INMT | 7 | 30697985 | 30757602 | + | 3376 | 0.03 | 0.17 | 0.06 | 0 | 0 | 0 | 0.035855 | -4.801681709 | 0.004302997 | 0.016062531 |
| ENSG00000 | ZNF256 | 19 | 57940833 | 57947706 | - | 2207 | 0.27 | 0.23 | 0.19 | 0 | 0.05 | 0.03 | 0.11005882 | -3.183653298 | 0.004333729 | 0.016168143 |
| ENSG00000 | - | 17 | 40921430 | 40975926 | + | 815 | 0 | 0 | 0 | 0.08 | 0.41 | 0.57 | 29.6664565 | 4.890760712 | 0.004345796 | 0.01620404 |
| ENSG00000 | PDCL3P4 | 3 | 1.02E+08 | 1.02E+08 | + | 5804 | 0.41 | 0.4 | 0.29 | 0.26 | 0.15 | 0.15 | 0.48866639 | -1.033078227 | 0.004361872 | 0.016254838 |
| ENSG00000 | RGS16 | 1 | 1.83E+08 | 1.83E+08 | - | 2408 | 0.04 | 0.02 | 0.15 | 0.45 | 0.18 | 0.36 | 4.50750757 | 2.172329915 | 0.004388558 | 0.016342035 |
| ENSG00000 | PDCD4-AS1 | 10 | 1.11E+08 | 1.11E+08 | - | 925 | 0.86 | 0.45 | 0.67 | 1.92 | 1.56 | 1.45 | 2.39002554 | 1.257026034 | 0.004529374 | 0.016822301 |
| ENSG00000 | DNAH1 | 3 | 52316319 | 52400492 | + | 13873 | 0.18 | 0.12 | 0.16 | 0.06 | 0.08 | 0.09 | 0.49826116 | -1.005025983 | 0.004584463 | 0.016991994 |
| ENSG00000 | COL11A2 | 6 | 33162681 | 33192499 | - | 7364 | 0.14 | 0.12 | 0.17 | 0.09 | 0.05 | 0 | 0.29009603 | -1.78539752 | 0.004619176 | 0.017095528 |
| ENSG00000 | MYB | 6 | 1.35E+08 | 1.35E+08 | + | 5497 | 0.05 | 0.04 | 0.06 | 0.21 | 0.1 | 0.21 | 3.37009343 | 1.752788589 | 0.00465562 | 0.017217224 |
| ENSG00000 | IBA57-DT | 1 | 2.28E+08 | 2.28E+08 | - | 641 | 1.24 | 1.37 | 0.32 | 0.2 | 0 | 0.27 | 0.1517628 | -2.720109851 | 0.004672673 | 0.017277075 |
| ENSG00000 | LINC01940 | 2 | 2.39E+08 | 2.39E+08 | - | 3489 | 0.61 | 0.3 | 0.28 | 0.11 | 0.11 | 0.23 | 0.36727173 | -1.44508024 | 0.004675873 | 0.01728569 |
| ENSG00000 | MAGEB6 | X | 26192440 | 26195646 | + | 1949 | 0.05 | 0.05 | 0 | 0.52 | 0.23 | 0.12 | 7.93231052 | 2.987741155 | 0.004777928 | 0.017591018 |
| ENSG00000 | ATP13A4-AS1 | 3 | 1.94E+08 | 1.94E+08 | + | 1046 | 0 | 0 | 0 | 0.18 | 0.37 | 0.22 | 27.8241912 | 4.798267846 | 0.004785903 | 0.017613857 |
| ENSG00000 | - | 19 | 8008729 | 8016025 | + | 421 | 3.78 | 1.6 | 2.2 | 0.45 | 1.19 | 0.83 | 0.31595749 | -1.662197627 | 0.004797986 | 0.017651795 |
| ENSG00000 | SELENOO-AS1 | 22 | 50208461 | 50209542 | - | 640 | 2.18 | 1.94 | 1.21 | 1.19 | 0.26 | 0.36 | 0.32103354 | -1.639204047 | 0.004799458 | 0.017653945 |
| ENSG00000 | KCNA1 | 12 | 4909905 | 4918256 | + | 7985 | 0.24 | 0.22 | 0.17 | 0.12 | 0.06 | 0.11 | 0.44540113 | -1.166822887 | 0.005020989 | 0.018378724 |
| ENSG00000 | STK24-AS1 | 13 | 98577244 | 98578830 | + | 1278 | 0.78 | 0.85 | 1.13 | 0.25 | 0.39 | 0.41 | 0.36879485 | -1.439109591 | 0.005032988 | 0.018410773 |
| ENSG00000 | ROCR | 17 | 72021851 | 72058073 | - | 3586 | 0 | 0 | 0.01 | 0.09 | 0.15 | 0.07 | 19.4303422 | 4.280239406 | 0.005079365 | 0.01856307 |
| ENSG00000 | NAIP | 5 | 70968166 | 71025339 | - | 10055 | 0.16 | 0.18 | 0.09 | 0.03 | 0.08 | 0.06 | 0.38426061 | -1.379842991 | 0.005085696 | 0.018579654 |
| ENSG00000 | - | 1 | 1.55E+08 | 1.55E+08 | + | 1224 | 0.37 | 0.51 | 0.08 | 0 | 0 | 0.05 | 0.05195481 | -4.266598793 | 0.005094569 | 0.018608652 |
| ENSG00000 | DRAXIN | 1 | 11691710 | 11725857 | + | 7365 | 0.07 | 0.1 | 0.08 | 0.01 | 0.03 | 0.01 | 0.18306385 | -2.44958119 | 0.00511066 | 0.0186622 |
| ENSG00000 | - | 18 | 35716370 | 35717978 | + | 1609 | 1.36 | 1.42 | 1.22 | 0.28 | 0.93 | 0.76 | 0.47935607 | -1.060830387 | 0.005132898 | 0.018731451 |
| ENSG00000 | - | 12 | 9398355 | 9414851 | - | 400 | 2.11 | 1.94 | 1.29 | 0.32 | 0.83 | 0 | 0.20947312 | -2.255162961 | 0.005268549 | 0.019180722 |
| ENSG00000 | - | 4 | 1.71E+08 | 1.71E+08 | + | 1133 | 1.05 | 0.73 | 1.64 | 0.67 | 0.34 | 0.31 | 0.36902468 | -1.438210786 | 0.005287592 | 0.01923596 |
| ENSG00000 | DDX11-AS1 | 12 | 31019434 | 31073847 | - | 4240 | 0.43 | 0.37 | 0.4 | 0.19 | 0.18 | 0.21 | 0.46742953 | -1.097179205 | 0.005308438 | 0.019297675 |
| ENSG00000 | ANPEP | 15 | 89784895 | 89815401 | - | 5046 | 0.17 | 0.21 | 0.15 | 0.05 | 0.08 | 0.03 | 0.29770967 | -1.748021998 | 0.005325343 | 0.019348521 |
| ENSG00000 | ARL14 | 3 | 1.61E+08 | 1.61E+08 | + | 1289 | 0.08 | 0.08 | 0.08 | 0.34 | 0.43 | 0.59 | 5.53843056 | 2.469477214 | 0.005420566 | 0.019647821 |
| ENSG00000 | WTAPP1 | 11 | 1.03E+08 | 1.03E+08 | + | 3331 | 0.04 | 0 | 0.06 | 0.13 | 0.13 | 0.33 | 5.4093752 | 2.435461969 | 0.005520244 | 0.01996182 |
| ENSG00000 | NPIPA7 | 16 | 16379140 | 16393972 | + | 1334 | 0.48 | 1.4 | 1.31 | 0.48 | 0.17 | 0.57 | 0.36335765 | -1.460537827 | 0.005529415 | 0.019990116 |
| ENSG00000 | - | 2 | 99405218 | 99405843 | + | 626 | 1.43 | 2.73 | 1.48 | 0.41 | 1.06 | 0.56 | 0.34980291 | -1.515385817 | 0.005530079 | 0.019990116 |
| ENSG00000 | SERTAD4-AS1 | 1 | 2.1E+08 | 2.1E+08 | - | 1093 | 2.19 | 1.28 | 1.08 | 0.64 | 1.02 | 0.27 | 0.40674702 | -1.297796334 | 0.005541004 | 0.020025965 |
| ENSG00000 | - | 17 | 8365563 | 8381328 | + | 1408 | 0.95 | 0.18 | 0.22 | 0.09 | 0.12 | 0 | 0.14708936 | -2.765235164 | 0.00556038 | 0.020081394 |
| ENSG00000 | COL28A1 | 7 | 7356203 | 7535873 | - | 6639 | 0.16 | 0.09 | 0.12 | 0.04 | 0.03 | 0.04 | 0.27885627 | -1.842406377 | 0.005612309 | 0.020257898 |
| ENSG00000 | AATK | 17 | 81110487 | 81166221 | - | 13177 | 0.09 | 0.05 | 0.1 | 0.02 | 0.05 | 0.01 | 0.30692688 | -1.704033107 | 0.005648553 | 0.020362848 |
| ENSG00000 | ZGLP1 | 19 | 10304803 | 10309880 | - | 2431 | 0.49 | 0.87 | 0.59 | 0.24 | 0.34 | 0.31 | 0.43941811 | -1.186333777 | 0.005875337 | 0.021088643 |
| ENSG00000 | CDC42-AS1 | 1 | 22028317 | 22052927 | - | 2574 | 0.81 | 0.58 | 0.54 | 0.49 | 0.24 | 0.14 | 0.42505679 | -1.234272502 | 0.00591147 | 0.021203028 |
| ENSG00000 | LINC01152 | 17 | 72030291 | 72041310 | + | 4164 | 0.16 | 0.07 | 0.09 | 0.18 | 0.44 | 0.28 | 2.74770439 | 1.458226804 | 0.005924242 | 0.021245005 |
| ENSG00000 | NLGN4Y | Y | 14522573 | 14845654 | + | 8593 | 0.19 | 0.19 | 0.2 | 0.1 | 0.11 | 0.08 | 0.47084085 | -1.086688611 | 0.005936003 | 0.021283342 |
| ENSG00000 | SPNS2 | 17 | 4498881 | 4539035 | + | 4068 | 0 | 0 | 0.03 | 0.05 | 0.18 | 0.11 | 13.0571364 | 3.706766624 | 0.005958929 | 0.021353993 |
| ENSG00000 | - | 7 | 227554 | 229557 | - | 2004 | 0.4 | 0.13 | 0.28 | 0.06 | 0.06 | 0 | 0.13981085 | -2.838451764 | 0.005979701 | 0.021405286 |

| | | | | | | | | | | | | | | | | |
|---|---|---|---|---|---|---|---|---|---|---|---|---|---|---|---|---|
| ENSG00000 | - | 20 | 62928621 | 62929297 | - | 677 | 1.1 | 0.61 | 0.91 | 0.19 | 0.16 | 0.17 | 0.19121009 | -2.38676946 | 0.006086495 | 0.021728896 |
| ENSG00000 | PADI1 | 1 | 17205128 | 17246007 | + | 4903 | 0.26 | 0.44 | 0.26 | 0.16 | 0.07 | 0.2 | 0.4203364 | -1.250383713 | 0.006135639 | 0.021872925 |
| ENSG00000 | ALK | 2 | 29192774 | 29921586 | - | 7382 | 0.23 | 0.25 | 0.18 | 0.13 | 0.1 | 0.09 | 0.45774216 | -1.127392912 | 0.006197528 | 0.022061912 |
| ENSG00000 | FMO1 | 1 | 1.71E+08 | 1.71E+08 | + | 3602 | 0.1 | 0.36 | 0.2 | 0.04 | 0.09 | 0.03 | 0.23788359 | -2.071672327 | 0.006209233 | 0.02209567 |
| ENSG00000 | PRR4 | 12 | 10845849 | 10849475 | - | 1310 | 0.91 | 0.51 | 0.98 | 0.15 | 0.34 | 0.36 | 0.33888171 | -1.561146323 | 0.006221732 | 0.022132227 |
| ENSG00000 | - | 16 | 29913144 | 29915337 | - | 2194 | 0.52 | 0.68 | 0.26 | 0.2 | 0.13 | 0.19 | 0.33645487 | -1.571515099 | 0.006234818 | 0.022170844 |
| ENSG00000 | - | 7 | 19112474 | 19114271 | + | 1711 | 0.84 | 1.57 | 1.05 | 0.52 | 0.06 | 0.07 | 0.1785061 | -2.485954753 | 0.006282813 | 0.022328517 |
| ENSG00000 | DIO1 | 1 | 53891239 | 53911086 | + | 2282 | 0.33 | 0.18 | 0.14 | 0.03 | 0 | 0.05 | 0.1161617 | -3.105793563 | 0.006287946 | 0.022339792 |
| ENSG00000 | EPCAM-DT | 2 | 47192405 | 47345074 | - | 3594 | 0.01 | 0.01 | 0 | 0.11 | 0.12 | 0.13 | 12.1979644 | 3.608568505 | 0.006290533 | 0.022344988 |
| ENSG00000 | - | 1 | 1.2E+08 | 1.2E+08 | + | 853 | 0.35 | 0.43 | 0.66 | 1.34 | 1.37 | 1.3 | 2.68354754 | 1.424141444 | 0.006326221 | 0.022463735 |
| ENSG00000 | EPHB1 | 3 | 1.35E+08 | 1.35E+08 | + | 5843 | 0.01 | 0.01 | 0 | 0.05 | 0.07 | 0.1 | 12.190497 | 3.607685038 | 0.006588614 | 0.023295625 |
| ENSG00000 | ATP13A5 | 3 | 1.93E+08 | 1.93E+08 | - | 6206 | 0.07 | 0.05 | 0.01 | 0 | 0 | 0 | 0.03853164 | -4.697812764 | 0.00667949 | 0.023566652 |
| ENSG00000 | PSG9 | 19 | 43211791 | 43269530 | - | 5048 | 0.12 | 0.12 | 0.09 | 0.04 | 0 | 0.02 | 0.17086889 | -2.54903836 | 0.006722977 | 0.02369486 |
| ENSG00000 | LINC01389 | 1 | 47380928 | 47408477 | - | 483 | 1.65 | 0.43 | 1.28 | 0.13 | 0.35 | 0 | 0.13829972 | -2.854129834 | 0.006752365 | 0.023777366 |
| ENSG00000 | TMEM86B | 19 | 55226638 | 55228784 | - | 2147 | 0.63 | 1.09 | 1.34 | 0.56 | 0.39 | 0.62 | 0.49581727 | -1.012119573 | 0.006782214 | 0.023865568 |
| ENSG00000 | - | 2 | 2.17E+08 | 2.17E+08 | - | 2879 | 0.14 | 0.14 | 0.18 | 0 | 0 | 0.04 | 0.08545258 | -3.548732113 | 0.006849629 | 0.024077226 |
| ENSG00000 | LINC01176 | 7 | 30390885 | 30412375 | + | 5096 | 0.24 | 0.38 | 0.12 | 0.05 | 0.09 | 0.14 | 0.35804282 | -1.481795975 | 0.006865534 | 0.024116082 |
| ENSG00000 | COL4A1 | 13 | 1.1E+08 | 1.1E+08 | - | 14662 | 0.18 | 0.12 | 0.17 | 0.03 | 0.14 | 0.06 | 0.47416979 | -1.076524339 | 0.006944292 | 0.024375508 |
| ENSG00000 | MANCR | 10 | 4581295 | 4678216 | - | 3143 | 0.44 | 0.41 | 0.57 | 0.14 | 0.3 | 0.2 | 0.43863552 | -1.188905466 | 0.007012007 | 0.024561168 |
| ENSG00000 | PCAT19 | 19 | 41449520 | 41501255 | - | 8213 | 0.08 | 0.06 | 0.15 | 0.22 | 0.26 | 0.17 | 2.19468076 | 1.134011102 | 0.007049188 | 0.024674018 |
| ENSG00000 | - | 5 | 58114598 | 58145996 | + | 789 | 0.38 | 1.44 | 0.65 | 0.16 | 0.21 | 0.15 | 0.2030708 | -2.29994532 | 0.007071503 | 0.024743417 |
| ENSG00000 | - | 17 | 7858943 | 7866083 | + | 7141 | 0.13 | 0.17 | 0.18 | 0.07 | 0.09 | 0.01 | 0.34664952 | -1.528450325 | 0.007122492 | 0.024913063 |
| ENSG00000 | - | 11 | 66269832 | 66278525 | - | 2792 | 0.29 | 0.19 | 0.17 | 0.02 | 0.02 | 0.08 | 0.19105753 | -2.387920972 | 0.007127085 | 0.024924741 |
| ENSG00000 | GOLGA2P5 | 12 | 1E+08 | 1E+08 | - | 10957 | 0.02 | 0.07 | 0.01 | 0.14 | 0.07 | 0.11 | 3.00032411 | 1.585118355 | 0.007146639 | 0.024988731 |
| ENSG00000 | IRAG2 | 12 | 25004342 | 25108335 | + | 6611 | 0.14 | 0.11 | 0.1 | 0.01 | 0.05 | 0.04 | 0.26889824 | -1.8948678 | 0.007228598 | 0.025248668 |
| ENSG00000 | ZNF474 | 5 | 1.22E+08 | 1.22E+08 | + | 2731 | 0.33 | 0.59 | 0.47 | 0.19 | 0.18 | 0.21 | 0.40464205 | -1.305281858 | 0.007328296 | 0.025574135 |
| ENSG00000 | CACNA2D1-AS1 | 7 | 82009177 | 82029955 | + | 2651 | 0.56 | 0.59 | 0.52 | 0.26 | 0.19 | 0.33 | 0.44843091 | -1.157042382 | 0.007395484 | 0.02579081 |
| ENSG00000 | C9orf24 | 9 | 34379019 | 34397828 | - | 1435 | 0.69 | 0.47 | 0.36 | 0 | 0.27 | 0.08 | 0.22830513 | -2.130964826 | 0.007419425 | 0.025853169 |
| ENSG00000 | - | 20 | 58817132 | 58817725 | - | 594 | 0.67 | 1.31 | 1.56 | 0.64 | 0.09 | 0.1 | 0.22047959 | -2.181282984 | 0.007447567 | 0.025940597 |
| ENSG00000 | RBBP4P2 | 3 | 94075912 | 94077175 | - | 1264 | 0.12 | 0.37 | 0.12 | 0 | 0 | 0 | 0.04072022 | -4.618110795 | 0.007488962 | 0.026057395 |
| ENSG00000 | - | 2 | 2.01E+08 | 2.01E+08 | + | 973 | 0.36 | 0.69 | 0.64 | 0.07 | 0.06 | 0.18 | 0.17325572 | -2.529025143 | 0.007543238 | 0.026231615 |
| ENSG00000 | - | 1 | 2.35E+08 | 2.35E+08 | + | 526 | 1.42 | 1.08 | 0.29 | 0.24 | 0.11 | 0 | 0.11624844 | -3.104716724 | 0.007616924 | 0.026442482 |
| ENSG00000 | APP-DT | 21 | 26170871 | 26217381 | + | 4245 | 0.14 | 0.07 | 0.04 | 0.24 | 0.22 | 0.29 | 2.86881075 | 1.520452799 | 0.007755535 | 0.026876753 |
| ENSG00000 | ADGRF2 | 6 | 47656487 | 47695192 | + | 2852 | 0 | 0.04 | 0.05 | 0.24 | 0.14 | 0.18 | 6.04376512 | 2.595447594 | 0.007932771 | 0.027357466 |
| ENSG00000 | SSR4P1 | 21 | 45070952 | 45074165 | - | 2636 | 0.26 | 0.37 | 0.57 | 0.1 | 0.19 | 0.15 | 0.35563965 | -1.491511904 | 0.008059435 | 0.027719267 |
| ENSG00000 | RIBC1 | X | 53422690 | 53431120 | + | 2993 | 0.12 | 0.1 | 0.29 | 0.3 | 0.52 | 0.53 | 2.5347732 | 1.341856666 | 0.008181496 | 0.028083729 |
| ENSG00000 | FLG | 1 | 1.52E+08 | 1.52E+08 | - | 12793 | 0 | 0.02 | 0.05 | 0.08 | 0.06 | 0.09 | 3.47597212 | 1.797416511 | 0.008248635 | 0.028289774 |
| ENSG00000 | ZNF14 | 19 | 19710472 | 19733112 | - | 2963 | 0.05 | 0.07 | 0.05 | 0.43 | 0.15 | 0.16 | 4.0779968 | 2.027860644 | 0.008252507 | 0.028298174 |
| ENSG00000 | - | 16 | 89268104 | 89273044 | + | 3660 | 0.27 | 0.45 | 0.53 | 0.21 | 0.24 | 0.13 | 0.44282773 | -1.175182532 | 0.008309285 | 0.02846832 |
| ENSG00000 | CNDP1 | 18 | 74534500 | 74587212 | + | 7117 | 0.12 | 0.07 | 0.04 | 0.16 | 0.22 | 0.2 | 2.38536909 | 1.254212511 | 0.008322149 | 0.028507484 |
| ENSG00000 | CFP | X | 47623172 | 47630305 | - | 5617 | 0.08 | 0.2 | 0.11 | 0.05 | 0.02 | 0.04 | 0.25918286 | -1.947957801 | 0.008423435 | 0.0288088 |
| ENSG00000 | GRIN3A | 9 | 1.02E+08 | 1.02E+08 | - | 8189 | 0.01 | 0.01 | 0.01 | 0.09 | 0.01 | 0.11 | 6.32893278 | 2.661962245 | 0.0084246 | 0.0288088 |
| ENSG00000 | SMCO3 | 12 | 14804650 | 14814182 | - | 2104 | 0 | 0.05 | 0 | 0.45 | 0.18 | 0.03 | 13.0323872 | 3.704029463 | 0.008549404 | 0.02918038 |
| ENSG00000 | - | 9 | 35491133 | 35491969 | - | 663 | 0.38 | 0.31 | 0.47 | 1.53 | 1.68 | 0.7 | 3.25705301 | 1.7035672 | 0.008593212 | 0.02931481 |
| ENSG00000 | - | 11 | 22829380 | 22945393 | + | 5339 | 0.09 | 0.05 | 0.04 | 0.1 | 0.22 | 0.25 | 3.01622892 | 1.592745927 | 0.008642105 | 0.029461384 |
| ENSG00000 | KCNJ5-AS1 | 11 | 1.29E+08 | 1.29E+08 | - | 3759 | 0.45 | 0.32 | 0.44 | 0.12 | 0.28 | 0.16 | 0.44586081 | -1.165334709 | 0.008689451 | 0.029595988 |
| ENSG00000 | NAIPP2 | 5 | 70094659 | 70128434 | - | 3544 | 0.51 | 0.34 | 0.36 | 0.09 | 0.3 | 0.13 | 0.41852934 | -1.256599327 | 0.008832116 | 0.030026779 |
| ENSG00000 | ABAT | 16 | 8674596 | 8784575 | + | 9713 | 0.03 | 0.08 | 0.06 | 0.13 | 0.14 | 0.15 | 2.3875422 | 1.255526232 | 0.008913506 | 0.030267259 |
| ENSG00000 | KRT8P33 | 5 | 1.23E+08 | 1.23E+08 | - | 1423 | 0.14 | 0.11 | 0.18 | 0.62 | 0.35 | 0.61 | 3.55007034 | 1.827847609 | 0.00895752 | 0.030395952 |
| ENSG00000 | DNAH6 | 2 | 84516455 | 84819589 | + | 13514 | 0.03 | 0.07 | 0.06 | 0.1 | 0.12 | 0.15 | 2.18572206 | 1.128109959 | 0.008962981 | 0.030409294 |
| ENSG00000 | TSSC2 | 11 | 3380918 | 3408981 | + | 3715 | 0.74 | 0.59 | 0.36 | 0.38 | 0.31 | 0.19 | 0.49922331 | -1.002242787 | 0.008978334 | 0.030456186 |

| | | | | | | | | | | | | | | | | |
|---|---|---|---|---|---|---|---|---|---|---|---|---|---|---|---|---|
| ENSG00000 | HCLS1 | 3 | 1.22E+08 | 1.22E+08 | - | 5093 | 0.08 | 0.11 | 0.09 | 0.27 | 0.27 | 0.17 | 2.45955932 | 1.298399851 | 0.009027499 | 0.030607301 |
| ENSG00000 | KREMEN2 | 16 | 2964216 | 2968383 | + | 2342 | 0.36 | 0.44 | 0.64 | 0.14 | 0.26 | 0.17 | 0.38432507 | -1.379601011 | 0.009058723 | 0.030697461 |
| ENSG00000 | - | 1 | 19608114 | 19608568 | + | 455 | 0.55 | 1.71 | 0.57 | 0.14 | 0 | 0.13 | 0.08922355 | -3.486431569 | 0.009083787 | 0.030766668 |
| ENSG00000 | - | 3 | 45795970 | 45863928 | + | 8660 | 0.11 | 0.03 | 0.1 | 0.01 | 0.01 | 0.04 | 0.21794758 | -2.197946894 | 0.009210642 | 0.031144408 |
| ENSG00000 | LINC02958 | 18 | 23957754 | 23982556 | - | 2343 | 0.06 | 0.24 | 0.29 | 0.03 | 0.02 | 0.02 | 0.12301805 | -3.023058091 | 0.009301196 | 0.031428086 |
| ENSG00000 | LYG1 | 2 | 99284238 | 99304742 | - | 1114 | 1.56 | 0.98 | 1.76 | 0.91 | 0.6 | 0.58 | 0.46427334 | -1.106953645 | 0.009323923 | 0.031494175 |
| ENSG00000 | - | 5 | 1.4E+08 | 1.4E+08 | + | 6140 | 0.12 | 0.2 | 0.1 | 0.01 | 0.05 | 0.08 | 0.30189507 | -1.727880912 | 0.009519799 | 0.032043699 |
| ENSG00000 | - | 8 | 1.3E+08 | 1.3E+08 | + | 3369 | 0.01 | 0.02 | 0.05 | 0.08 | 0.13 | 0.26 | 5.95799847 | 2.574827754 | 0.009797667 | 0.032871033 |
| ENSG00000 | - | 5 | 1.41E+08 | 1.41E+08 | + | 2086 | 0 | 0.02 | 0 | 0.18 | 0.11 | 0.17 | 16.6922453 | 4.061106126 | 0.009812229 | 0.032914335 |
| ENSG00000 | C1QTNF12 | 1 | 1242446 | 1246722 | - | 1794 | 0.22 | 0.06 | 0.06 | 0.64 | 0.28 | 0.39 | 3.68011429 | 1.87975057 | 0.010053379 | 0.033660791 |
| ENSG00000 | ZNF420 | 19 | 37007857 | 37130368 | + | 4077 | 0.15 | 0.04 | 0.19 | 0.42 | 0.19 | 0.39 | 2.5553138 | 1.35350047 | 0.010241959 | 0.034215709 |
| ENSG00000 | - | 16 | 67550815 | 67552935 | - | 2121 | 0.05 | 0.29 | 0.02 | 0 | 0 | 0 | 0.04056699 | -4.62354997 | 0.01051794 | 0.03496894 |
| ENSG00000 | YWHAZP2 | 2 | 1.27E+08 | 1.27E+08 | - | 728 | 0.07 | 0 | 0.21 | 0.61 | 0.61 | 0.72 | 6.67411065 | 2.738575605 | 0.010536549 | 0.035007398 |
| ENSG00000 | CLXN | 8 | 48710789 | 48735311 | - | 5822 | 0.14 | 0.07 | 0.07 | 0.25 | 0.17 | 0.26 | 2.34511078 | 1.229656076 | 0.010628486 | 0.035265716 |
| ENSG00000 | CFAP54 | 12 | 96489571 | 96875555 | + | 12914 | 0.11 | 0.05 | 0.05 | 0.12 | 0.15 | 0.17 | 2.03229195 | 1.023107667 | 0.01063666 | 0.035281063 |
| ENSG00000 | ZNF385B | 2 | 1.79E+08 | 1.8E+08 | - | 5417 | 0.05 | 0.07 | 0.02 | 0.19 | 0.1 | 0.16 | 3.2821091 | 1.714623198 | 0.010694881 | 0.03546235 |
| ENSG00000 | - | 5 | 96784777 | 96785999 | + | 1001 | 0.7 | 1.71 | 0.98 | 0.44 | 0.44 | 0.47 | 0.38588878 | -1.373742996 | 0.010714169 | 0.035520382 |
| ENSG00000 | UCA1 | 19 | 15828206 | 15836328 | + | 4154 | 0 | 0.02 | 0.01 | 0.17 | 0.09 | 0.06 | 8.22610761 | 3.040209945 | 0.010743468 | 0.035581922 |
| ENSG00000 | - | 1 | 2E+08 | 2E+08 | - | 638 | 0 | 0.16 | 0 | 0.7 | 0.61 | 0.55 | 11.0963867 | 3.472018063 | 0.010983106 | 0.036275379 |
| ENSG00000 | TNFRSF11A | 18 | 62325287 | 62391288 | + | 8989 | 0.07 | 0.05 | 0.03 | 0.12 | 0.15 | 0.12 | 2.4656179 | 1.301949243 | 0.010998498 | 0.03630283 |
| ENSG00000 | - | 13 | 1.03E+08 | 1.03E+08 | + | 867 | 0.75 | 0.42 | 0.89 | 0.15 | 0.06 | 0.27 | 0.22367324 | -2.1605354 | 0.011134413 | 0.036687292 |
| ENSG00000 | EHF | 11 | 34621093 | 34663288 | + | 6617 | 0.05 | 0.05 | 0.1 | 0.14 | 0.22 | 0.16 | 2.50548937 | 1.325092419 | 0.01128013 | 0.037087531 |
| ENSG00000 | RPL29P11 | 3 | 37016523 | 37017014 | - | 492 | 1.21 | 1.68 | 0.73 | 2.84 | 3.27 | 2.37 | 2.24781251 | 1.168521706 | 0.011355018 | 0.037309078 |
| ENSG00000 | LINC01238 | 2 | 2.42E+08 | 2.42E+08 | + | 2854 | 0.07 | 0.22 | 0.05 | 0 | 0.02 | 0 | 0.0626123 | -3.997410068 | 0.011425842 | 0.037498409 |
| ENSG00000 | - | 7 | 1.52E+08 | 1.52E+08 | + | 1554 | 0.03 | 0.23 | 0 | 0.41 | 0.36 | 0.49 | 4.56875972 | 2.19180257 | 0.011477136 | 0.037654322 |
| ENSG00000 | F8A3 | X | 1.55E+08 | 1.55E+08 | - | 1707 | 0.58 | 0.27 | 0.48 | 0.07 | 0.13 | 0.2 | 0.296073 | -1.755975147 | 0.011536692 | 0.037812282 |
| ENSG00000 | TFPI2-DT | 7 | 93890913 | 93893601 | + | 527 | 0.09 | 0 | 0 | 1.33 | 0.32 | 0.22 | 17.0751151 | 4.093823395 | 0.011558958 | 0.037854064 |
| ENSG00000 | SLC38A5 | X | 48458537 | 48470260 | - | 4017 | 0.2 | 0.24 | 0.19 | 0.05 | 0.12 | 0.04 | 0.32973853 | -1.600605634 | 0.011611339 | 0.038006826 |
| ENSG00000 | PSG1 | 19 | 42866464 | 42879822 | - | 4961 | 0.29 | 0.19 | 0.32 | 0.06 | 0.19 | 0.09 | 0.42294457 | -1.241459511 | 0.011638557 | 0.038083382 |
| ENSG00000 | CHRM3 | 1 | 2.39E+08 | 2.4E+08 | + | 15173 | 0.08 | 0.1 | 0.14 | 0.03 | 0.04 | 0.08 | 0.46667811 | -1.099500282 | 0.011655425 | 0.038119756 |
| ENSG00000 | BEAN1-AS1 | 16 | 66469796 | 66481230 | - | 1728 | 0.14 | 0.27 | 0.27 | 0.04 | 0 | 0.03 | 0.09722772 | -3.362488435 | 0.011721945 | 0.038286938 |
| ENSG00000 | DMRT1 | 9 | 841690 | 969090 | + | 3253 | 0.08 | 0.11 | 0.08 | 0.25 | 0.36 | 0.2 | 2.9222105 | 1.547060106 | 0.01192413 | 0.038877081 |
| ENSG00000 | - | 22 | 15823197 | 15823890 | + | 694 | 0.43 | 1.42 | 0.45 | 0.09 | 0.08 | 0.25 | 0.17835694 | -2.487160772 | 0.012012667 | 0.039133665 |
| ENSG00000 | RUSF1-DT | 16 | 31508464 | 31509566 | + | 1103 | 0.59 | 0.19 | 0.42 | 1.32 | 0.65 | 1.22 | 2.55590296 | 1.35383306 | 0.01208804 | 0.039353419 |
| ENSG00000 | - | 16 | 70661667 | 70665836 | + | 4170 | 0.13 | 0.09 | 0.26 | 0.03 | 0.05 | 0.04 | 0.25535435 | -1.96942746 | 0.012140687 | 0.039498948 |
| ENSG00000 | - | 4 | 1.05E+08 | 1.05E+08 | - | 549 | 2.81 | 4.15 | 2.35 | 1.39 | 1.21 | 2.12 | 0.48885099 | -1.032533307 | 0.012191637 | 0.03964525 |
| ENSG00000 | - | 21 | 25515349 | 25518865 | - | 1431 | 0.28 | 0.18 | 0.25 | 0.53 | 0.82 | 0.65 | 2.7086539 | 1.437576062 | 0.012198205 | 0.039660123 |
| ENSG00000 | ABHD11-AS1 | 7 | 73734994 | 73736161 | + | 757 | 0.2 | 0.14 | 0.61 | 1.26 | 0.81 | 1.15 | 3.27731356 | 1.712513711 | 0.012213472 | 0.039696779 |
| ENSG00000 | - | 22 | 35298838 | 35299541 | - | 385 | 0.52 | 0.94 | 0.94 | 0.16 | 0 | 0 | 0.06628995 | -3.91506612 | 0.012317136 | 0.039974353 |
| ENSG00000 | BMP7 | 20 | 57168753 | 57266641 | - | 5425 | 0.02 | 0.01 | 0 | 0.04 | 0.08 | 0.12 | 8.08125124 | 3.014578687 | 0.012430177 | 0.040256362 |
| ENSG00000 | TET1 | 10 | 68560337 | 68694487 | + | 9612 | 0.09 | 0.08 | 0.09 | 0.02 | 0.05 | 0.02 | 0.33764429 | -1.566423948 | 0.012558713 | 0.040606504 |
| ENSG00000 | LINC00971 | 3 | 84638405 | 84881924 | - | 8725 | 0.01 | 0 | 0.01 | 0.04 | 0.04 | 0.04 | 10.5777281 | 3.40295789 | 0.012606474 | 0.040741058 |
| ENSG00000 | - | 14 | 19072333 | 19075341 | + | 2144 | 0.14 | 0.19 | 0.1 | 0 | 0.03 | 0 | 0.06637795 | -3.913152139 | 0.012624209 | 0.040791744 |
| ENSG00000 | - | 15 | 52017167 | 52018032 | - | 866 | 0.75 | 0.72 | 0.77 | 1.32 | 1.15 | 2.69 | 2.22562186 | 1.154208492 | 0.012631439 | 0.040808475 |
| ENSG00000 | NINJ2-AS1 | 12 | 630858 | 664196 | + | 4356 | 0.17 | 0.04 | 0.08 | 0.34 | 0.24 | 0.21 | 2.60602592 | 1.381851434 | 0.012642674 | 0.040824875 |
| ENSG00000 | PAGE1 | X | 49687447 | 49695984 | - | 659 | 0.23 | 0.24 | 0.08 | 1.73 | 0.76 | 0.27 | 4.86000757 | 2.280958561 | 0.012726448 | 0.041042074 |
| ENSG00000 | LNCOG | 12 | 76259836 | 76305969 | + | 2108 | 0.19 | 0.29 | 0.22 | 0.45 | 0.74 | 0.53 | 2.35550005 | 1.236033363 | 0.012754551 | 0.041119364 |
| ENSG00000 | GLUD2 | X | 1.21E+08 | 1.21E+08 | + | 2485 | 0.22 | 0.21 | 0.21 | 0.64 | 0.42 | 0.45 | 2.27411218 | 1.185303422 | 0.013250525 | 0.042532153 |
| ENSG00000 | BAMBI | 10 | 28677510 | 28682932 | + | 2139 | 0.14 | 0.29 | 0.31 | 0.59 | 0.6 | 0.57 | 2.28999015 | 1.195341395 | 0.013256007 | 0.042536018 |
| ENSG00000 | EHBP1-AS1 | 2 | 62957326 | 63048640 | - | 3367 | 0.35 | 0.23 | 0.38 | 0.15 | 0.08 | 0.17 | 0.40214798 | -1.314201612 | 0.01329242 | 0.042639099 |
| ENSG00000 | - | 18 | 1883524 | 2489426 | - | 6656 | 0.13 | 0.23 | 0.16 | 0.1 | 0.08 | 0.05 | 0.41503495 | -1.268695275 | 0.013343537 | 0.042782365 |

| | | | | | | | | | | | | | | | | |
|---|---|---|---|---|---|---|---|---|---|---|---|---|---|---|---|---|
| ENSG00000 | RPL37P1 | 20 | 35588324 | 35588607 | + | 284 | 1.4 | 0.55 | 1.27 | 0 | 0.2 | 0 | 0.06671537 | -3.905837075 | 0.013380623 | 0.042894357 |
| ENSG00000 | - | 17 | 38749360 | 38751773 | + | 2414 | 0.14 | 0.19 | 0.19 | 0.45 | 0.37 | 0.53 | 2.44946573 | 1.292467109 | 0.013390153 | 0.042911071 |
| ENSG00000 | FAM30A | 14 | 1.06E+08 | 1.06E+08 | + | 10311 | 0.14 | 0.14 | 0.11 | 0.04 | 0.11 | 0.01 | 0.40225449 | -1.313819553 | 0.013427463 | 0.043023702 |
| ENSG00000 | - | 18 | 51019401 | 51030050 | - | 358 | 2.78 | 0.14 | 1.15 | 0 | 0.47 | 0 | 0.11342445 | -3.140196385 | 0.013523258 | 0.04330971 |
| ENSG00000 | ARAP2 | 4 | 35948221 | 36244514 | - | 12427 | 0.08 | 0.1 | 0.27 | 0.37 | 0.37 | 0.42 | 2.43952639 | 1.286601088 | 0.01358171 | 0.043468906 |
| ENSG00000 | SH3TC2 | 5 | 1.49E+08 | 1.49E+08 | - | 38112 | 0.02 | 0.03 | 0.01 | 0.03 | 0.06 | 0.04 | 2.10386042 | 1.073038992 | 0.013732226 | 0.043865913 |
| ENSG00000 | - | 11 | 3084393 | 3085443 | - | 730 | 0.2 | 0.78 | 0.28 | 0 | 0.08 | 0 | 0.06605535 | -3.920180744 | 0.01399517 | 0.044555547 |
| ENSG00000 | TMEM151B | 6 | 44270450 | 44307506 | + | 5475 | 0.06 | 0.05 | 0.06 | 0.23 | 0.11 | 0.15 | 2.81395745 | 1.492600515 | 0.014071681 | 0.044756138 |
| ENSG00000 | COP1-DT | 1 | 1.76E+08 | 1.76E+08 | + | 3821 | 0.05 | 0.04 | 0.03 | 0.2 | 0.22 | 0.08 | 3.95044912 | 1.982016681 | 0.014154504 | 0.044990779 |
| ENSG00000 | - | 20 | 23179272 | 23190419 | + | 5313 | 0.07 | 0.1 | 0.08 | 0.19 | 0.24 | 0.18 | 2.43395712 | 1.283303749 | 0.014203929 | 0.045108616 |
| ENSG00000 | LINC01431 | 20 | 23356586 | 23358175 | - | 1590 | 0.53 | 0.52 | 0.32 | 0.08 | 0.17 | 0.18 | 0.30839723 | -1.697138267 | 0.014242815 | 0.045213661 |
| ENSG00000 | CTXND1 | 15 | 80195481 | 80252213 | - | 6893 | 0.1 | 0.09 | 0.07 | 0 | 0.01 | 0.05 | 0.22152985 | -2.174426971 | 0.014276867 | 0.045300065 |
| ENSG00000 | ENAM | 4 | 70628744 | 70646824 | + | 5807 | 0.02 | 0 | 0 | 0.11 | 0.05 | 0.04 | 10.8026357 | 3.433311454 | 0.014380763 | 0.045578813 |
| ENSG00000 | CPN2 | 3 | 1.94E+08 | 1.94E+08 | - | 3025 | 0.12 | 0.22 | 0.14 | 0.44 | 0.33 | 0.39 | 2.34795151 | 1.231402611 | 0.014573581 | 0.046101758 |
| ENSG00000 | CACNB2 | 10 | 18140424 | 18543557 | + | 11162 | 0.15 | 0.09 | 0.14 | 0.02 | 0.09 | 0.06 | 0.43608047 | -1.197333716 | 0.014711901 | 0.046480165 |
| ENSG00000 | - | 5 | 1.52E+08 | 1.52E+08 | + | 3317 | 0.33 | 0.19 | 0.2 | 0.06 | 0 | 0.16 | 0.28703396 | -1.800706637 | 0.014801059 | 0.046717312 |
| ENSG00000 | SNHG22 | 18 | 49813965 | 49851059 | + | 2662 | 0.34 | 0.21 | 0.19 | 0.05 | 0.1 | 0.07 | 0.2836568 | -1.817781635 | 0.014871222 | 0.046886674 |
| ENSG00000 | - | 19 | 49050217 | 49050846 | - | 630 | 0.08 | 0 | 0.16 | 0.6 | 0.97 | 0.37 | 7.71923495 | 2.94845787 | 0.014877307 | 0.046898422 |
| ENSG00000 | SDCBP2 | 20 | 1309909 | 1329139 | - | 2553 | 0.02 | 0.02 | 0.06 | 0.17 | 0.17 | 0.21 | 5.33215466 | 2.414718625 | 0.015016228 | 0.047238995 |
| ENSG00000 | - | 21 | 6228966 | 6267317 | - | 2878 | 0 | 0.02 | 0 | 0 | 0.12 | 0.22 | 17.2231739 | 4.106279125 | 0.015105616 | 0.047467631 |
| ENSG00000 | TRIM7-AS2 | 5 | 1.81E+08 | 1.81E+08 | + | 664 | 1.8 | 3.67 | 1.47 | 1.15 | 0.75 | 1.4 | 0.45747001 | -1.128250918 | 0.015135815 | 0.047522872 |
| ENSG00000 | CACNA1E | 1 | 1.81E+08 | 1.82E+08 | + | 21561 | 0.05 | 0.06 | 0.04 | 0.01 | 0.03 | 0.02 | 0.40913381 | -1.289355332 | 0.015189447 | 0.04763567 |
| ENSG00000 | GLYATL1 | 11 | 58905398 | 59043527 | + | 5440 | 0.05 | 0.01 | 0.02 | 0.21 | 0.11 | 0.04 | 4.15725105 | 2.055629872 | 0.015469987 | 0.048467101 |
| ENSG00000 | VASH2 | 1 | 2.13E+08 | 2.13E+08 | + | 9086 | 0.08 | 0.08 | 0.07 | 0.02 | 0.03 | 0.02 | 0.30452841 | -1.715351252 | 0.015499991 | 0.048531316 |
| ENSG00000 | SSMEM1 | 7 | 1.3E+08 | 1.3E+08 | + | 1368 | 0.18 | 0.11 | 0.08 | 0.65 | 0.2 | 0.6 | 3.7316849 | 1.899827172 | 0.015935033 | 0.049728206 |

**Supplementary Table 5. Gene Set Enrichment Analysis (GSEA) identifies hallmark pathways suppressed by circSPECC1 knockdown in pGBM1 cells**

| ID | setSize | enrichmentS | NES | pvalue | p.adjust | qvalue | rank | leading_edge | core_enrichment |
|---|---|---|---|---|---|---|---|---|---|
| HALLMARK_HYPOXIA | 21 | -0.55448629 | -2.3317497 | 0.000376677 | 0.005273475 | 0.004361521 | 211 | tags=81%, lis | SLC2A1/IGFBP1/TMEM45A/TGFBI/FOS/ENO2/ADM/ALDOC/ERO1A/NDRG1/DPYSL4/PPFIA4/ANGPTL4/COL5A1/IGFBP3/CCN5/PKP1 |
| HALLMARK_GLYCOLYSIS | 15 | -0.53429415 | -1.97351726 | 0.004742842 | 0.033199894 | 0.027458559 | 237 | tags=87%, lis | PGK1/PLOD2/ARTN/TGFBI/SLC16A3/ENO2/SPAG4/ERO1A/DPYSL4/PPFIA4/ANGPTL4/COL5A1/IGFBP3 |
| HALLMARK_TNFA_SIGNALING_VIA_ | 17 | 0.42808587 | 1.590687188 | 0.032454993 | 0.151456634 | 0.125264885 | 216 | tags=65%, lis | IL1B/SLC16A6/SERPINB2/BCL2A1/CCL20/IL1A/GCH1/CXCL3/CXCL2/CXCL1/LIF |

**Supplementary Table 6. Information of antibodies used in the study**

| Antibody | Company | Category Number | Dilution |
|---|---|---|---|
| N-cadherin | Cell Signaling Technology | 4061 | WB: 1:1000 |
| CD44 | Proteintech Group | 15675-1-AP | WB: 1:1000 |
| Vimentin | Cell Signaling Technology | 5741 | WB: 1:1000 |
| Snail | Cell Signaling Technology | 3879 | WB: 1:1000 |
| Slug | Cell Signaling Technology | 9585 | WB: 1:1000 |
| PGK1 | Proteintech Group | 17811-1-AP | WB: 1:10000 |
| Tubulin | Ray Antibody Biotech | RM2003 | WB: 1:5000 |
| IGF2BP2 | Proteintech Group | 11601-1-AP | WB:1:2000; IF:1：50 |
