# Supplementary material for: Hypoxia-induced circSPECC1 drives temozolomide resistance in glioblastoma via IGF2BP2-mediated PGK1 mRNA stabilization
Source: Cell Death Dis. 2026 Jun 17;17(1):756. doi: 10.1038/s41419-026-08879-8 (PMC13518933; doi:10.1038/s41419-026-08879-8)

Figure 4

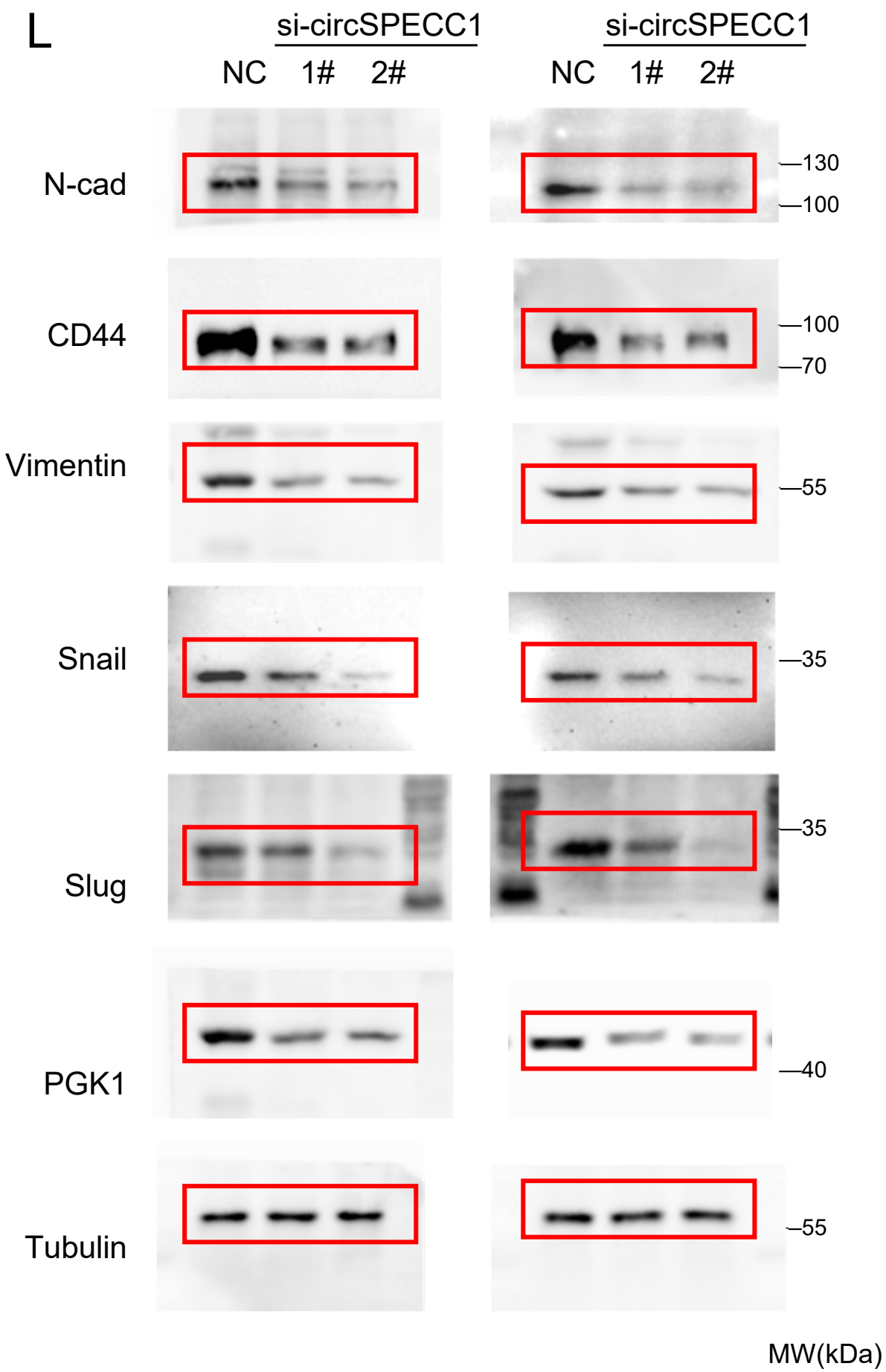

Figure 5

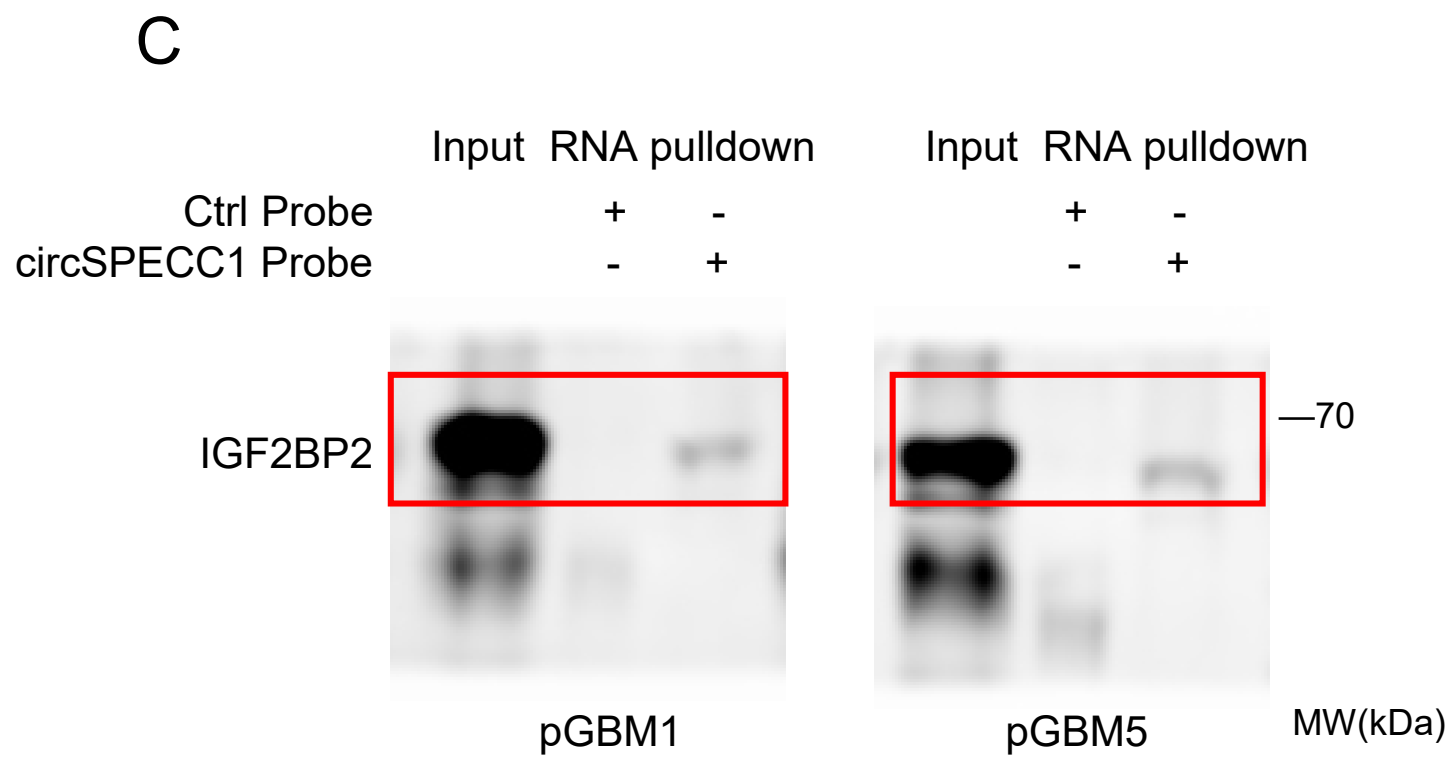

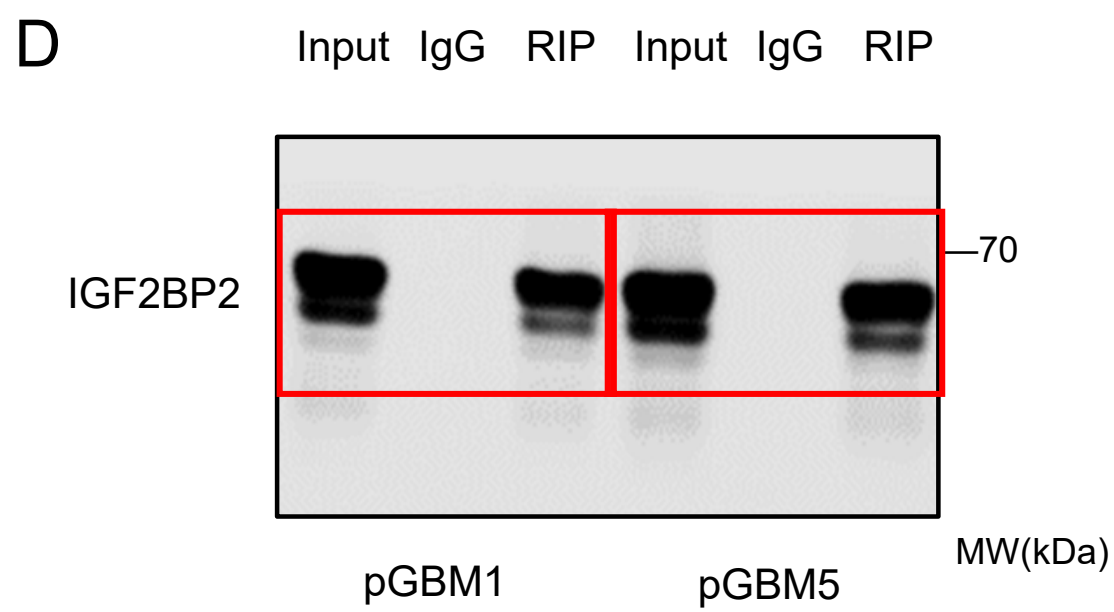

Figure 6

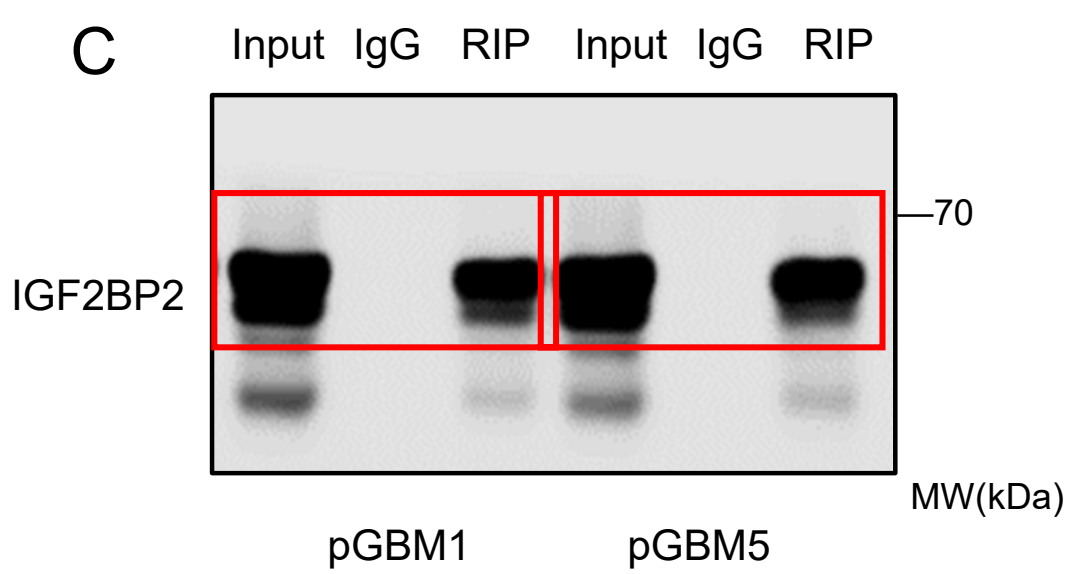

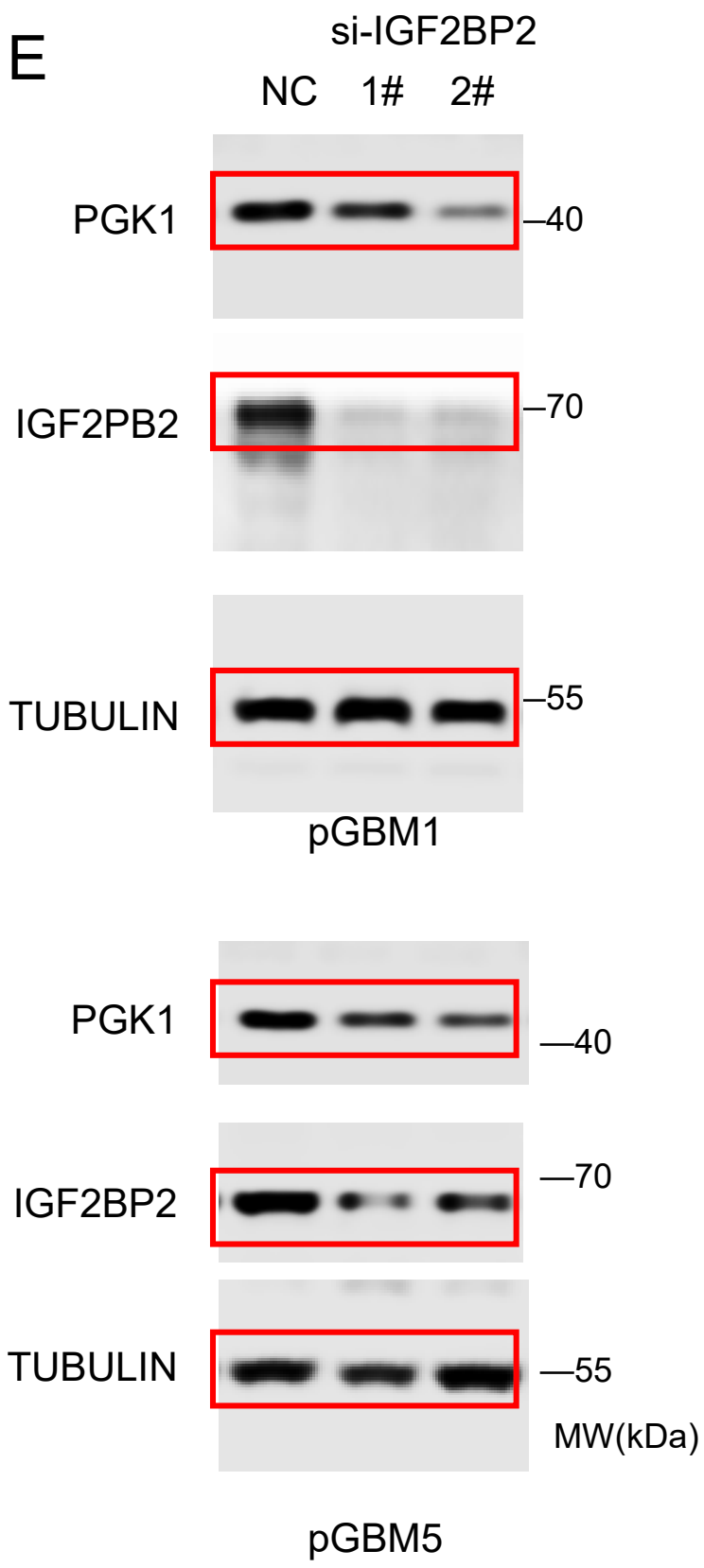

Figure 7

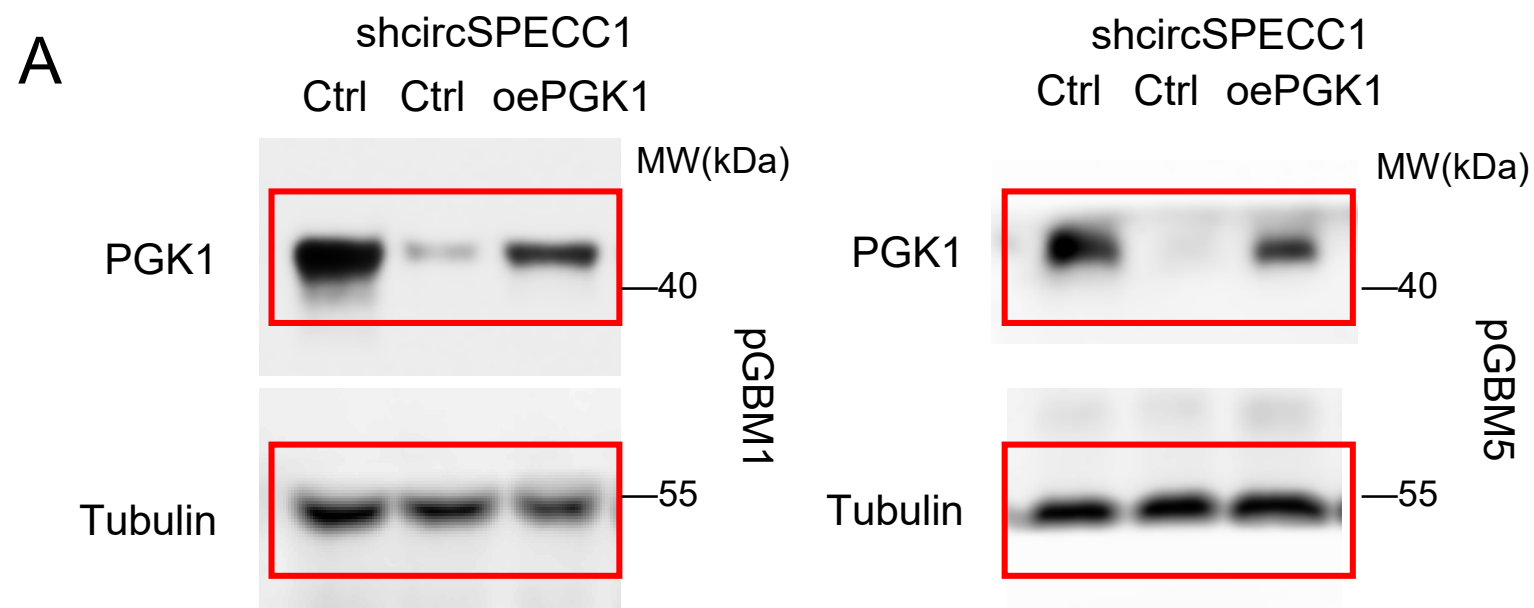

Supplement: Supplementary file 2 [file 41419_2026_8879_MOESM3_ESM.pdf]
