# Supplementary material for: Hypoxia-induced circSPECC1 drives temozolomide resistance in glioblastoma via IGF2BP2-mediated PGK1 mRNA stabilization
Source: Cell Death Dis. 2026 Jun 17;17(1):756. doi: 10.1038/s41419-026-08879-8 (PMC13518933; doi:10.1038/s41419-026-08879-8)
Supplement: Supplementary file 3 [file 41419_2026_8879_MOESM1_ESM.docx]

# **Supplemental Materials**

# **Hypoxia-induced** **circSPECC1 drives temozolomide resistance in glioblastoma via IGF2BP2-mediated PGK1 mRNA stabilization**

Yu Zeng, Liqian Zhao, Tianshi Que, Li Zhou, Xiaosheng Yang, Xin Xu, Kaihua Cao, Xizhao Wang, Xuhui Wang, Wenchuan Zhang, Ming Chen

Supplemental Methods

1.1 Quantitative real-time PCR (qRT-PCR) and PCR electrophoresis

Total RNA was extracted from cultured cells or frozen tissues via TRIzol reagent (Invitrogen, USA) following the manufacturer's instructions. RNA purity was assessed by NanoDrop spectrophotometry (A260/A280 > 1.8). First-strand cDNA was synthesized from 1 µg of RNA using the PrimeScript First-Strand cDNA Synthesis Kit (Takara, Japan) with random hexamers.

qRT-PCR was performed using SYBR Green Master Mix (Takara, Japan) under standard cycling conditions. Divergent primers spanning back-splice junctions were used to amplify circRNAs, whereas convergent primers were used to amplify linear transcripts. For electrophoresis, PCR products were separated on 2% agarose gels, stained with ethidium bromide, and visualized using UV light (GelDoc XR+, Bio-Rad). Gene expression levels were normalized to ACTB. Primer sequences are listed in Supplementary Table 1.

1.2 mRNA stability assays

RNA stability was assessed by time-course analysis with actinomycin D (Act D) treatment. Cells at approximately 70 % confluence were treated with Act D (5 µg/mL; Sigma‒Aldrich) to block de novo RNA transcription, and harvested at 0, 6, 12, and 24 h. Total RNA was isolated and transcript abundance was quantified by qRT-PCR as described above.

1.3 Dual-luciferase reporter assay

For dual-luciferase reporter assays, wild-type promoter fragments of the SPECC1 were PCR-amplified and cloned downstream of firefly luciferase in the psiCHECK-2 vector. 4 × 10⁴ HEK293T cells per well were seeded in 24-well plates and, 24 h later, co-transfected with 200 ng reporter plasmid or/and 200 ng HIF1A overexpression plasmid using Lipofectamine 3000 (Invitrogen). A Renilla luciferase-expressing pRL-TK plasmid (10 ng) served as internal control. After 36 h, cells were lysed in Passive Lysis Buffer and luciferase activities measured sequentially with the Dual Luciferase Reporter Gene Assay Kit (11402ES60; Yeasen, China) on luminometer. Firefly values were normalized to Renilla values, and relative luciferase activity was expressed as fold change over the matched empty-vector or scramble control. Each experiment was performed in quadruplicate and repeated three times.

1.4 CCK8 assay and temozolomide sensitivity assessment

Cell viability and temozolomide (TMZ) sensitivity were quantified using the Cell Counting Kit-8 (CCK-8; Biosharp, China). GBM Cells with indicated treatments were seeded in 96-well plates at 3000–5000 cells/well and allowed to adhere overnight. At the specified time points (1, 3, 5 or 7 day), 10 µl CCK-8 reagent was added to each well and plates were incubated for 1 hour at 37 °C in a humidified 5 % CO₂ incubator, and absorbance was measured at 450 nm using a microplate reader. For TMZ dose-response assays, cells were exposed to TMZ (0–1600 µM; Sigma-Aldrich) after adherence for 48 hours. CCK-8 reagent (10 µL/well) was added, incubated for 1 hour at 37 °C, and absorbance was measured at 450 nm. Half-maximal inhibitory concentration (IC_50_) was calculated via nonlinear regression of dose-response curves (https://www.aatbio.com/tools/ic50-calculator).

1.5 Clone formation assay

Colony formation capacity was assessed by seeding cells in 6-well plates at low densities (500 cells/well for GBM cells with indicated treatments). Cells were cultured for 14 days under standard conditions with or without temozolomide with IC_50_ concentration of indicated cells, with medium refreshed every 3 days. Colonies were then fixed with 4 % paraformaldehyde, stained with 0.5 % crystal violet, and washed with PBS gently three times. Colonies containing ≥50 cells were manually counted. Data were normalized to untreated controls.

1.6 EdU Assay

DNA synthesis and cell proliferation were assessed using the Cell-Light EdU Apollo567 In Vitro Kit (C10310-1, RiboBio, China). Briefly, Cells with indicated treatments were seeded in 96-well plates (5,000 cells/well) for 24 hours, and culture medium was then replaced with that containing 50 μM EdU. After culture for 2 hours, cells were washed gently with PBS and fixed with 4 % paraformaldehyde, neutralized with glycine, permeabilized with 0.5 % Triton X-100, and incubated with Apollo reaction reagent (30 min, dark, room temperature). Nuclei were counterstained with Hoechst 33342 (5 μg/mL; Thermo Fisher). Images were acquired using a fluorescence microscope (Olympus IX73) at 200× magnification. EdU^+^cells (red) and total nuclei (blue) were quantified in five random fields per well using ImageJ, and proliferation rate was calculated as: EdU^+^ nuclei / total nuclei) × 100%.

1.7 Wound healing assay

For wound healing assays. Cells were seeded in 6-well plates at 5 × 10⁵ cells/well and grown to >90 % confluence. Sterile 200-μL pipette tips were used to create uniform linear wounds, followed by three PBS washes to remove debris. Wound closure was monitored at 0 and 24 hours under microscopy at 100× magnification. Five predetermined fields per wound were imaged at each time point. The Gap area was quantified using ImageJ, and migration rate (%) was calculated as: (Area_0h_ – Area_24h_) / Area_0h_ × 100 %.

1.8 Transwell and Boyden assay

For Transwell assays, 5 × 10⁴ serum-starved cells in 200 μl of medium lacking FBS were seeded in the upper compartment of the 24-well Transwell chambers (8-μm pore; Corning); for Boyden assays, chambers were pre-coated with 30 μg Matrigel (BD Biosciences). The lower chamber contained 600 μl medium plus 10 % FBS as chemoattractant. After 24 h incubation at 37 °C, 5 % CO₂, non-invading cells were removed with a cotton swab, and cells on the underside of the membrane were fixed with 4 % paraformaldehyde, stained with 0.5 % crystal violet for 30 min, and imaged on an inverted microscope (×200). Five random fields per membrane were counted using ImageJ. Results are expressed as mean cells/field ± SD from three independent experiments performed in duplicate.

1.9 Western Blot assay

Cells were lysed in ice-cold RIPA buffer (Beyotime, China) supplemented with 1 mM PMSF and a protease/phosphatase inhibitor cocktail (HY-K0013, MCE). Lysates were quantified via BCA assay (Beyotime, China), and equal amounts of protein (20–30 μg) were resolved on 10 % SDS-PAGE gels and transferred onto 0.45 μm PVDF membranes (Millipore). Membranes were blocked for 1 h at room temperature in 5 % non-fat milk in TBS-Tween 0.1 % (TBST), then incubated overnight at 4 °C with primary antibodies (1:1000 dilution unless specified). After three washes in TBST, blots were probed with HRP-conjugated secondary antibodies (1:5000, Ray Antibody) for 1 h at room temperature and visualized with ECL reagent.

1.10 RNA pulldown assay and mass spectrum

Biotin-labeled probes targeting the back-splice junction of circSPECC1 were synthesized by GenePharma. RNA pulldown assay was performed with RNA-Protein pull-down kit (P0201, GEENSEED, China) according to manufacturer’s protocol. Briefly, cell lysates from GBM lines were incubated with 50 pmol biotinylated probes and rotated at 4 °C for 1 hour. Complexes were captured with Streptavidin Magnetic Beads, washed five times with lysis buffer, and eluted in loading buffer. Bound proteins were examined using liquid chromatography‒tandem MS and validated using Western blotting. The data were processed using MaxQuant (v2.0.3) against the UniProt human database.

1.11 RNA immunoprecipitation (RIP)

Cells were lysed in lysis buffer supplemented with RNase/protease inhibitors. Cell lysates were then incubated 5 μg of anti-IGF2BP1 or match IgG control antibody overnight at 4 °C. Antibody-RNA complexes were captured using Protein A/G magnetic beads (HY-K0202, MCE; 1 hour, 4 °C), washed five times with lysis buffer, and digested with Proteinase K. Co-precipitated RNA was purified using TRIzol LS followed by ethanol precipitation and analyzed by RT-qPCR. Enrichment was calculated as: % Input = 2^−(CTRIP – CTInput)^ × 100 %, normalized to IgG control.

1.12 Glycolysis stress assay

Glycolytic flux was quantified using the Seahorse XF Glycolysis Stress Test Kit (Agilent Technologies) on an XFe96 Analyzer. Cells were seeded in XF96 microplates at 5000 cells/well and incubated overnight. Prior to assay, cells were washed twice with unbuffered Seahorse XF DMEM (pH 7.4) supplemented with 2 mM glutamine, 1 mM pyruvate, and 10 mM glucose, followed by 1-hour equilibration at 37 °C (CO_2_-free). Glucose, oligomycin, and 2-deoxyglucose (2-DG) were sequentially injected at the indicated time points. Extracellular acidification rate (ECAR) was measured in mpH/min and normalized to cell count determined by Hoechst 33342 staining immediately after the run. Basal glycolysis, glycolytic capacity, and glycolytic reserve were measured according to the manufacturer's protocol. All assays were performed in quadruplicate wells and repeated three times.

1.13 Immunohistochemistry staining

Immunohistochemistry was performed on paraffin-embedded tissue sections. After deparaffinization and rehydration, slides were subjected to antigen retrieval in 10 mM sodium citrate (pH 6.0) at 95 °C for 20 min, cooled to room temperature. Endogenous peroxidase was quenched with 3% H_2_O_2_ for 15 min, followed by blocking with 5 % goat serum for 1 h. Sections were incubated with primary antibodies overnight at 4 °C in a humidified chamber. After gentle wash for three times, sections were incubated with HRP-conjugated secondary antibodies (PV-9001, ZSGB-Bio) for 30 min at room temperature, developed with DAB substrate (ZL1-9019, ZSGB-Bio), counterstained with hematoxylin, dehydrated, and mounted. Positive and negative controls were included in each run. Images were captured under microscope at 200× magnification and analyzed with ImageJ. Staining intensity was scored blindly as 0–3 (negative = 0; weak = 1; medium = 2; strong = 3) and multiplied by the percentage of positive cells (0: <5%; 1: 5–25%; 2: 26–50%; 3: 51–75%; 4: >75%) to generate staining index.

***1.14 Transcriptome sequencing and bioinformatic analysis***

Total RNA of pGBM1 cells transfected with shRNA targeting circSPECC1 was extracted and quantified NanoDrop spectrophotometry (A260/A280 > 1.8). Total RNA Library were prepared using Illumina TruSeq RNA Sample Preparation kit, according to manufacturer’s instruction. The paired-end reads were generated using the Illumina HiSeq instrument according to manufacturer’s instructions and then mapped to human genome (hg38) and normalized with *edgeR* package using TMM in R. Differential expression analysis and subsequent gene set enrichment analysis (GSEA) was performed with *DESeq2* and *clusterProfiler* package in R, respetively.
